# Identification and analysis of proline-rich proteins and hybrid proline-rich proteins super family genes from *Sorghum bicolor* and their expression patterns to abiotic stress and zinc stimuli

**DOI:** 10.3389/fpls.2022.952732

**Published:** 2022-09-26

**Authors:** Guddimalli Rajasheker, Marka Nagaraju, Rinku Polachirakkal Varghese, Naravula Jalaja, Anil Kumar Somanaboina, Prashant Singam, Chintala Ramakrishna, Suprasanna Penna, Nese Sreenivasulu, P. B. Kavi Kishor

**Affiliations:** ^1^Department of Genetics and Biotechnology, Osmania University, Hyderabad, India; ^2^Biochemistry Division, ICMR-National Institute of Nutrition, Hyderabad, India; ^3^Department of Biotechnology, Vignan’s Foundation for Science, Technology & Research (Deemed to be University), Vadlamudi, India; ^4^School of Biosciences and Technology, Vellore Institute of Technology, Vellore, India; ^5^Department of Environmental Sciences, GITAM University, Visakhapatnam, India; ^6^Nuclear Agriculture and Biotechnology, Bhabha Atomic Research Center, Mumbai, India; ^7^Consumer-driven Grain Quality and Nutrition Research Unit, International Rice Research Institute, Los Baños, Philippines

**Keywords:** proline-rich proteins, hybrid proline-rich proteins, gene expressions, abiotic stresses, *Sorghum bicolor*

## Abstract

Systematic genome-wide analysis of *Sorghum bicolor* revealed the identification of a total of 48 homologous genes comprising 21 proline-rich proteins (*PRPs*) and 27 hybrid proline-rich proteins (*HyPRPs*). Comprehensive scrutiny of these gene homologs was conducted for gene structure, phylogenetic investigations, chromosome mapping, and subcellular localization of proteins. Promoter analysis uncovered the regions rich with phosphorous- (BIHD), ammonium-, sulfur-responsive (SURE), and iron starvation-responsive (IRO2) along with biotic, abiotic, and development-specific *cis*-elements. Further, PRPs exhibit more methylation and acetylation sites in comparison with HyPRPs. miRNAs have been predicted which might play a role in cleavage and translation inhibition. Several of the *SbPRP* genes were stimulated in a tissue-specific manner under drought, salt, heat, and cold stresses. Additionally, exposure of plants to abscisic acid (ABA) and zinc (Zn) also triggered PRP genes in a tissue-dependent way. Among them, *SbPRP17* has been found upregulated markedly in all tissues irrespective of the stress imposed. The expressions of *SbHyPRPs*, especially *SbHyPRP2*, *SbHyPRP6*, and *SbHyPRP17* were activated under all stresses in all three tissues. On the other hand, *SbHyPRP8* (root only) and *SbHyPRP12* (all three tissues) were highly responsive to cold stress and ABA while *SbHyPRP26* was induced by drought and Zn in the stem. Taken together, this study indicates the critical roles that *SbPRPs* and *SbHyPRPs* play during diverse abiotic stress conditions and notably the plausible roles that these genes play upon exposure to zinc, the crucial micronutrient in plants.

## Introduction

Plants are constantly exposed to both biotic and abiotic stresses because of which a lion’s share of the final productivity is lost globally. Plants can cope with them by deploying several complex physiological and molecular mechanisms depending upon the type, severity, and duration of stress. But unraveling the underlying mechanisms is crucial for us to design crops with superior tolerance to stresses. Extreme environmental conditions cause membrane damage, denaturation of proteins, and accumulation of excessive reactive oxygen species (ROS) that eventually dismantle the cellular fabric (Bokszczanin, 2013; Wang et al., 2019). To combat these stress-induced damages, plants accumulate osmotic agents, antioxidants, and also antioxidative enzymatic machinery (Bohnert et al., 2006; Bokszczanin, 2013). Plant cell wall proteins consist of both structural [arabinogalactan proteins, (AGPs) and extensins or hydroxyproline-rich *O*-glycoproteins (HRGPs)] and enzymatic proteins. They play key roles in cell wall formation, cell differentiation, development, and oxidative cross-linking mediated by extension peroxidases (McNeil et al., 1984; Jose-Estanyol and Puigdomenech, 1993; Showalter, 1993; Sommer-Knudsen et al., 1998). Proline rich-proteins (PRPs) and hybrid proline-rich proteins (HyPRPs) contain proline and hydroxyproline and are a part of the HRGPs super family proteins, widely distributed in plants and implicated in biotic and abiotic stress tolerance. PRPs contain at least two consecutive proline residues organized in the peptides and the domains contain 70% of proline residues (Zhang et al., 2021). Based on the presence or absence of N-terminal signal peptides and also differences in the domains, PRPs are divided into (1) PRPs that have two domains with signal peptides and highly rich repeat regions of proline at the N-terminus (Dvorakova et al., 2007), (2) PRPs that have three domains with N-terminal signal peptide, repeat regions of proline, aside cysteine-rich regions in the C-terminal end, and (3) PRPs that are devoid of any signal peptide, but with similar PPVYK repeat sequences at the C-terminus (Jose-Estanyol and Puigdomenech, 1994, 2000). The presence of signal peptides in these proteins infers that they are secreted-type of proteins in plants (Keller, 1993; Showalter, 1993; Cassab, 1998). The signal peptides permit their sorting into the rough endoplasmic reticulum and other cellular organelles. Newman and Cooper (2011) identified 31 distinct proline-rich tandem repeat protein classes targeted for secretion using a systems-level computational approach. Further, PRPs have been classified as an 8-cysteine motif or 8CM proteins, with 90–100 amino acids but the proteins are not integrated within the membrane. The 8-cysteine residues appear indispensable for the three-dimensional structure of these proteins. The 8-cysteine residues have a versatile structure in plant proteins domain and lipid transfer proteins (Jose-Estanyol et al., 2004; Dvorakova et al., 2012). In general, proline-rich repeats precede the 8CM, and such proteins are named as HyPRPs, but the N-terminus features like that of PRPs (Jose-Estanyol and Puigdomenech, 1994, 2000). HyPRPs differ from that of PRPs in the amino acid motif size and composition of proline repeats (Jose-Estanyol et al., 2004; Dvorakova et al., 2007). HyPRPs are subdivided into two groups based on cysteine residue positions in the peptidic sequence (Jose-Estanyol and Puigdomenech, 1994). Proline-rich domains of SbrHyPRP from *Solanum brevidens* contains repeated motifs PPHVKPPSTPK and PTPPIVSPP extended with TPKYP and TPKPPS motifs at N- or C-termini, respectively (Fischer et al., 2002). PRPs having tandem repeats of a hexapeptide PPPVHL were first characterized by corn endosperm (Esen et al., 1982). Function, localization, and differential expression of three PRPs from *Glycine max* (PRP1, PRP2, and PRP3) were studied (Hong et al., 1990; Wyatt et al., 1992). PRPs and PRP-like proteins have been found to play a crucial role during plant growth, germination of seeds (Bouton et al., 2005; Chen et al., 2014), and elongation of root hairs in *Arabidopsis* (Boron et al., 2014). Blocking *OsPRP3* by RNAi leads to defects in floral organogenesis in *Oryza sativa* implying its role during plant development (Gothandam et al., 2010). Further, PRPs modulate both biotic and abiotic stresses such as chilling, drought, and salinity stresses (Zhan et al., 2012; Kavi Kishor et al., 2015). Pathogen-derived molecules usually trigger systemic acquired resistance (SAR) in plants, which can be abolished in a wide spectrum of pathogens. Many experiments have shown that HyPRPs are closely related to biotic stress resistance (Yu et al., 2013; Cecchini et al., 2015, 2021). HyPRPs with plastid pools modulates systemic immunity either positively or negatively against *Pseudomonas syringae* (Banday et al., 2022). It appears that HyPRPs are obligatory for both SAR and induced-systemic resistance (ISR), and SAR signal movement or action (Gao et al., 2015; Cecchini et al., 2019, 2021; Kachroo and Kachroo, 2020). Therefore, HyPRPs have distinct roles to play in plant immunity. Also, when useful bacteria attack the roots of plants, ISR occurs, which leads to the transport of mobile signals from the tissues or organs that are immunized (Cecchini et al., 2015, 2019; Carella, 2020; Kachroo and Kachroo, 2020). Signal molecules such as azelaic acid (AZA) work as mobile signals for SAR, and such signals alongside free radicals are imperative in imparting SAR (Jung et al., 2009; Wang et al., 2014; Kachroo and Kachroo, 2020). The experiments infer that HyPRPs may have a critical role to play in biotic stress tolerance. In *Arabidopsis*, *FUSED OUTER CUTICULAR LEDGE1* (*FOCL1*) encodes a guard cell-expressed, secreted PRP protein. Interestingly, *focl1* mutants display enhanced drought tolerance compared to wild-type plants (Hunt et al., 2017). HyPRPs are poorly glycosylated cell wall glycoproteins and also play critical roles during plant ontogeny and abiotic stress tolerance like PRPs (Holk et al., 2002; Blanco-Portales et al., 2004; Kavi Kishor et al., 2015; Gujjar et al., 2019).

Information on the number of PRPs in diverse taxa is scanty. Showalter et al. (2016) discovered 49 *PtPRPs* in *Populus trichocarpa* and *Malus domestica*, 9 *MdPRP* genes have been identified (Zhang et al., 2021). Surprisingly, no HyPRPs have been found in algae, mosses, and ferns, but *Pinus taeda* contains 21 *PtHyPRPs* (Dvorakova et al., 2007). While in dicots such as *Arabidopsis thaliana* 28 *AtHyPRPs* have been found, 16 *StHyPRPs* were recorded in *Solanum tuberosum* (Dvorakova et al., 2012), 19 complete or nearly complete from *S. lycopersicon* (*SlHyPRPs*) (Kapoor et al., 2019) and 14 from *Medicago truncatula* (*MtHyPRPs*) have been recorded from the publicly available sequence data (Dvorakova et al., 2012). In *Glycine max*, 35 *GmHyPRP*-encoding genes were identified by Neto et al. (2013). In monocots, like *Oryza sativa* genome, conflicting reports exist. Studies by Dvorakova et al. (2007); Boutrot et al. (2008), and Kapoor et al. (2019) showed 21, 31, and 45 *OsHyPRP* genes, respectively, in rice whereas, in *Zea mays*, 52 members of *ZmHyPRPs* have been detected (Dvorakova et al., 2007). Such a large variation could be because of different methods that researchers have employed for identification.

Sorghum [*Sorghum bicolor* (L.) Moench] is an important C_4_ cereal crop and the fifth largest grown in arid and semi-arid regions, globally utilized for both food and fodder (FAOSTAT^1^). In the semi-arid tropics of Asia and Africa, it serves as a staple food, animal feed, fodder, and notably a promising multipurpose feedstock crop for bioethanol production (Silva et al., 2022). It is moderately tolerant to both drought and heat and requires very low inputs. Traits that impart alkalinity, salt, water deficit, waterlogging, and high-temperature stresses make this crop attractive for research purposes in comparison to other cereal crops (Hadebe et al., 2017; Huang, 2018). Fujino et al. (2014) have detected 21 *HyPRPs* in *Oryza sativa*, 12 in *Hordeum vulgare*, 10 in *Brachypodium*, 20 in *Zea mays*, and 28 in *S. bicolor* using a BLAST search of qLTG3-1 as the query. Though 28 *HyPRPs* have been reported in sorghum, their subcellular localizations, *cis*-element characterization, and their tissue-specific expressions and implications under abiotic stress conditions have not been undertaken. So, the number of PRPs and HyPRPs, their tissue-specific expressions under cold, high temperature, salt, drought stress conditions, and their response to ABA have not yet been elucidated. Similarly, little is known if these genes play a molecular function in response to soil nutrients especially sulfur (S), zinc (Zn), and iron (Fe). In the present study, we have presented results on the genome-wide analysis of sorghum for both PRP and HyPRP homolog genes, their characterization, and tissue-specific expressions under abiotic stress conditions besides their response to ABA and Zn.

## Materials and methods

### Plant material

Seeds of *S. bicolor* variety BTx623 (an inbred line) were obtained from ICRISAT, Patancheru, Hyderabad. This line is moderately tolerant to water deficit conditions and its genome sequencing information is publicly available. Seeds were sown in the pots filled with 5 kg of garden soil, and seedlings were grown for 40-days under glasshouse conditions (28/20°C day/night temperatures). Seedlings were subjected to drought stress by imposing a 200 mM mannitol solution, and salt stress was induced by saturating the seedlings with 200 mM NaCl solution for 4 h. Seedlings were subjected to cold stress by keeping them at 4°C and high-temperature stress by exposing them to 40°C for 4 h. Seedlings were also treated with 100 μM ABA and 25 mM zinc chloride solution (based on literature search) for 4 h separately. After completion of treatment, roots, stems, and leaves were collected along with respective controls (without treatment) and frozen immediately in liquid nitrogen and stored at –80°C refrigerator for isolation of RNA and gene expression analysis by qRT-PCR. Biological triplicates and technical duplicates were maintained for gene expression analysis for each treatment.

### *In silico* identification of proline-rich proteins and hybrid proline-rich proteins genes in sorghum

Proline-rich proteins and HyPRP gene sequences of *Oryza*, and *Arabidopsis* were retrieved from Plant GDB^2^ and Gramene database^3^ (Tello-Ruiz et al., 2021). These gene sequences were blasted against *S. bicolor* genome in Gramene database to find out their homologs. Genscan^4^ (Burge and Karlin, 1998) was used to retrieve genes and their respective protein sequences. Based on the homology, the identified putative protein sequences were subjected to motif search^5^ to check the reliability and for identifying the conserved domains (Letunic et al., 2004).

### *In silico* prediction of potential *cis*-regulatory elements

To predict the putative *cis*-acting elements of *PRP* and *HyPRP* promoter regions, the 2,000 bp genomic sequences upstream of the start codon were extracted and PLANTCARE (Lescot et al., 2002) software was used to identify putative *cis*-acting elements responsible for plant development and biotic and abiotic stress tolerance.

### Chromosomal localization, and gene structure analysis of sorghum *SbPRPs* and *SbHyPRPs*

The identified *SbPRP* and *SbHyPRP* genes were mapped to their respective chromosomes based on the information provided in the Gramene Genome Database. Gene Structure Display Server^6^ software was used for obtaining the gene structures of *SbPRPs* and *SbHyPRPs* – exons, introns, and untranslated sequence regions (UTRs) by aligning their gene and coding sequences (Guo et al., 2007).

### Synteny of proline-rich proteins and hybrid proline-rich proteins genes across *Sorghum*, *Oryza*, and *Arabidopsis*

TBtools (Chen et al., 2020) was used for identifying the synteny of *PRP* and *HyPRP* genes across *S. bicolor* (*Sb*), *O. sativa* (*Os*), and *A. thaliana* (*At*).

### Phylogenetic analysis and non-synonymous and synonymous substitution ratios of proline-rich proteins and hybrid proline-rich proteins

The Maximum Likelihood phylogenetic tree was constructed using MEGA 6.2 software (Tamura et al., 2013) by employing the Poisson correction, partial deletion, and bootstrap value (1,000 replicates) parameters to know the evolutionary relationships. The PAL2NAL^7^ (Suyama et al., 2006) software was used to calculate the substitution rates for non-synonymous and synonymous sites of each of the identified paralogs (sorghum) and orthologous gene pairs (between *Sorghum/Oryza*, and *Sorghum/Arabidopsis*) from phylogeny.

### Protein analysis of SbPRPs and SbHyPRPs

Molecular weight (MW), isoelectric point (pI), and GRAVY (grand average of hydropathy) of PRPs and HyPRPs were identified by using ProtParam Expasy tools^8^ (Gasteiger et al., 2005), while phosphorylation sites were predicted by employing NetPhos3.1 software of Expasy tools (Blom et al., 2004). The putative transmembrane helices within these genes were identified using TMHMM software (Moller et al., 2001). The sub-cellular localization of PRPs and HyPRPS was identified by the WOLFPSORT program^9^ (Horton et al., 2007). MEME software (Bailey and Gribskov, 1998) was employed to analyze new sequence patterns and their significance (Bailey et al., 2006). The software helped to identify the nature of motifs by setting different default parameters, number of motifs from 1 to 10 with a motif width of 5–50, and the number of motif sites from 5 to 10.

### Prediction of 3D structures and verification of their stability

The 3D structures for the highly expressed 12 proteins encoded by *SbPRP* (*SbPRP3*, *SbPRP6*, *SbPRP10*, *SbPRP12*, *SbPRP17*, and *SbPRP19*) and *SbHyPRP* genes (*SbHyPRP2*, *SbHyPRP5*, *SbHyPRP6*, *SbHyPRP13*, *SbHyPRP17*, and *SbHyPRP26*) were predicted using SWISS-MODEL server (Biasini et al., 2014). The predicted 3D structures of proteins for the highly expressed genes were evaluated for stability using PROCHECK and protein structure verification server (PSVS)^10^. The stability of the proteins was analyzed by Ramachandran plots through the calculation of phi (F) and psi (ψ) torsion angles.

### Analysis of acetylation, methylation sites, and miRNA target sites

The acetylation of the internal lysines was predicted by using PAIL^11^ (Li et al., 2006), the PRmePRed^12^ (Kumar et al., 2017) was used to predict the methylation sites. The putative miRNAs in targeting the *SbPRP and SbHyPRP* genes were identified using the psRNATarget server (Dai and Zhao, 2011) by default parameters.

### Prediction of simple sequence repeats

The simple sequence repeats (SSRs), the gene-specific molecular markers of PRPs and HyPRPs were identified within the transcripts by employing MISA-web^13^ (Beier et al., 2017).

### Digital expressions of *SbPRP* and *HyPRP* genes in diverse tissues and developmental stages under cold and drought stresses

Digital expression profiling of *SbPRP* and *SbHyPRP* genes was analyzed using Genevestigator^14^. *S. bicolor* mRNA-seq data available for all the 21 *SbPRP* and 27 *SbHyPRP* genes for stress conditions like cold and drought, three tissues, and five developmental stages were collected and used for analysis. Gene expression profiles were developed using hierarchical clustering and heat maps were generated for anatomy, development, and perturbations separately.

### qRT-PCR gene expression analysis of SbPRP and SbHyPRPs under diverse abiotic stress conditions

The transcriptional profiling of *PRPs* and *HyPRPs* was further investigated using qRT-PCR. Total RNA from root, stem, and leaf tissues under various stresses was extracted by using a nucleo-spin plant RNA isolation kit (MACHEREY-NAGEL) according to the manufacturer’s instructions. A total of 3 μg of total RNA was transcribed into cDNA by using first-strand synthesis kit (Thermo Scientific, Waltham, MA, USA). The SYBR Green Master Mix (2×) (Takara, Kasatsu, Shiga, Japan) was used according to the manufacturer’s recommendations. The qRT-PCR analysis was conducted by using ABI 7500 real-time PCR system (Applied Biosystems, Waltham, MA, USA) and Takara SYBR Green Master Mix (2 ×) with the following thermal cycles: 1 cycle at 95*^o^*C for 10 min, followed by 40 cycles alternatively at 95°C for 15 s and 60°C for 1 min. The amplicon dissociation curves were recorded with a fluorescence lamp after the 40th cycle by heating from 58 to 95*^o^*C within 20 min. Three biological replicates and two technical replicates were taken for the study. The gene expression data were normalized with Acyl Carrier Protein 2 (*SbACP2*) and Elongation Factor P (*SbEF-P*) genes of sorghum (Reddy et al., 2016). The *PRP* and *HyPRP* gene-specific primers used for qRT-PCR and reference genes are listed in Supplementary Table 1. The relative gene expressions were calculated by employing REST software (Pfaffl et al., 2002).

## Results

### Genome-wide characterization, chromosomal localization, and gene structure analysis of sorghum *PRPs* and *HyPRP* genes

**FIGURE 1:**
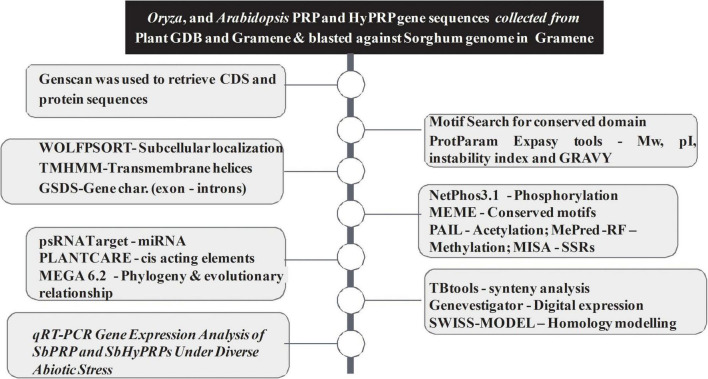
Methods, softwares, and pipelines used for genome-wide screening and characterization of SbPRPs and SbHyPRPs.

**FIGURE 2:**
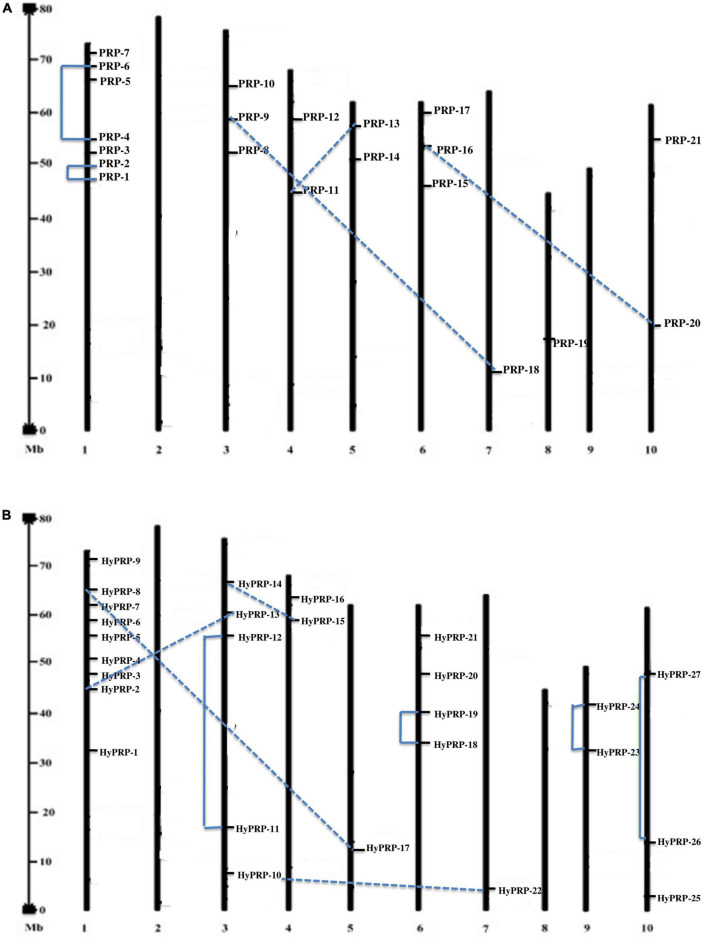
Location and duplications of SbPRP and SbHyPRP. **(A)** Location and duplication of *SbPRPs* on chromosomes. **(B)** Location and duplication of *SbHyPRPs* on chromosomes.

**FIGURE 3:**
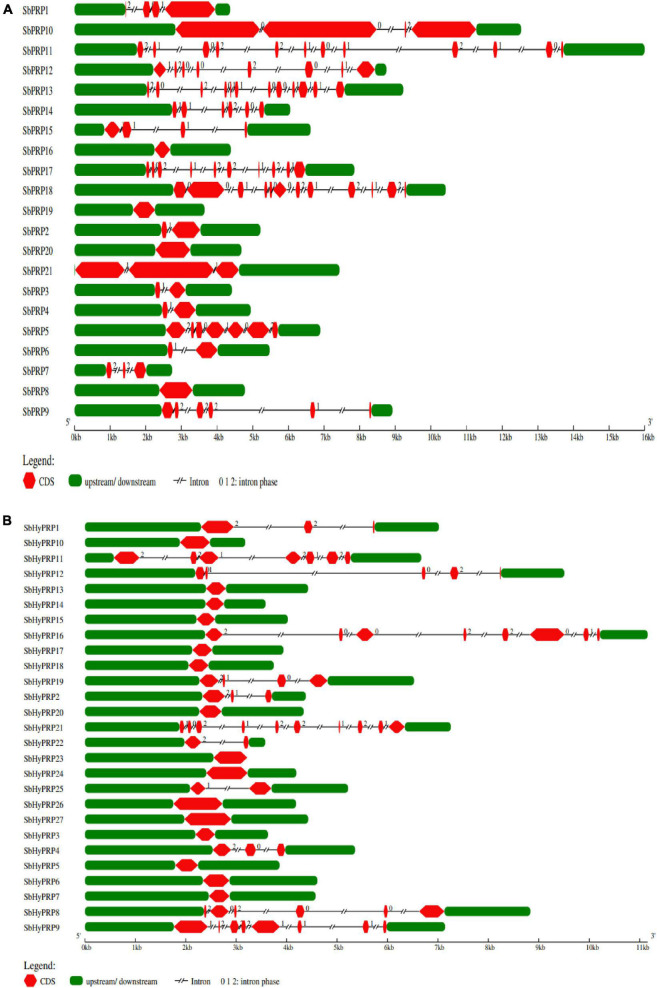
Gene characterization of SbPRP and SbHyPRP genes. **(A)** Distribution of exons, introns, upstream, and downstream regions in *SbPRPs*, **(B)** distribution of exons, introns, upstream, and downstream regions in *SbHyPRPs*.

The workflow of genome-wide screening and characterization of SbPRPs and SbHyPRPs is shown in Figure 1. Genome-wide analysis of sorghum resulted in the identification of a total of 48 homologous genes encompassing 21 *SbPRPs* and 27 *SbHyPRPs*. After checking their reliability by conserved domain search using the MOTIF search tool for convenience, the predicted PRPs, HyPRPs have been named as *SbPRP1* to *SbPRP21*, and *SbHyPRP1* to *SbHyPRP27*, respectively. Out of 21 *SbPRP* genes, 7 genes were localized on chromosome 1, followed by 3 and 6 (3 genes each). Chromosomes 4, 5, and 10 have 2 genes each, and chromosomes 7 and 8 have 1 gene each in *S. bicolor* (Table 1 and Figure 2A). Chromosome 1 also displays 9 HyPRP genes in *S. bicolor* out of 27 *SbHyPRPs*, followed by chromosomes 4 and 6 with 4 each, chromosome 3 with 3, chromosome 8 with 2, chromosome 5, 7, 10, 11, 12 with 1 gene each (Table 2 and Figure 2B). Chromosomes 2 and 9 totally lack the distribution of these genes. Thus, chromosome 1 has been found as the hot spot with 7 *SbPRP* and 8 *SbHyPRP* genes. The gene structure analysis provides possible mechanisms of structural evolution of *SbPRP* and *SbHyPRP* genes in sorghum and hence an attempt was made to compare the exon–intron structures of all the identified genes. Out of 21 *SbPRP* genes, 4 have no introns, 2 exons were found in 4 genes, 3 exons in 3 genes, 4 in 3, 6 in 1, 7 in 1, 8 in 1, 10 in 1, and 12 in 3 (Table 1 and Figure 3A). The *SbPRP11*, *13*, and *18* have the highest number of exons, i.e., 12, while the least number of exons (one) was noticed in *SbPRP8*, *16*, *19*, and *20*. Of the 27 *SbHyPRPs*, 16 genes have been found without any introns, but comprise only one exon, 2 exons in 7 genes, 3 in 3, and 4 exons in one gene. The majority of the *SbHyPRPs* were intron less in comparison with *SbPRPs* (Table 2 and Figure 3B).

**TABLE 1:** List of identified PRPs exhibiting chromosomal location, sub group, length, DNA binding domains (DBD), molecular weight, (MW), iso-electric point (pI), GRAVY, no. of exons, localization, and instability and aliphatic indexes.

Gene	Common name	AA	Chr loc.	Pi/MW	DBD	Exon	Locali zation	GRAVY	Instability index	Aliphatic index	TMH MM
SORBI_3001G265900	SbPRP-1	624	1	9.37/71127.19	133–209	4	C	–1.066	59.37	42.88	0
SORBI_3001G266100	SbPRP-2	324	1	7.74/34937.58	34–108	2	E/V	–0.630	51.39	51.54	1
SORBI_3001G266200	SbPRP-3	210	1	6.93/22303.42	31–107	2	E	–0.305	47.66	64.24	0
SORBI_3001G266300	SbPRP-4	266	1	9.09/29111.79	35–117	2	E	–0.318	40.84	68.98	1
SORBI_3001G438400	SbPRP-5	256	1	7.49/27050.04	32–113	7	E	–0.455	44.55	61.48	0
SORBI_3001G438500	SbPRP-6	259	1	6.51/26803.38	34–116	2	E	–0.177	71.97	45.27	1
SORBI_3001G493300	SbPRP-7	195	1	7.52/19452.47	35–109	3	P	0.313	43.46	97.74	2
SORBI_3003G195000	SbPRP-8	312	3	11.11/32187.12	44–75	1	C	–0.054	54.54	73.91	1
SORBI_3003G315500	SbPRP-9	69	3	4.59/25020.34	11–60	3	C	–0.220	84.11	53.77	0
SORBI_3003G336300	SbPRP-10	2477	3	6.67/278911.49	142–276	4	N	–0.622	40.73	71.53	0
SORBI_3004G060600	SbPRP-11	511	4	9.31/57657.25	320–421	12	Cy	–0.094	36.47*	106.71	0
SORBI_3004G180900	SbPRP-12	519	4	5.05/58183.27	308–345	8	N	–1.061	50.76	54.66	0
SORBI_3005G191600	SbPRP-13	481	5	7.03/52432.65	261–335	12	N/P/ER	–0.441	43.8	70.58	0
SORBI_3005G197700	SbPRP-14	264	5	6.95/29229.79	57–98	6	V	–0.406	48.76	86.44	1
SORBI_3006G031100	SbPRP-15	309	6	10.99/33712.72	118–150	3	Cy/M/ER	–0.219	44.26	95.34	0
SORBI_3006G211701	SbPRP-16	148	6	8.68/15027.87	65–147	1	C	0.388	53.06	96.89	0
SORBI_3006G238000	SbPRP-17	380	6	6.54/41092.34	321–375	10	Cy	0.126	42.81	92.45	0
SORBI_3007G074200	SbPRP-18	1016	7	8.61/112336.86	977–1016	12	N	–0.476	40.98	72.33	0
SORBI_3008G082400	SbPRP-19	205	8	9.33/22334.14	13–118	1	V	–1.491	77.34	34.39	1
SORBI_3010G054600	SbPRP-20	324	10	9.43/34162.79	238–319	1	C	–0.347	87.66	68.83	0
SORBI_3010G278800	SbPRP-21	814	10	5.42/91773.76	537–582	4	C	–0.970	47.90	64.05	1

a.a, amino acids; Chrom, chromosome; C, chloroplast; Cy, cytoplasm; N, nucleus; P, plastid; V, vacuole; E, extra cellular; ER, endoplasmic reticulum; GRAVY, grand average hydropathy.

* Stable.

**TABLE 2:** List of identified HyPRPs exhibiting chromosomal location, length of aa, DNA binding domains (DBD), molecular weight (MW), iso-electric point (pI), GRAVY, no. of exons, localization, and instability and aliphatic indexes.

Gene	Common name	AA	Chr loc.	Pi/MW	DBD	Exon	Locali zation	GRAVY	Instability index	Aliphatic index	TMHMM
SORBI_3001G302600	SbHyPRP-1	287	1	7.52/27092.47	142–214	2	E	0.192	44.22	78.89	0
SORBI_3001G304201	SbHyPRP-2	218	1	8.90/22653.51	86–147	2	C	0.321	49.09	102.39	0
SORBI_3001G304300	SbHyPRP-3	129	1	6.00/13201.74	53–126	1	E	0.676	45.72	118.68	0
SORBI_3001G473200	SbHyPRP-4	252	1	8.88/26375.49	42–120	1	C	0.175	49.82	87.54	1
SORBI_3001G541300	SbHyPRP-5	150	1	8.00/14891.71	69–149	1	C	0.594	58.85	100.07	0
SORBI_3001G541400	SbHyPRP-6	175	1	8.82/15975.22	91–173	1	E	0.334	60.61	84.17	1
SORBI_3001G542000	SbHyPRP-7	137	1	8.03/13709.31	54–135	1	E	0.587	49.89	107.66	1
SORBI_3001G112200	SbHyPRP-8	415	1	9.66/45451.14	354–386	3	M	–0.147	50.26	88.63	0
SORBI_3003G126100	SbHyPRP-9	613	3	9.33/66940.26	209–286	2	C	–0.359	58.43	72.92	0
SORBI_3003G058900	SbHyPRP-10	198	3	6.58/21159.92	61–157	1	E	–0.257	35.37*	70.96	0
SORBI_3003G149800	SbHyPRP-11	636	3	9.00/70134.60	48–63	3	N	–0.575	51.65	74.47	0
SORBI_3003G329400	SbHyPRP-12	171	3	9.40/19721.55	81–119	2	N	–0.559	58.31	75.91	0
SORBI_3004G291500	SbHyPRP-13	131	4	8.05/12991.45	49–130	1	C	0.664	43.56	105.80	0
SORBI_3004G292001	SbHyPRP-14	122	4	7.45/12258.61	41–122	1	C	0.833	38.84*	116.72	0
SORBI_3004G292250	SbHyPRP-15	122	4	7.45/12255.61	41–122	1	C	0.831	37.27*	117.54	0
SORBI_3004G292900	SbHyPRP-16	621	4	9.13/69704.92	38–115	2	C	–0.303	44.73	81.24	1
SORBI_3005G046700	SbHyPRP-17	131	5	5.99/13456.17	65–129	1	E	0.722	48.17	122.82	0
SORBI_3006G172550	SbHyPRP-18	132	6	8.36/13331.89	50–131	1	E	0.597	35.81*	107.95	1
SORBI_3006G172601	SbHyPRP-19	328	6	8.75/36189.90	48–125	4	E/V	0.200	66.33	99.02	1
SORBI_3006G211701	SbHyPRP-20	148	6	8.68/15027.87	65–147	1	C	0.388	53.06	96.89	0
SORBI_3006G238100	SbHyPRP-21	380	6	6.54/41092.34	321–375	3	Cy	0.126	42.81	92.45	0
SORBI_3007G030100	SbHyPRP-22	148	7	9.66/15852.42	26–111	1	E	–0.299	68.58	78.58	0
SORBI_3008G158501	SbHyPRP-23	229	8	7.45/19735.64	146–228	1	E	0.181	36.93*	69.48	1
SORBI_3008G158600	SbHyPRP-24	273	8	7.45/22529.25	190–272	1	E	0.107	42.70	61.87	1
SORBI_3010G003600	SbHyPRP-25	249	10	8.71/26495.94	166–248	2	C	0.208	56.48	100.68	1
SORBI 3010G054800	SbHyPRP-26	324	10	9.43/34162.79	238–319	2	C	–0.347	87.66	68.83	0
SORBI_3010G204700	SbHyPRP-27	309	10	9.10/31575.13	135–192	1	E/V	–0.189	69.42	77.80	0

a.a, amino acids; Chrom, chromosome; C, chloroplast; Cy, cytoplasm; N, nucleus; M, mitochondria; V, vacuole; E, extra cellular; ER, endoplasmic reticulum; GRAVY, grand average hydropathy.

* Stable.

### *In silico* prediction of potential *cis*-regulatory elements

Promoter analysis predicted that *SbPRP* and *SbHyPRP* genes have potential *cis*-regulatory elements such as Dc3 Promoter Binding Factor (DPBF, participate in seed specific and/or ABA-inducible expression), MYC, MYB, etiolation, G-box, I-box, ANAERO, associated with abiotic stress, hormone-specific (ABRE, and ERE), T/GBOX for jasmonic acid, biotic stress-responsive WBox, development-specific (pollen, endosperm, and meristem specific) and guard cell-specific elements. Phosphorous (BIHD)-, ammonium-, sulfur-responsive (SURE), and iron (IRO2) starvation-responsive elements have also been predicted. They were rich with EECCRCAH1 elements, the binding site of MYB transcription factor; rbcS general consensus sequence; XYLAT, the core xylem gene set; Telo-box elements for activation of expression in root primordia (Supplementary Tables 2, 3).

### Phylogenetic and gene duplication analysis of SbPRP, SbHyPRPs, and gene synteny in sorghum, rice, and *Arabidopsis*

**FIGURE 4:**
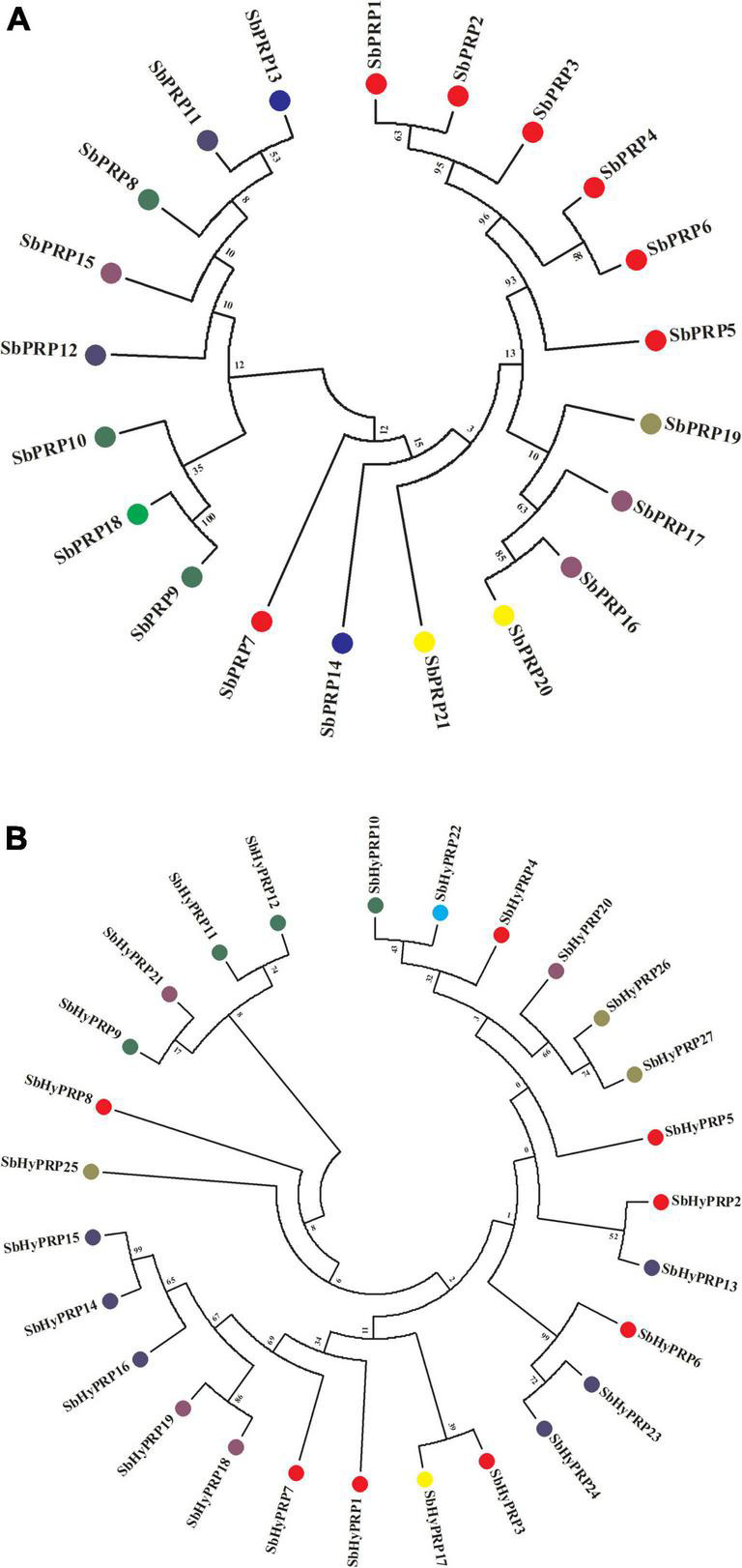
Phylogenetic analysis of SbPRP and SbHyPRP genes. **(A)** Phylogenetic tree of PRPs in *Sorghum.*Sub-groups are distinguished by different colors based on their chromosome locations, **(B)** phylogenetic tree of HyPRPs in *Sorghum*. Sub-groups are distinguished by different colors based on their chromosome locations.

**FIGURE 5:**
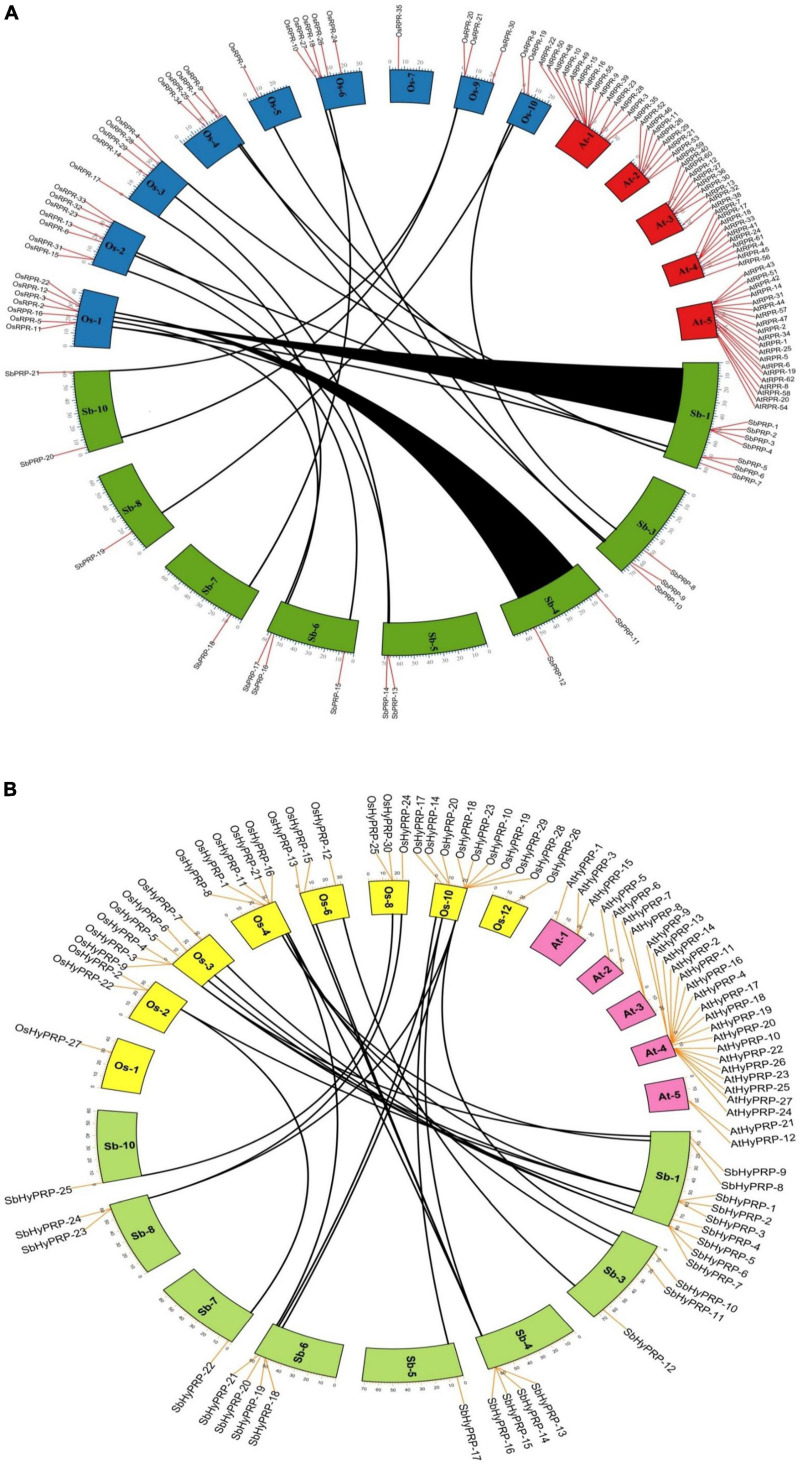
Synteny analysis of SbPRP and SbHyPRP genes. **(A)** Synteny analysis of PRP in *Sorghum bicolor, Oryza sativa*,and *Arabidopsis thaliana*. **(B)** Synteny analysis of HyPRP in *Sorghum bicolor, Oryza sativa*,and *Arabidopsis thaliana*. The map was built with TB tools software. Sb, *Sorghum bicolor*; Os, *Oryza sativa;* PRP, Proline-rich proteins; HyPRP, Hybrid proline-rich proteins.

The phylogenetic analysis of SbPRPs has been clustered into two clades, based on homology and conserved motifs. Clade I is subdivided into 7, whereas clade II is grouped into 4. A total of five paralogous pairs were observed in the SbPRP family, of which two tandem (SbPRP1/2 and SbPRP4/6 on chromosome 1) and the remaining segmental duplications (Figures 2A, 4A). A phylogenetic tree was constructed to see the evolutionary relationship of SbPRPs with *Oryza* and *Arabidopsis* ancestors for monocot and dicots. All of them exhibited lesser evolutionary relationships, but species-specific clusters were noticed. SbPRPs showed five orthologous relationships, of which four are with *Oryza* and only one with *Arabidopsis* (Sorbi_3001G266100/AT5G9170) (Supplementary Figure 1). The phylogenetic tree of SbHyPRPs clustered into two clades, based on their conserved motifs and subcellular localizations. Clade I is divided into two groups, whereas clade II is clustered into eight groups. A total of nine paralogs were predicted, of which four tandem (SbHyPRP11/12 on chromosome 3; SbHyPRP18/19 on 6; SbHyPRP23/24 on chromosome 9; SbHyPRP26/27 on chromosome 10) and five segmental duplications (Figures 2B, 4B). The SbHyPRPS have been predicted to have 10 orthologous relationships with *Oryza*, and only one with *Arabidopsis* (Sorbi_3010G204700/AT2G10940) (Supplementary Figure 2). Comprehensive phylogenetic trees were constructed by including *Oryza, Arabidopsis, Zea, Medicago, Lycopersicum, Solanum, Medicago, Hordeum, Beta vulgaris, and Setaria* for PRP and HyPRPs. They display species and family specificities. While SbPRPs showed 12 orthologous relationships with *Zea*, SbHyPRPS depicted 3 with *Zea* (Supplementary Figures 3, 4). Darwinian selection in paralogous and orthologous duplications was uncovered by calculating the non-synonymous substitution (d_*N*_) to synonymous (d_*S*_) ratios. Among the five paralogs of SbPRPs, only one event (SbPRP9/SbPRP18) showed the dN/dS ratio below 1, implying purifying selection, while the remaining four followed a positive/Darwinian selection (Supplementary Table 4). Out of the 5 PRP orthologous events, three events (SORBI_3006G211701/Os06G0104800; SORBI_3010G054600/Os06G0168700; SORBI_3001G266100/At5G59170) exhibited the d_*N*_/d_*S*_ ratio less than 1, while the remaining two showed Darwinian/positive selection (Supplementary Table 5). Of the nine paralogous events of SbHyPRPs, only two (SbHyPRP14/SbHyPRP15, SbHyPRP18/SbHyPRP19) exhibited purifying selection (Supplementary Table 6). Out of six orthologs of SbHyPRPs, only one event (SORBI_3001G304201/Os10G40420) followed purifying selection and the remaining positive selection (Supplementary Table 7). Syntenic genes for sorghum PRP and HyPRPs were mapped to *Oryza* and *Arabidopsis*. For *SbPRP* genes, chromosome 1 displays seven genes, followed by chromosomes 3 and 6 with three genes, chromosomes 4, 5, and 10 with two genes, and chromosomes 7 and 8 with one gene each, respectively (Figure 5A). For *SbHyPRP*, *S. bicolor* chromosome 1 displays nine genes, followed by chromosomes 4 and 6 with 4, chromosome 3 with 3, chromosome 8 with 2, and chromosomes 5, 7, 10, 11, and 12 with one gene each, respectively (Figure 5B).

### *In silico* characterization of SbPRP and SbHyPRP proteins

It has been predicted that *SbPRP* genes encoding peptides span from 69 (SbPRP9) to 2477 (SbPRP10) amino acids with the pI value varying from 4.59 (SbPRP9) to 11.11 (SbPRP8). The molecular weights range from 15027.87 (SbPRP-16) to 278911.49 (SbPRP10) Da. The pI values ranges between 5.99 (SbHyPRP17) and 9.66 (SbHyPRP8 and 22), and the molecular weights extend from 12255.61 (SbHyPRP15) to 70134.60 Da (SbHyPRP11). The analyses reveal that majority of them are basic in nature with few exceptions. All the predicted SbPRPs are unstable, (if instability index > 40, considered stable in nature) and the instability index ranges between 40.73 (SbPRP10) and 87.66 (SbPRP20). In the case of SbHyPRPs, 81.48% of proteins were unstable and the remaining were stable (Table 1). The instability index varies from 35.37 (SbHyPRP1) to –87.66 (SbHyPRP26) (Table 2). SbPRPs are hydrophilic proteins (86%) with lower aliphatic indices. When compared, the majority of the SbHyPRPs (66.66%) are found to be hydrophobic in nature, with a high aliphatic index. Out of 21 SbPRPs, only 38.09% contain transmembrane helices, while 33.33% of SbHyPRPs consist of the same (Tables 1, 2). Six (28.57%) of the SbPRPs target chloroplasts, four extracellular, one plastid, two the cytoplasm, three the nucleus, two the vacuole, and the remaining three target different localizations (Table 1). Out of 27 SbHyPRPs, 11 have been distributed in the chloroplast, 10 extracellular, one in mitochondria, two in the nucleus, one in the cytoplasm, and two extracellular or vacuolar (Table 2). Based on the analysis, it has been predicted that chloroplast and extracellular are the major target sites for their localization. Most of the SbPRPs have been predicted in the cytoplasm, indicating that they are secretary proteins. SbPRPs have been predicted to have a signal peptide at the N-terminus and a hydrophobic cysteine-rich region at the C-terminus. These results indicate that PRP proteins are secreted to the extracellular matrix through the signal peptide. The majority of the SbPRP proteins phosphorylate at serine and threonine sites and very few of them at tyrosine residues, threonine being the predominant one. Protein kinase C (PKC) and unspecified (unsp) are the most dominant types present in higher amounts in all the SbPRP proteins. Next to CKII, PKA P38MAPK, CDK5, PKG, and GSK3 are the most common kinases associated with phosphorylation (Supplementary Table 8). Whereas, in the case of SbHyPRPs, serine is the governing phosphorylation site followed by threonine and tyrosine. TPKC, unsp, PKA, CKII, cdc2, P38MAPK, CDK5, PKG, and GSK3 are the common kinases for SbHyPRP protein phosphorylations (Supplementary Table 9).

### Conserved motif analysis

The MEME web server was used to analyze the motif distribution of SbPRPs and SbHyPRPs. In SbPRPs, the predicted motif 1 appears as the biggest and motif 10, the smallest. The SbPRP family does not exhibit a common motif distribution pattern. Motif 1 is the common motif found at N-terminus in fewer proteins. The motifs 2, 3, 4, and 5 are the structural conserved motifs, present in the middle of the proteins (Supplementary Figures 5, 6). In SbHyPRPs, motif 6 is the largest with 43 amino acids in length, and motifs 7 and 8 with 11 amino acids, are the smallest. Motif 4 is the common conserved motif present at N-terminus, while motif 3 is at C-terminus. Motif 8 exhibited repeated distribution in SbHyPRP 11, 26, and 27 (Supplementary Figures 7, 8). These conserved motifs reflect features for PRP and HyPRP proteins as shown by Wang et al. (2006) and hence provide confirmatory identification of proteins from the sorghum genome.

### Homology modeling and validation of proline-rich proteins and hybrid proline-rich proteins

Protein models help to understand structure-function relationships (Rasheed et al., 2020). So, the 3D structures of SbPRP and SbHyPRP proteins were predicted with the best PDB templates (Supplementary Figure 9). The template PDB id, template description, chain, model of the oligomer, and their structure validations are represented in Supplementary Table 10. 3D structures of one SbPRP (SbPRP12) and six SbHyPRP (SbHyPRP2, SbHyPRP5, SbHyPRP6, SbHyPRP13, SbHYPRP17, and SbHYPRP26) proteins displayed significant sequence similarity percent ranging from 31.1% (SbPRP12) to 55.26% (SbHyPRP6). 3D structures of five SbPRP proteins (SbPRP3, SbPRP6, SbPRP10, SbPRP17, and SbPRP19) did not show any significant (<30%) sequence similarity (Supplementary Table 10). All the generated Ramachandran plots for structure validation are represented in Supplementary Figure 10.

### miRNA target site prediction and analysis of acetylation and methylation sites

Plant regulatory small RNAs (sRNAs) include microRNAs (miRNAs) and small interfering RNAs (siRNAs) like phasiRNAs which regulate gene expressions, thus, modulating plant growth as well as stress tolerance (Haak et al., 2017). The psRNA Target Server predicts the miRNAs and their target sites in *SbPRP* and *SbHyPRP* genes. The 21 SbPRPS predicted in the present study appear to be the targets for 34 different miRNAs. The sbi-miR437 and sbi-miR5568 have been predicted to target the SbPRP family. The miRNAs participate in cleavage and translation inhibition events. The SbPRP13, 18, and 20 appear as the major targets for different miRNAs (Supplementary Table 11). Interestingly, 29 different miRNAs target 25 *SbHyPRPs* and moderate the activity. The *SbHyPRP2*, *3*, *14*, *16*, *18*, and *24* are the major gene targets for the majority of the miRNAs (Supplementary Table 12). SbPRP9 and SbPRP21 showed a high number of acetylations (Supplementary Table 13). When compared with SbPRPs, SbHyPRPs displayed a diminished number of acetylation and methylation sites. SbHyPRP 11 and 16 have been predicted to have myriad acetylation, and methylation sites (Supplementary Table 14). Prediction of miRNAs, methylation and acetylation sites indicate that *SbPRP* and *SbHyPRP* genes might be regulated epigenetically.

### Prediction of simple sequence repeats

Out of a total of 36 SSRs, 18 were predicted in PRPs and HyPRPS. They were present on chromosomes 1, 3, 4, 5, 6, 7, and 10. Chromosome 1 has been found as the hot spot with three PRPs and five HyPRPs. While the majority of them carried single SSRs, PRP8, 10, HyPRP1, 4, and 6, have 2, and PRP6 and 8 have 3 SSRs. Tri-nucleotide repeats have been predicted as the major repeats (25), followed by hexamer (7) and tetra-nucleotide repeats (4) (Supplementary Table 15).

### Digital expressions of *SbPRP* and *SbHyPRP* genes in diverse tissues, stages, and stress conditions

**FIGURE 6:**
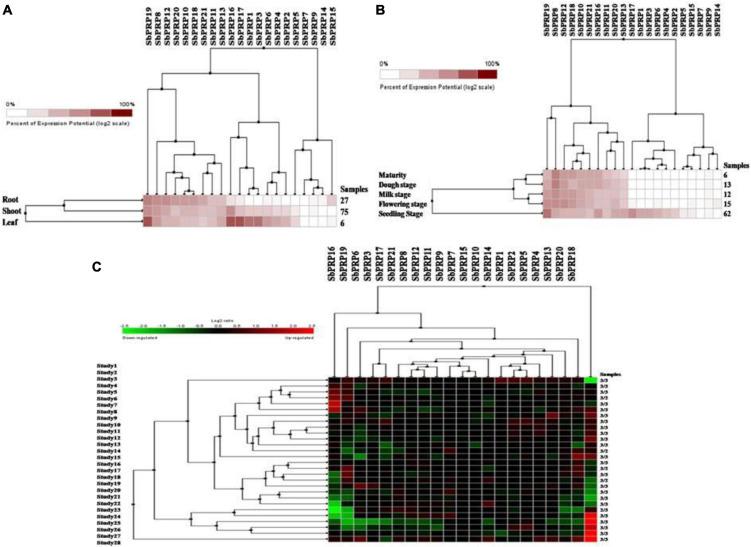
Digital expression profile of *SbPRP* genes in different organs, developmental stages under cold and drought stresses. **(A)** *SbPRP* in anatomical tissues, **(B)** *SbPRP* genes in developmental stages, **(C)** *SbPRP* gene expressions under cold and drought stress conditions.

**FIGURE 7:**
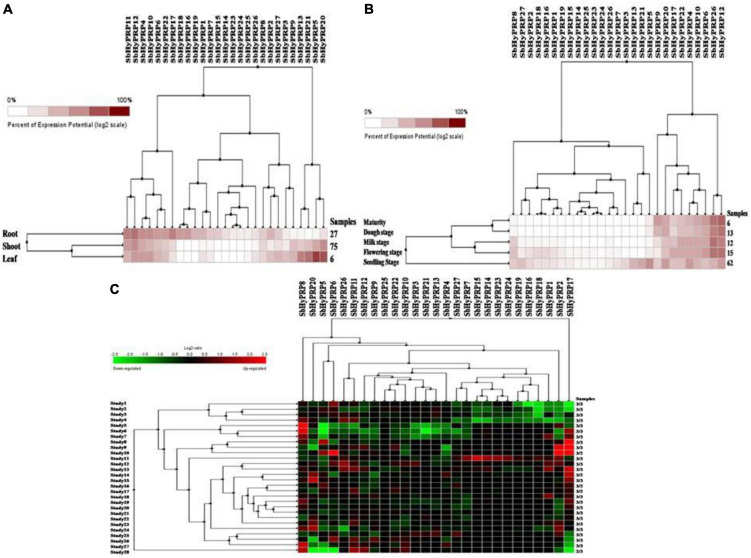
Digital expression profile of *SbHyPRP* genes in different organs, developmental stages under cold and drought stresses. **(A)** *SbHyPRP*in anatomical tissues, **(B)** *SbHyPRP* in developmental stages, **(C)** *SbHyPRP* gene expressions under cold and drought stresses.

Using digital data, the expression of 21 *SbPRP* genes was analyzed in the root, shoot, and leaf tissues exposed to cold and drought stress conditions. Overall, high expression levels of *SbPRPs* were noticed in the leaf compared to root and shoot tissues. Out of 21 *SbPRPs*, *SbPRP16*, *SbPRP17*, *SbPRP19*, *SbPRP1*, and *SbPRP3* displayed high expressions in shoot tissues (Figure 6A). Among diverse developmental stages such as maturity, dough, milking, flowering, and seedling, the seedling stage showed superior expressions followed by the flowering stage. *SbPRP19*, *SbPRP16*, *SbPRP17*, *SbPRP1*, *SbPRP13*, *SbPRP13*, and *SbPRP20* showed high expression levels in seedlings treated with cold and drought stresses in comparison with other developmental stages (Figure 6B). SbPRP18 displayed higher expression levels, followed by 16, and 19 under both the stresses (Figure 6C). *SbHyPRP5*, *SbHyPRP20*, *SbHyPRP13*, and *SbHyPRP21* showed elevated expressions in the leaf. Expressions of *SbHyPRP12*, *SbHyPRP17*, and *SbHyPRP11* were strikingly high in roots (Figure 7A). *SbHyPRP12* and *SbHyPRP26* were markedly upregulated during maturity, dough, milking, and flowering stages in comparison with other genes. The majority of the *SbHyPRP* genes are also expressed during the seedling stage (Figure 7B). The heat map of *SbHyPRPs* showed both up- and down-regulation up to 2.5-folds under cold and drought stresses (Figure 7C). *SbHyPRP17*, *SbHyPRP2*, *SbHyPRP18*, and *SbHyPRP20* showed upregulation in comparison with other genes under stress conditions. *SbHyPRP2*, *SbHyPRP8*, and *SbHyPRP17* displayed high expressions under drought compared to cold stress conditions (Figure 7C).

### Expression analysis of *SbPRP* genes in different tissues, varying stress conditions, and Zn treatment

**FIGURE 8:**
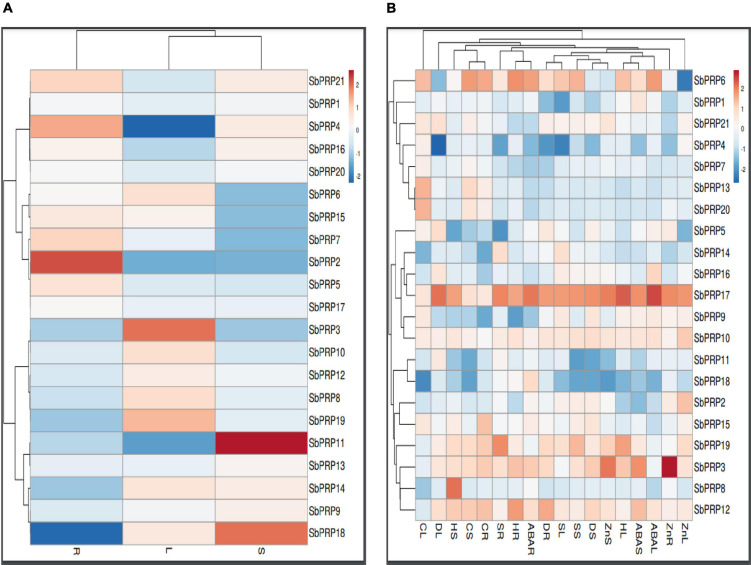
qRT-PCR expression profiling of *SbPRP*s. **(A)** Native expression analysis in root, stem, and leaf, **(B)** relative expression analysis in root, stem, and leaf under drought, salt, heat, cold, ABA, and Zn stresses. R, root; S, stem; L, leaf.

Expression analysis of *SbPRP* and *SbHyPRP* genes were carried out in root, stem, and leaf to address their role in diverse organs. There is no single gene that has exhibited uniform levels of expression in all the three tissues. Under control conditions (without any stress), only *SbPRP2* (1.303-folds) and *SbPRP4* (0.824-folds) were constitutively expressed in roots, while *SbPRP11* (1.820-folds) and *SbPRP18* (1.473-folds) in the stem, and *SbPRP3* (0.985) in leaf tissues (Figure 8A and Supplementary Table 16). Drought has considerably stimulated the expression of *SbPRP17* in leaf (4.73-folds), stem (4.08-folds), and root (3.94-folds), but only in the roots of *SbPRP12* (3.61-folds). Salt stress induced the activity of *SbPRP17* by 4.25-, 3.53-, and 4.44-folds in leaf, stem, and root tissues, respectively, and also only in the roots of *SbPRP19* (4.34-folds). Heat stress has upregulated *SbPRP3* (3.31-folds), *SbPRP6* (3.34-folds) and *SbPRP17* (4.94-folds), and PRP19 (4.00-folds) in the leaf, *SbPRP8* (4.58-folds) and *SbPRP17* (3.744-folds) in the stem, *SbPRP6*, *SbPRP12* and *SbPRP17* by 4.05-, 3.84-, and 3.65-folds respectively in the root (Figure 8B and Supplementary Table 16). Cold stress has stimulated *SbPRP4*, *SbPRP5*, *SbPRP6*, *SbPRP7*, *SbPRP9*, *SbPRP10*, *SbPRP13*, *SbPRP15*, *SbPRP17*, *SbPRP20*, and *SbPRP21* in the leaf between 3.08 to 4.99-folds. The fold-wise increase in the stem of *SbPRP3*, *SbPRP6*, *SbPRP12*, *SbPRP13*, *SbPRP17*, and *SbPRP19* ranged from 3.01 to 5.20-folds. In the case of roots, the elevated transcript levels were 4.53, 5.38, 4.82, 3.03, 4.82, 3.24, and 4.59-folds in *SbPRP3*, *SbPRP6*, *SbPRP12*, *SbPRP13*, *SbPRP15*, *SbPRP17*, and *SbPRP19*, respectively. While ABA stimulated *SbPRP6* and *SbPRP17* by 3.85-, 3.65- (*SbPRP6*), 4.94-, and 4.20-folds (*SbPRP17*) in the leaf and root, it activated *SbPRP3*, *SbPRP12*, and *SbPRP17* in the stems by 4.00, 3.04, and 4.00-folds, respectively (Figure 8B and Supplementary Table 16). Zn has jacked up only *SbPRP17* by 3.58-folds in the leaf, *SbPRP3* and *SbPRP17* in the stems by 3.51- and 3.37-, and in the roots by 4.81- and 3.52-folds respectively. From the results obtained, it is inferred that *SbPRP17* is critical for stress tolerance since it is expressed under various abiotic stress conditions in all three tissues, followed by *SbPRP3*, *SbPRP6*, and *SbPRP12* (Figure 8B, Supplementary Figure 11, and Supplementary Table 16).

### Transcript profiling of *SbHyPRP* genes in different tissues, diverse abiotic stresses, and Zn treatment

**FIGURE 9:**
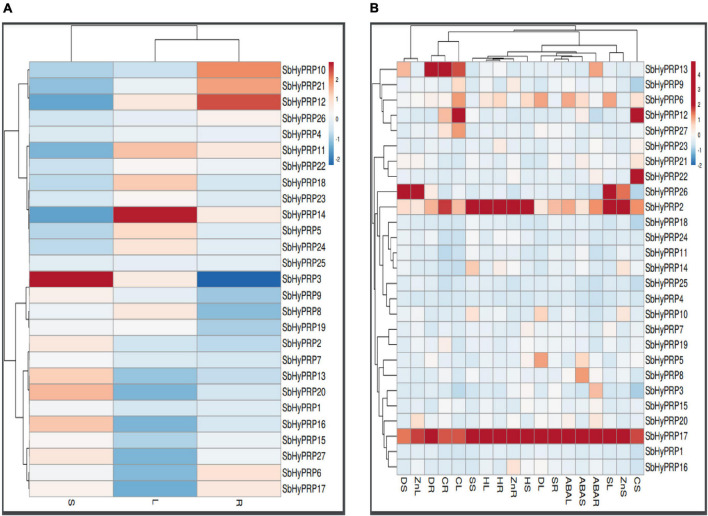
qRT-PCR expression profiling of *SbHyPRP*s. **(A)** Native expression analysis in root, stem, and leaf, **(B)** relative expression analysis in root, stem, and leaf under drought, salt, heat, cold, ABA, and Zn stresses. R, root; S, stem; L, leaf.

Unlike *SbPRPs*, *SbHyPRPs* displayed uniform constitutive expressions in all the tissues. In roots, *SbHyPRP12* was enhanced by 1.75-, *SbHyPRP10* by 1.60-, and *SbHyPRP21* by 1.54-folds when compared to the expression levels of the housekeeping gene actin. In the case of the stem, *SbHyPRP3* was upregulated by 2.77-, *SbHyPRP20* by 2.03-, *SbHyPRP16* by 1.95, and *SbHyPRP13* by 1.91-folds. While in leaf tissues, gene expressions increased by 1.77- in *SbHyPRP14*, 1.35- in *SbHyPRP11*, and 1.32-folds in *SbHyPRP18* devoid of any stress (Figure 9A and Supplementary Table 17). Significant upregulation of the *SbHyPRP2* and *SbHyPRP17* genes was noticed in comparison with control tissues regardless of the tissue and type of stress imposed. Drought stress has increased the expressions of *SbHyPRP2* by 5.16-, 9.67-, and 8.17-folds in the leaf, stem, and root, respectively. *SbHyPRP17* registered an upregulation of 26.66-, 17.01-, and 15.35-folds in leaf, stem, and root, respectively, under drought. The highest expression of 44.15-folds was recorded in *SbHyPRP20* in the stem tissues under water-deficit conditions. Expressions varied between 3.45-, and 24.00-folds in *SbHyPRP5*, *SbHyPRP6*, *SbHyPRP10*, *SbHyPRP13*, *SbHyPRP16*, *SbHyPRP27* genes under drought stress (Figure 9B and Supplementary Table 17). Under salt stress, the highest expression of 21.79-folds was noticed in the root tissues of *SbHyPRP17*, followed by leaf tissues (19.10-folds). *SbHyPRP2* was upregulated by 15.60-folds in the leaf under saline conditions. Activities were also high under salt stress in *SbHyPRP6*, *SbHyPRP7*, *SbHyPRP10*, *SbHyPRP14*, and *SbHyPRP26*. *SbHyPRP17* displayed a dramatic increase in the activity (30.82-folds) under heat stress in the leaves, followed by *SbHyPRP2* in the leaf (23.44-folds). *SbHyPRP2* and *SbHyPRP17* also recorded markedly high activities in the stem and root tissues (15.3-, 12.58-, 8.54-, 13.40-folds respectively). Cold temperature induced the transcript levels of *SbHyPRP2*, *SbHyPRP6*, *SbHyPRP9*, *SbHyPRP12*, *SbHyPRP17*, *SbHyPRP26*, and *SbHyPRP27* in the leaves in the range of 3.34- (*SbHyPRP26*) to 19.96-folds (*SbHyPRP12*). Again in the stem, the expression level was high in *SbHyPRP12* (11.35-folds). *SbHyPRP2* and *SbHyPRP13* exhibited 10.13- and 17.83-folds enhanced transcript levels in the roots under cold stress (Figure 9B and Supplementary Table 17). Significant upregulation in the activity of *SbHyPRP17* was noticed in the leaf, stem, and root tissues (30.87-, 16.10-, and 18.46-folds), and the activities ranged between 3.13- and 9.57-folds in other genes when exposed to the phytohormone ABA. Zn treatment boosted the activities of *SbHyPRP26* (25.39-folds) and *SbHyPRP17* (11.96-folds) in the leaves. While *SbHyPRP2* displayed the maximum activity (16.83-folds) in the stem, the roots of *SbHyPRP17* showed 11.47-fold higher activities compared to the controls (Figure 9B, Supplementary Figure 12, and Supplementary Table 17).

## Discussion

### Characterization of *SbPRP* and *SbHyPRP* gene homologs

The accessibility of the whole sorghum genome sequence (Paterson et al., 2009) has provided an excellent opportunity for genome annotation, comparative genomic analysis, and understanding of the structural and functional variations among the diverse gene families. Publicly available genome sequence data have helped us in identifying different gene families in sorghum (Kumari et al., 2018; Maheshwari et al., 2019; Nagaraju et al., 2020, 2021). PRPs and HyPRPs play crucial roles in abiotic stress and growth functions as well (Zhang and Schläppi, 2007; Priyanka et al., 2010; Huang et al., 2011; Berendsen et al., 2012; Li et al., 2016; Efthimiadou et al., 2020). Recently, *OsHyPRP06/R3L1* has been found to modulate root system development besides salt tolerance via apoplastic ROS homeostasis in rice (Zhao et al., 2021). Great variability exists in the number of *PRPs* and *HyPRPs* in different taxa (Dvorakova et al., 2007; Neto et al., 2013; Fujino et al., 2014; Showalter et al., 2016; Kapoor et al., 2019; Zhang et al., 2021), which could be due to differences in the genera or the method employed. Genome-wide analyses of sorghum resulted in the identification of 21 *SbPRPs* and 27 *SbHyPRPs* in the present study. Detection of both *SbPRPs* and *SbHyPRPs* in the present study in *S. bicolor* indicates their conservation in evolution.

### Prediction of exons and introns

The exon-intron structure in the genomic sequences furnishes an insight into evolutionary relationships among the genes or organisms and perhaps needs intensified investigations (Koralewski and Krutovsky, 2011). Our predicted results on intron-exon structure organization both in *SbPRPs* and *SbHyPRPs* conform to the results obtained from other species (Fujino et al., 2014; Kapoor et al., 2019; Zhang et al., 2021). Several *SbPRP* and *SbHyPRP* genes have been found with no or few introns. It has been shown in yeast, *A. thaliana*, and mice that genes with rapidly changing expression levels in response to environmental stresses contain lower intron densities (Jeffares et al., 2008). Introns delay the regulatory responses, hence genes that need rapid adjustment for survival against the changing environment are intron poor (Jeffares et al., 2008; Lan et al., 2013). It appears, therefore, that there is an intron loss during the evolution of a few eukaryotic lineages. Besides structural diversity, the complexity of the eukaryotic genes might contribute to the evolution of gene families. Our results show that the *SbPRP* and *SbHyPRP* genes have been distributed randomly on different chromosomes.

### Predicted *cis*-regulatory elements

In the present study, *cis*-elements such as DPBF, MYC, MYB, G-box, ABRE, and ERE have been identified in sorghum besides phosphorous, ammonium, sulfur, and iron starvation-responsive elements. SbPRPs have been found rich in phosphorous, iron, ammonium, and sulfur-responsive elements, while SbHyPRPs in sulfur-responsive elements, indicating the diverse roles they play in nutrient homeostasis. These elements might target transcription factors like SPB, zinc finger, WRKY, WD-40, NAC, MYB, HSFs, GRAS, ARFs, and bHLH families and play a vital role in abiotic stress tolerance (Katiyar et al., 2015). MYB and ABRE elements might play a role in the ABA-dependent signaling pathway in response to drought, salt, and cold stresses. Promoter analysis also revealed the presence of elements that participate in sugar signaling, nuclear factor binding, and biotic stress-responsive *cis*-elements such as WBOXNTERF3, WBOXATNPR1, and CGTCA that respond to wounds, pathogens, and salicylic acid (Hwang et al., 1998; Schumann et al., 2017). In cotton, the *GhHyPRP4* promoter is cold-inducible (Huang et al., 2011). Kapoor et al. (2019) reported a good correlation between inducible expression in *OsHyPRP16* in response to the pathogen *Magnaporthe oryzae*, hormones ABA, methyl jasmonate, salicylic acid, and abiotic stresses like cold, and salt in the promoter sequences of rice.

### Phylogenetic analysis

Phylogenetic trees infer that PRPs are highly conserved in different taxa. Analysis of the phylogenetic trees reveals that homologous gene pairs have high sequence identities with other taxa compared, thus displaying their evolutionary relationships. The clustering of the PRP and HyPRPs of sorghum, rice, maize, and disconnection from Arabidopsis indicates their evolutionary divergence during monocotyledon lineage-specific evolution. Expansion of gene families occurs through segmental, and tandem duplications besides transposition events (Kong et al., 2007). In the present study, a total of eight paralogs were observed including two regional and six segmental duplication events in *SbPRPs*, while three regional and five segmental duplications in *SbHyPRPs*, indicating that segmental and tandem duplications are responsible for gene family expansion. Gene order conservation of PRP and HyPRP has been identified by circos maps. High conservation of *PRP* and *HyPRP* genes has been noticed between *Sorghum* and *Oryza* when compared to *Sorghum* and *Arabidopsis*, indicating that rice and sorghum are closely related.

### Subcellular localization of protein predictions

Predictions related to the subcellular localization of the proteins in the extracellular matrix, identification of signal peptides, and also cysteine-rich regions indicate that all these proteins might not be secretory type as has also been pointed out by Banday et al. (2022). PRPs and HyPRPs are located on the cell wall and plasma membrane and regulate the synthesis of cell walls aside from responding to abiotic stresses (Jose-Estanyol et al., 2004; Kavi Kishor et al., 2015; Mellacheruvu et al., 2016; Gujjar et al., 2018; Li et al., 2019; Zhang et al., 2021). In citrus, CsPRP4 protein was localized in the plasma membrane (Ma et al., 2013), and in *Juglans sigillata*, JsPRP1 in the epidermal cells (Liu et al., 2018). While HyPRP protein from *Arabidopsis* has been identified on the cell wall (Zhang and Schläppi, 2007), GhHyPRP3 from cotton has been noticed on the plasma membrane (Qin et al., 2013). In apple, PRP proteins have been detected in the cell membrane, nucleus, and chloroplast (Zhang et al., 2021). AZELAIC ACID INDUCED 1 (AZI1) is a HyPRP, a member of the HyPRP superfamily (Dvorakova et al., 2007), and a signal-anchored protein that has a proline-rich region (PRR) for targeting the protein to plastids (Cecchini et al., 2015, 2021). HyPRPs possess transmembrane domains besides a PRR and a lipid transfer domain. A feedback regulatory loop exists between glycerol-3-phosphate (G3P) and lipid transfer protein Defective in Induced Resistance (DIR1) in plants and AZI1 mediates azelaic-acid-induced systemic immunity (Yu et al., 2013). But, the subcellular locations of HyPRPs and their precise functions during development and biotic and abiotic stress tolerance have mostly not yet been identified. HyPRPs may be localized to the plastid outer membranes, thylakoid membranes, membranes of the endoplasmic reticulum, plasma membrane, or plasmodesmata (Banday et al., 2022). Few of the HyPRPs have a set of proteins that target the outer membrane of the plastid with the aid of PRRs. HyPRPs modulate systemic immunity against *Pseudomonas syringae* and also help in the colonization of *P. simiae* WCS417 in the roots. Taken together, it appears that HyPRPs may facilitate signal molecule transport and help in plant immunity, growth, and development besides abiotic stress tolerance. Similar to the studies carried out by He et al. (2002); Fujino et al. (2014), and Kapoor et al. (2019), our findings suggest that SbPRP and SbHyPRP proteins are localized in different subcellular locations.

### Homology modeling

The highly expressed SbPRP and SbHyPRP protein structures were predicted and structurally validated for accuracy using Ramachandran plots. Similar studies were reported previously by Kumar et al. (2018). Understanding the prediction of protein structures can facilitate the understanding of their function (Jumper et al., 2021).

### Methylation sites, identification of micro RNAs

*SbPRPs* and *SbHyPRPs* showed methylation as well as acetylation sites. Elements of epigenetic regulation such as histone variants, chromatin remodeling, non-coding RNAs, and DNA methylations are highly conserved among plant systems and represent an important component of the omnifarious biological process (Goldberg et al., 2007; Allis and Jenuwein, 2016; Ueda and Seki, 2020). It is known that epigenetic regulation including acetylation and methylation coordinates abiotic stress responses (Kim et al., 2015; Asensi-Fabado et al., 2017; Luo et al., 2017; Ueda and Seki, 2020). Methylations and acetylations found in sorghum might regulate the genes post-transcriptionally which are implicated in abiotic stresses.

Micro RNAs (miRNAs) are small, non-coding, regulatory elements, which play overriding roles in gene regulation by disturbing the transcripts of genes and mediating the plant adaptation to the changing environmental conditions (Bartel, 2004). In the present study, miRNAs that target SbPRPs and SbHyPRPs have been predicted. We assume that these miRNAs regulate these genes. miRNAs are critical not only for stress responses but also for plant development (Sunkar et al., 2007; Rubio-Somoza et al., 2009). In the present investigation, miR437 and miR5568 have been identified as dominant miRNAs, specific for monocots and absent from eudicots (Sunkar et al., 2007). The conservation and divergence of miRNAs may help to understand miRNA evolution and identify the species-specific miRNAs and the roles that they play in gene regulation. SICKLE (*SIC*), a proline-rich protein has been found vital in *A. thaliana* for development and abiotic stress tolerance. It is also involved in microRNA biogenesis (Zhan et al., 2012). Loss-of-function *sic-1* mutant *Arabidopsis* displayed higher levels of introns, sickle-like margin, delayed flowering, hypersensitivity to chilling, and salt stresses. The *SIC* protein has been observed to colocalize with the miRNA biogenesis component hyponastic leaves 1 (HYL1). These observations have led Zhan et al. (2012) to conclude that *SIC* is necessary for the biogenesis of some miRNAs and spliced introns. DNA and chromatin modifications modulate the expression of stress-responsive genes. Such modifications are also heritable inferring their role in the evolutionary mechanisms associated with abiotic stress tolerance (Singroha et al., 2022).

### Predicted simple sequence repeats

Simple sequence repeats are used generally to study genetic relatedness or variation among the species. SSRs get mutated quickly; hence, differentiation of closely related species is possible. So far, several QTLs associated with diverse traits were identified in many crops. The predicted SSRs associated with PRPs and HyPRPs could be informative and used to evaluate the genetic diversity and relationships among diverse sorghum lines. Such markers may also be useful to the breeders who are aiming to generate abiotic stress tolerant sorghum lines.

### Digital expressions of *SbPRP* and *SbHyPRP* genes at diverse organs and developmental stages and transcript profiling of *SbPRP* and *SbHyPRP* genes under abiotic stresses, abscisic acid, and zinc

Digital expression data indicated that the expressions of *PRP* and *HyPRP* genes were higher in leaf tissues during the seedling stage of plant development. The results indicate the importance of *SbPRP19*, *SbPRP16*, *SbPRP17*, *SbPRP1*, *SbPRP13*, *SbPRP13*, and *SbPRP20* genes during seedling when compared to other stages. Such genes need to be functionally validated to find out the seedling specific roles they play especially under water-deficit conditions. Increased expressions of both *SbHyPRP12* and *SbHyPRP26* during maturity, dough, milking, and flowering stages infer the crucial role they might play during these stages. High expressions of *SbHyPRP2*, *SbHyPRP8*, and *SbHyPRP17* under water-deficit conditions compared to cold stress surmise that functional validation of these genes needs to be carried out.

Insolubilization of PRPs leads to the formation of protein–protein or protein–carbohydrate linkages within cell walls and assists in the stability of extracellular matrix in plants (Bernhardt and Tierney, 2000; Akiyama and Pillai, 2003). PRPs respond to the changing environments and thus abet in coping with stress situations (Kavi Kishor et al., 2015; Zhang et al., 2021). Literature on the transcript expressions of PRPs is scanty. Munoz et al. (1998) in *Cicer arietinum* and Xu et al. (2007) in *Gossypium hirsutum* have shown tissue-specific expression of PRP genes. *GmPRP1* mRNAs have been found in elongating and mature regions of the seedling hypocotyl cells in *Glycine max*, *GmPRP2* in phloem cells, and *GmPRP3* in the endodermoid layer of cells in the hypocotyl elongating region (Wyatt et al., 1992). In the case of *Arabidopsis*, *AtPRP1* and *AtPRP3* gene expressions were noticed in roots, and *AtPRP2* and *AtPRP4* in aerial organs (Fowler et al., 1999; Bernhardt and Tierney, 2000). *SbPRP* genes were activated under different abiotic stress conditions aside from ABA and Zn treatments in a tissue-specific manner in sorghum. This reveals that these genes are spatially regulated during plant development in a specific tissue or cell type as has been shown by others (Munoz et al., 1998; Xu et al., 2007; Zhang et al., 2021). Out of 21 *SbPRPs*, *SbPRP17* appeared to have ubiquitous expressions in all tissues though the magnitude differed, suggesting that *SbPRPs* function in many physiological processes during stress. It is known that *PRP* genes are up- and down-regulated, involved in plant growth and environmental challenges. In support of this statement, Gothandam et al. (2010) overexpressed rice *OsPRP3* and showed that transgenics acquire cold tolerance, but knockdown mutants display defects in flower morphogenesis. In *Arabidopsis*, *PRP* is critical for development, and abiotic stress tolerance is associated with microRNA biogenesis (Zhan et al., 2012). When *Gossypium hirsutum GhPRP5* gene was overexpressed in *Arabidopsis*, transgenics exhibited diminished growth in comparison with wild-type plants. On the other hand, *GhPRP5* knock-down mutants in cotton improved fiber development with longer fiber length (Xu et al., 2013). Thus, PRPs have been proved to be associated with positive or negative regulation of plant growth. In the present study, it has been noticed that *SbPRPs* respond to drought, salt, heat, cold, ABA, and Zn inferring that *SbPRPs* play critical roles, but the responses differ among various environmental stimuli and Zn treatment. Such a phenomenon was noticed in *Brassica napus*, and *BnPRP* responds to low and high temperatures, and drought, but not injury (Goodwin et al., 1996). In *Glycine max*, *GmPRP* was triggered by salicylic acid, an endogenous circadian rhythm and diverse abiotic stress conditions (He et al., 2002). Contrary to the above, *OsPRP* was down-regulated during submergence in *Oryza sativa* (Akiyama and Pillai, 2003). In the case of Arabidopsis, out of 18 *PRP* genes, nematode infection upregulated the expression of *PRP4*, *PRP11*, and *PRP16* and *P. syringae* infection induced *PRP9* and *PRP10* (Showalter et al., 2010). Overexpression of *Juglans sigillata JsPRP1* resulted in drought tolerance, CdCl_2_ stress, *Colletotrichum gloeosporiodes* infection (Liu et al., 2018). In apple also, many *MdPRP* genes were expressed in all tissues except *MdPRP4*, *MdPRP5*, *MdPRP7*, and *MdPRP8* (Zhang et al., 2021). Interestingly, Zn also stimulated *SbPRP3* and *SbPRP17* genes in all three tissues, but their implication in zinc nutrition or starvation await functional assays to demonstrate the precise role of ABA and Zn in *PRP* functioning. Overall, these findings indicate that *PRPs* are differentially regulated under diverse abiotic stress conditions and might play decisive roles in modulating growth in addition to stress responses.

HyPRPs are a large family of cell wall protein with a variable N-terminal domain and a conserved C-terminal domain which is related to non-specific lipid transfer proteins. Initially, they have been regarded as only cell wall proteins, but slowly their multifunctional nature is being unraveled. *HyPRPs* take an active part in abscission, cell elongation, cell wall synthesis, growth, development, and biotic and abiotic stress tolerance (Wu et al., 1993; Zhang and Schläppi, 2007; Dvorakova et al., 2012; Sundaresan et al., 2018). Compared to *SbPRP* genes, multiple *SbHyPRP* genes have been found upregulated upon exposure to different abiotic stress conditions. Several *HyPRP* genes have been cloned and overexpressed and the transgenics displayed freezing or low-temperature stress tolerance inferring their positive regulatory role in stress response (Zhang and Schläppi, 2007; Huang et al., 2011; Peng et al., 2015). Overexpression of other *HyPRP* genes such as *CcHyPRP* (*Cajanus cajan*), *GhHyPRP3* (*Gossypium hirsutum*), and *MfHyPRP* (*Medicago falcata*) increased the levels of tolerance to multiple abiotic stresses like salt, and cold temperature (Priyanka et al., 2010; Qin et al., 2013; Tan et al., 2013). Pitzschke et al. (2014) identified and characterized AZI1, a lipid transfer protein (LTP)-related HyPRP, a target of mitogen-activated protein kinase 3 (MPK3). While null mutants of *azi1* are salt susceptible, overexpressing lines exhibit tolerance. Thus, MPK3 appears as a positive regulator of AZI1 and a key interactant. *HyPRP* from *Arabidopsis* has been found to play a key role in SAR when infected with *Pseudomonas syringae* (Jung et al., 2009). Neto et al. (2013) identified soybean HyPRP family members and their responses to Asian soybean rust disease. *EARLI1* is an Arabidopsis *HyPRP*, protects plants against cold stress, and improves germination under low temperatures and salt stress conditions (Zhang and Schläppi, 2007). Experiments performed by Li et al. (2012) using recombinant EARLI1 protein indicate that its application to *Botrytis cinerea*, *Fusarium oxysporum* inhibits the growth and cell viability. These results point out that *HyPRPs* are positively associated with biotic stress tolerance. Conversely, Yeom et al. (2012) identified the *HyPRP1* gene in *Capsicum annuum* and *Nicotiana benthamiana* which is constitutively expressed in different organs. But the gene was down-regulated when inoculated with pathogens. Overexpression of the gene resulted in accelerated death along with the down-regulation of ROS-scavenging genes. Gene silencing suppressed pathogen-induced cell death but increased disease resistance. It appears therefore that *HyPRP* acts as a positive regulator of cell death and a negative regulator of basal defense against pathogens. A DOUBLE HYBRID PROLINE-RICH PROTEIN 1 (*AtDHyPRP1*) detected in *Arabidopsis* contains two tandem proline-rich domain-eight cysteine motifs (PRD-8CMs) and is a novel *HyPRP* and induced by salicylic acid, methyl jasmonate, virulent and non-virulent strains of the pathogen *P. synringae.* Overexpression of this gene resulted in increased pathogen resistance, but RNAi lines of *AtDHyPRP1* exhibited susceptibility, indicating its involvement in defense response (Li et al., 2014). Li et al. (2016) noticed that the *SpHyPRP1* gene isolated from *Solanum pennellii* play a negative role in abiotic stress tolerance in tomato. Similarly, *GhHyPRP1* negatively regulates the resistance to *Verticillium dahliae* in cotton via the thickening of cell walls and ROS accumulation (Yang et al., 2018). These studies underpin the significant role that *HyPRPs* play during biotic and abiotic stress tolerance.

## Conclusion

Proline-rich proteins and HyPRPs are dominant constituents of cell wall proteins and help in the stability of the extracellular matrix. Their role in plant growth, development, and biotic and abiotic stresses are being rolled in recent times. The present study predicts the identification of 21 PRPs and 27 HyPRPs in sorghum and also the presence of *cis*-regulating elements implicated in abiotic stress tolerance, and nutritional starvation. Further, qRT-PCR analysis indicates that *SbPRP* and *SbHyPRP* genes are stimulated under cold, drought, high temperature, and salt stress conditions in sorghum. However, it is necessary to understand the molecular regulation of PRP and HyPRP proteins and also the functional validation of these genes in a wide array of biotic and abiotic stress conditions.

## Data availability statement

The original contributions presented in this study are included in the article/Supplementary material, further inquiries can be directed to the corresponding author.

## Author contributions

PK and NS designed the experiments. MN, RV, and GR carried out the bioinformatics analysis. GR performed the qRT-PCR experiments. PK, NS, GR, MN, RV, NJ, AKS, PS, and SP prepared the manuscript and refined it. PK, MN, GR, NS, and AKS revised the manuscript. All authors have read and approved the final version of the manuscript.

## Footnotes

1
https://www.fao.org/faostat/en#data

2
http://www.plantgdb.org/

3
http://www.gramene.org/

4
http://genes.mit.edu/GENSCAN.html

5
http://www.genome.jp/tools/motif/

6
http://gsds.cbi.pku.edu.cn

7
http://www.bork.embl.de/pal2nal/

8
http://web.expasy.org/protparam

9
http://wolfpsort.org/

10
https://saves.mbi.ucla.edu/

11
http://bdmpail.biocuckoo.org/

12
http://bioinfo.icgeb.res.in/PRmePRed/

13
http://misaweb.ipk-gatersleben.de/

14
https://genevestigator.com/
