# Supplementary material for: Identification and analysis of proline-rich proteins and hybrid proline-rich proteins super family genes from Sorghum bicolor and their expression patterns to abiotic stress and zinc stimuli
Source: Front Plant Sci. 2022 Sep 26;13:952732. doi: 10.3389/fpls.2022.952732 (PMC9549341; doi:10.3389/fpls.2022.952732)
Supplement: Supplementary file 1 [file Presentation_1.pptx]

## Slide 1
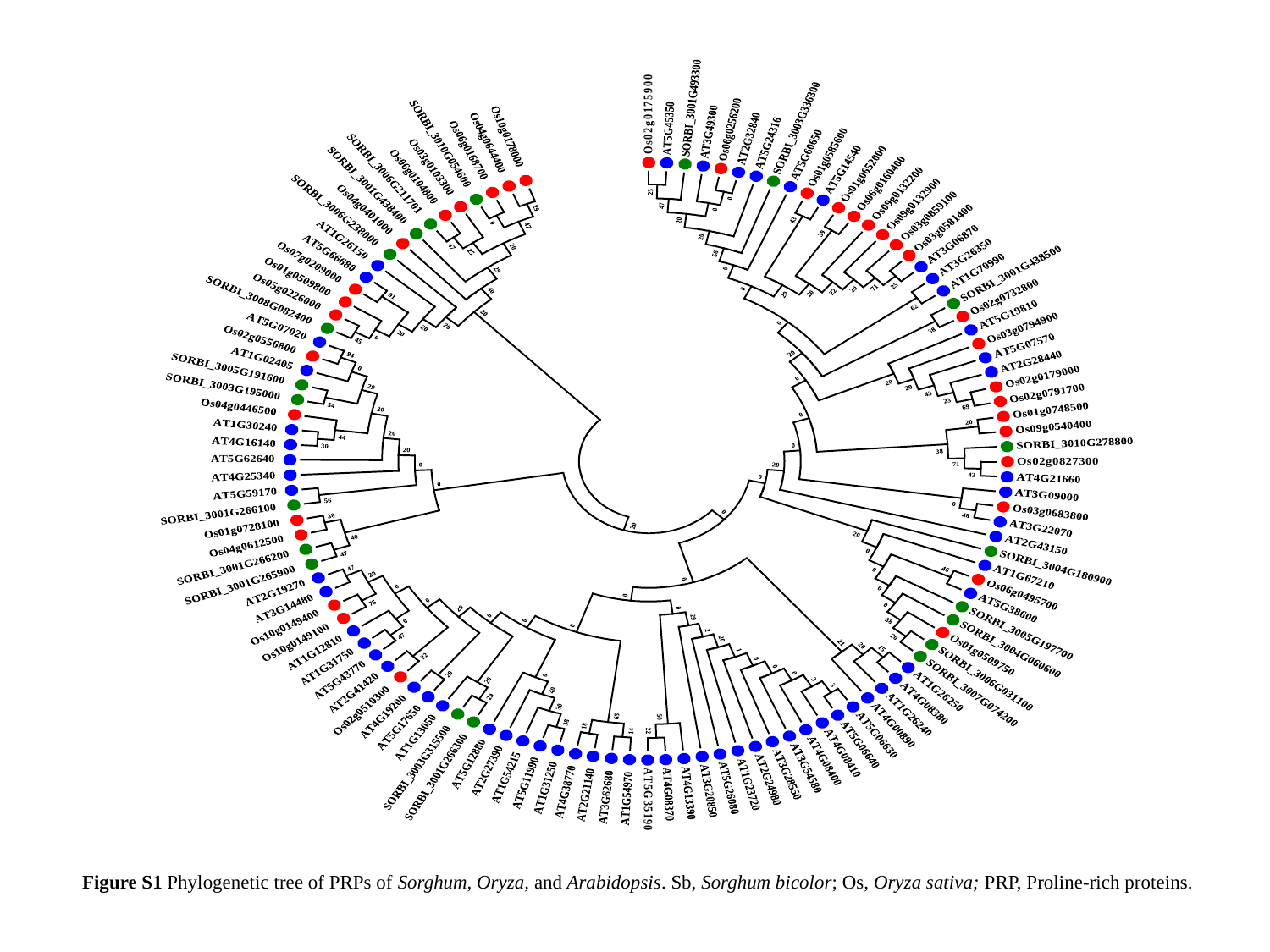

Figure S1 Phylogenetic tree of PRPs of Sorghum, Oryza, and Arabidopsis. Sb, Sorghum bicolor; Os, Oryza sativa; PRP, Proline-rich proteins.
