# Supplementary material for: Identification and analysis of proline-rich proteins and hybrid proline-rich proteins super family genes from Sorghum bicolor and their expression patterns to abiotic stress and zinc stimuli
Source: Front Plant Sci. 2022 Sep 26;13:952732. doi: 10.3389/fpls.2022.952732 (PMC9549341; doi:10.3389/fpls.2022.952732)
Supplement: Supplementary file 4 [file Presentation_4.pptx]

## Slide 1
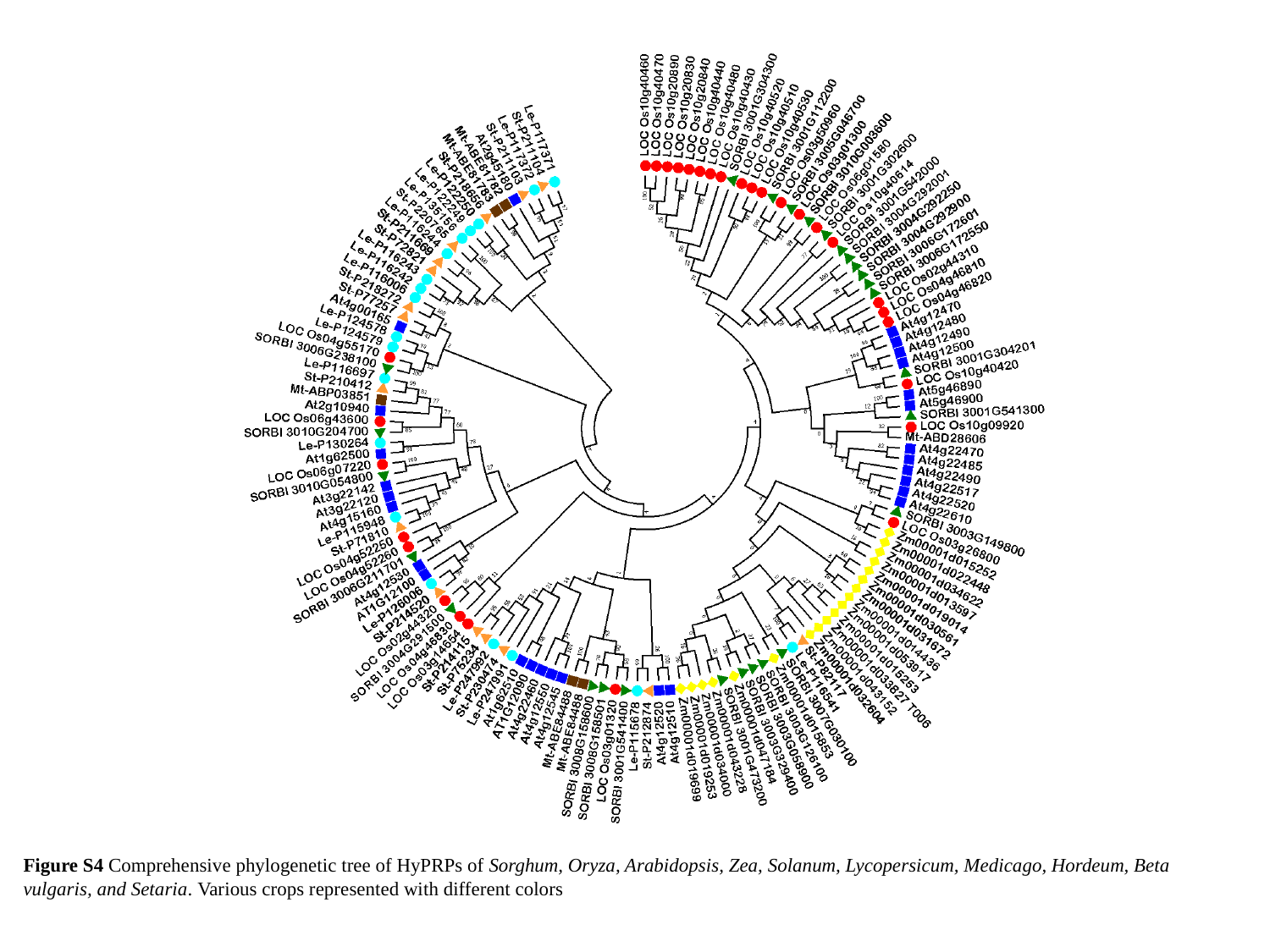

Figure S4 Comprehensive phylogenetic tree of HyPRPs of Sorghum, Oryza, Arabidopsis, Zea, Solanum, Lycopersicum, Medicago, Hordeum, Beta vulgaris, and Setaria. Various crops represented with different colors
