# Supplementary material for: Identification and analysis of proline-rich proteins and hybrid proline-rich proteins super family genes from Sorghum bicolor and their expression patterns to abiotic stress and zinc stimuli
Source: Front Plant Sci. 2022 Sep 26;13:952732. doi: 10.3389/fpls.2022.952732 (PMC9549341; doi:10.3389/fpls.2022.952732)
Supplement: Supplementary file 5 [file Presentation_5.pptx]

## Slide 1
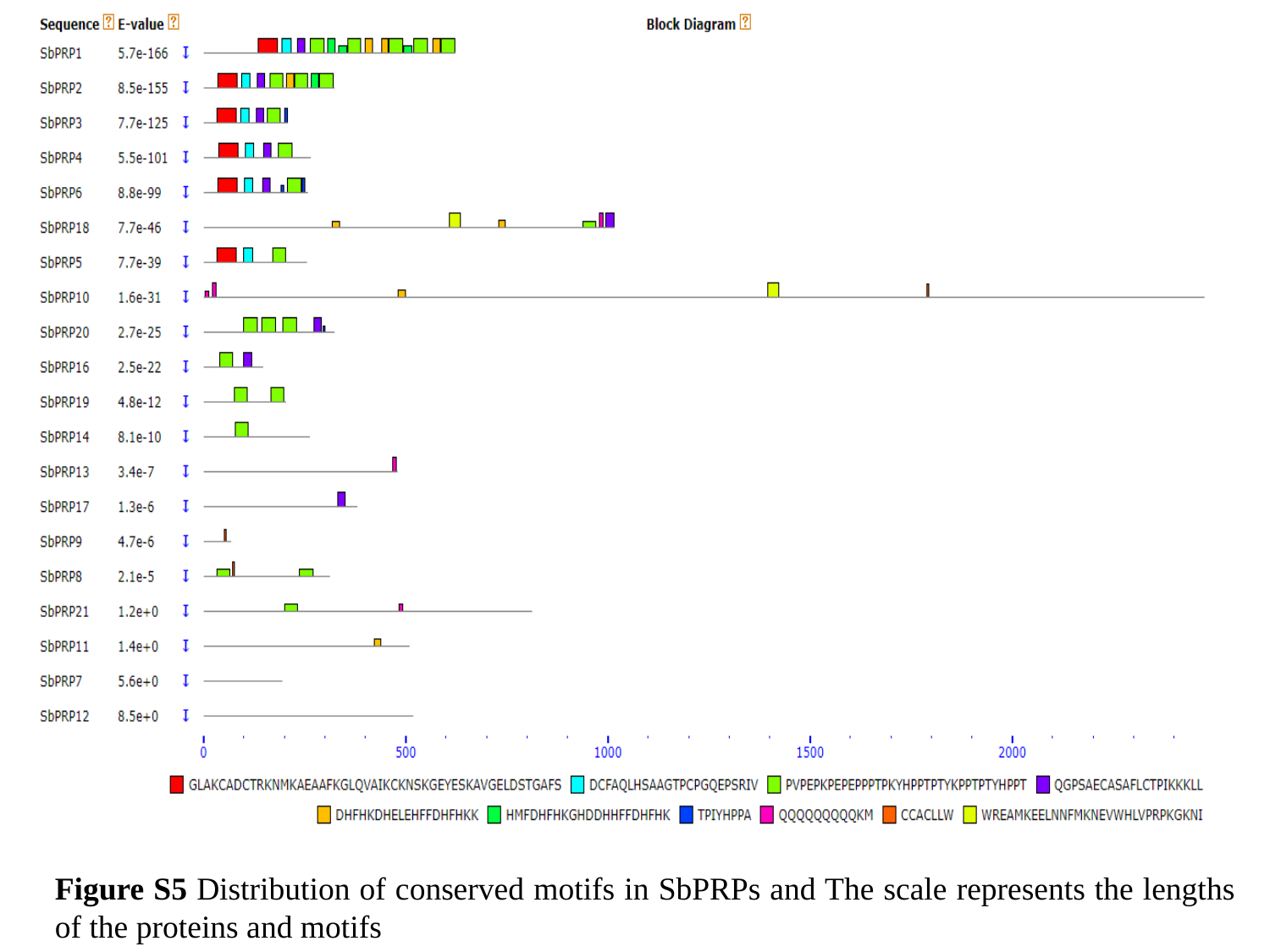

Figure S5 Distribution of conserved motifs in SbPRPs and The scale represents the lengths of the proteins and motifs
