# Supplementary figures and images for: Identification and analysis of proline-rich proteins and hybrid proline-rich proteins super family genes from Sorghum bicolor and their expression patterns to abiotic stress and zinc stimuli
Source: Front Plant Sci. 2022 Sep 26;13:952732. doi: 10.3389/fpls.2022.952732 (PMC9549341; doi:10.3389/fpls.2022.952732)

## Slide 1
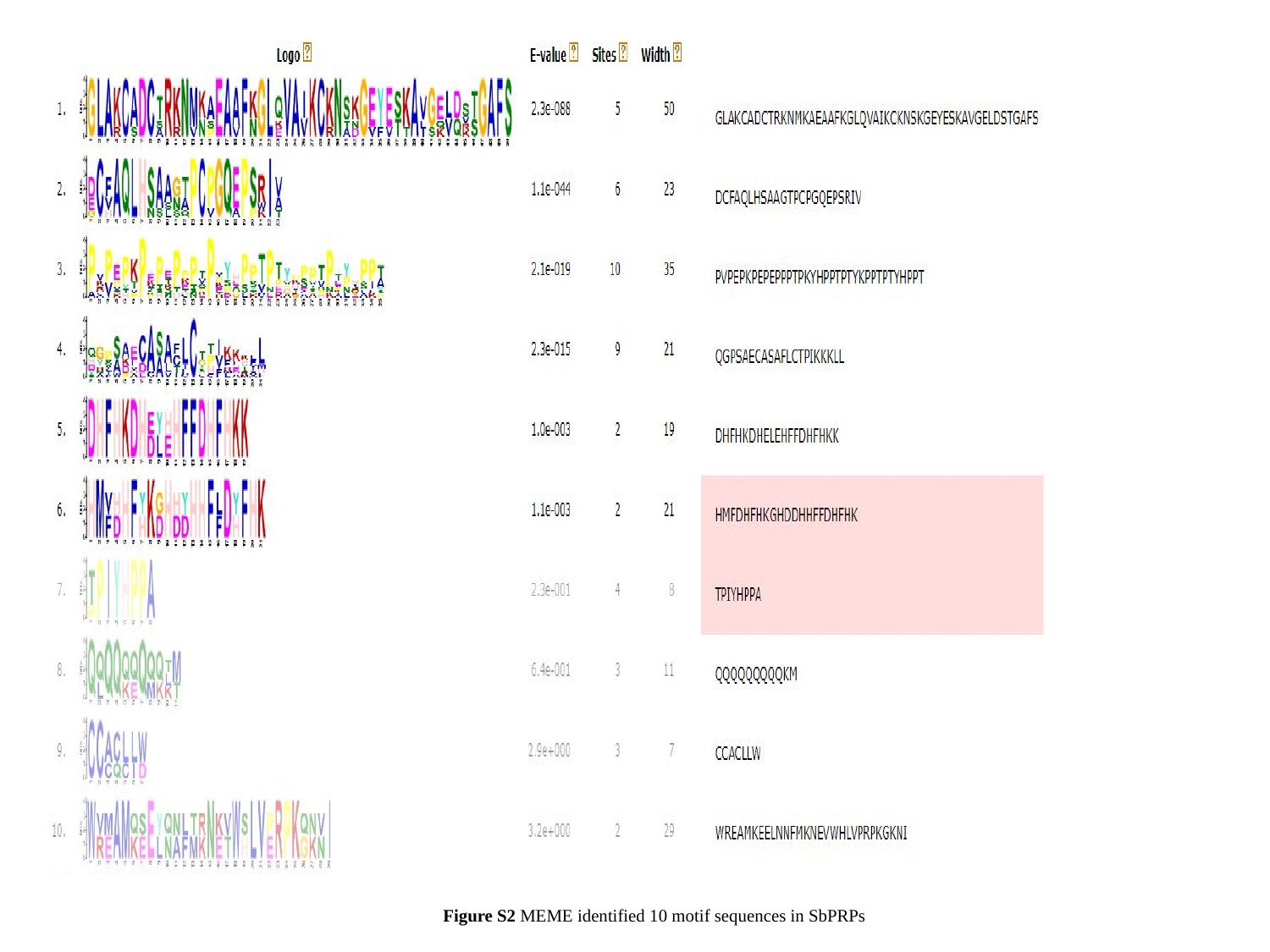

Figure S2 MEME identified 10 motif sequences in SbPRPs

Supplement: Supplementary file 6 [file Presentation_6.pptx]

## Slide 1
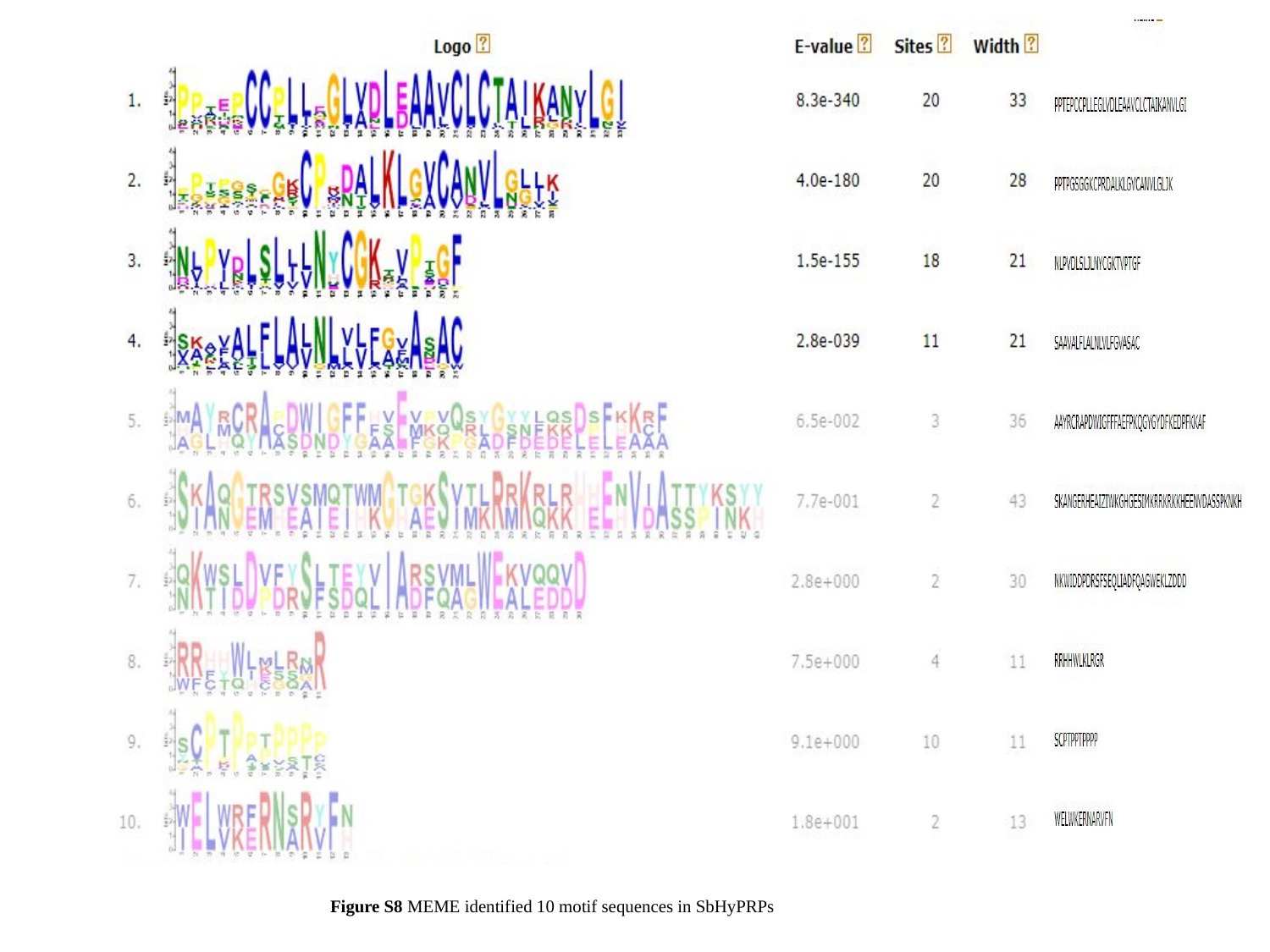

Figure S8 MEME identified 10 motif sequences in SbHyPRPs

Supplement: Supplementary file 8 [file Presentation_8.pptx]

## Slide 1
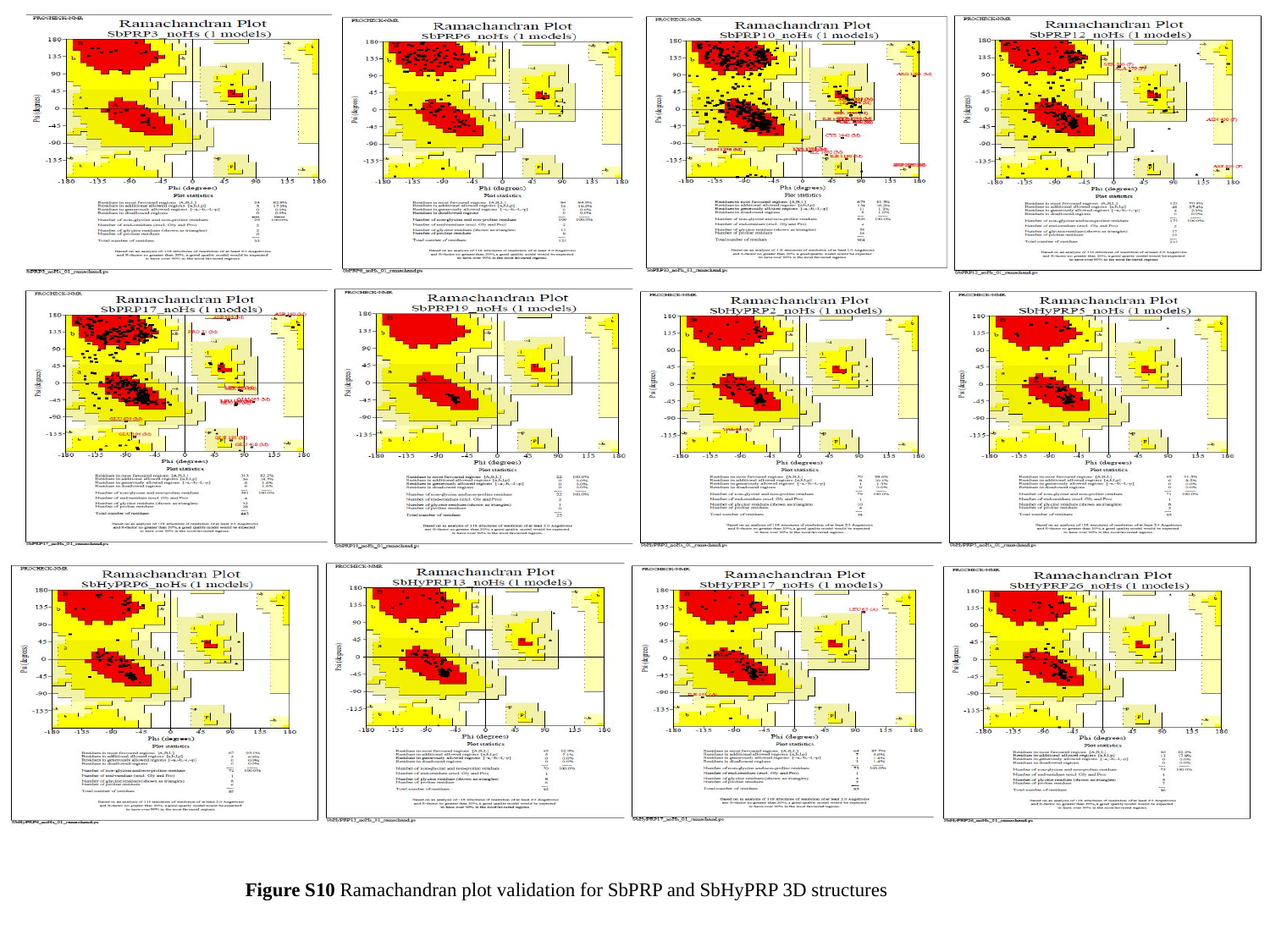

Figure S10 Ramachandran plot validation for SbPRP and SbHyPRP 3D structures

Supplement: Supplementary file 10 [file Presentation_10.pptx]
