# Supplementary material for: Identification and analysis of proline-rich proteins and hybrid proline-rich proteins super family genes from Sorghum bicolor and their expression patterns to abiotic stress and zinc stimuli
Source: Front Plant Sci. 2022 Sep 26;13:952732. doi: 10.3389/fpls.2022.952732 (PMC9549341; doi:10.3389/fpls.2022.952732)
Supplement: Supplementary file 9 [file Presentation_9.pptx]

## Slide 1
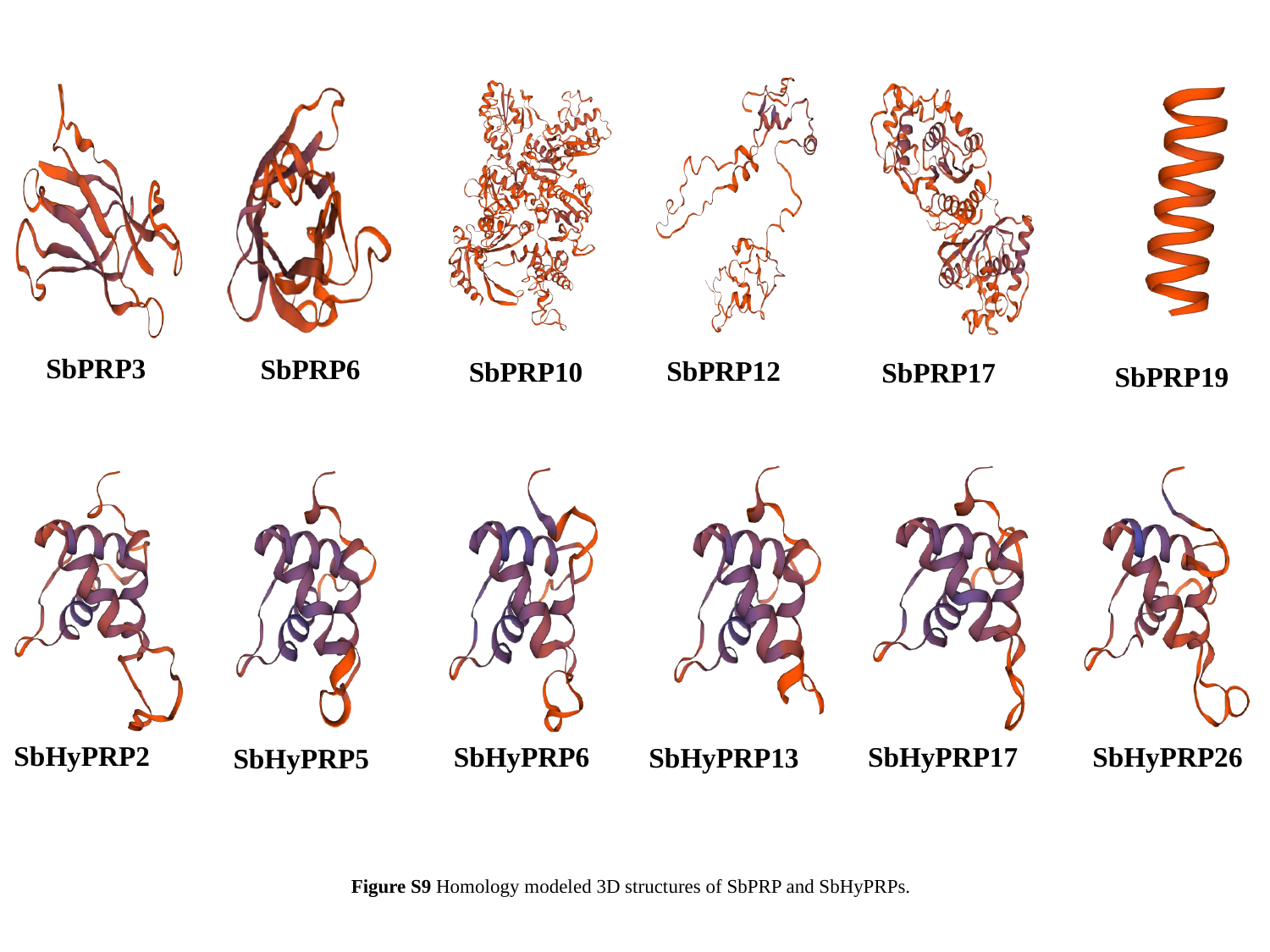

SbPRP6
SbPRP3
SbPRP10
SbPRP12
SbPRP17
SbPRP19
SbHyPRP13
SbHyPRP26
SbHyPRP2
SbHyPRP17
SbHyPRP6
SbHyPRP5
Figure S9 Homology modeled 3D structures of SbPRP and SbHyPRPs.
