# Supplementary material for: Identification and analysis of proline-rich proteins and hybrid proline-rich proteins super family genes from Sorghum bicolor and their expression patterns to abiotic stress and zinc stimuli
Source: Front Plant Sci. 2022 Sep 26;13:952732. doi: 10.3389/fpls.2022.952732 (PMC9549341; doi:10.3389/fpls.2022.952732)
Supplement: Supplementary file 11 [file Presentation_11.pptx]

## Slide 1
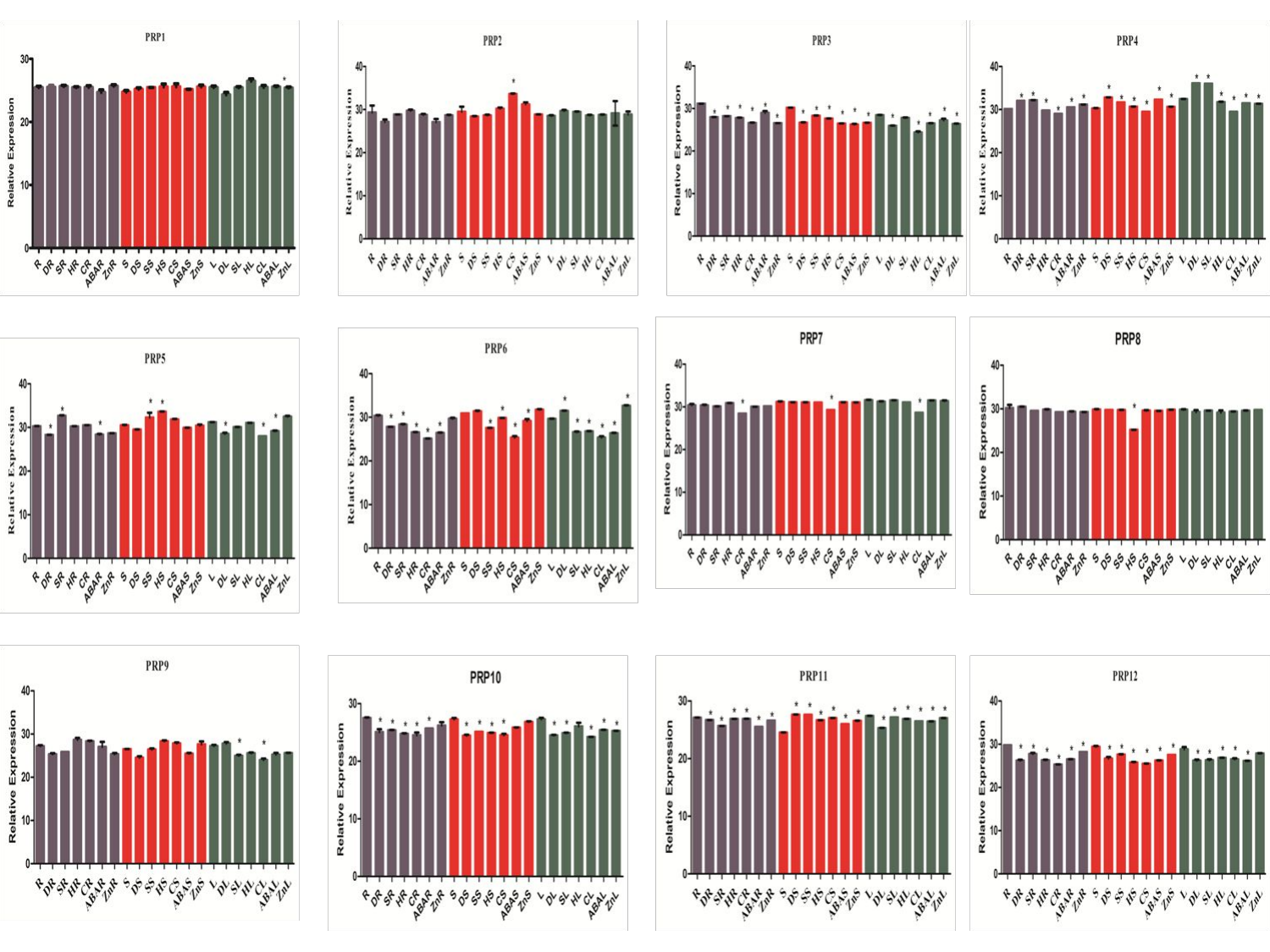

## Slide 2
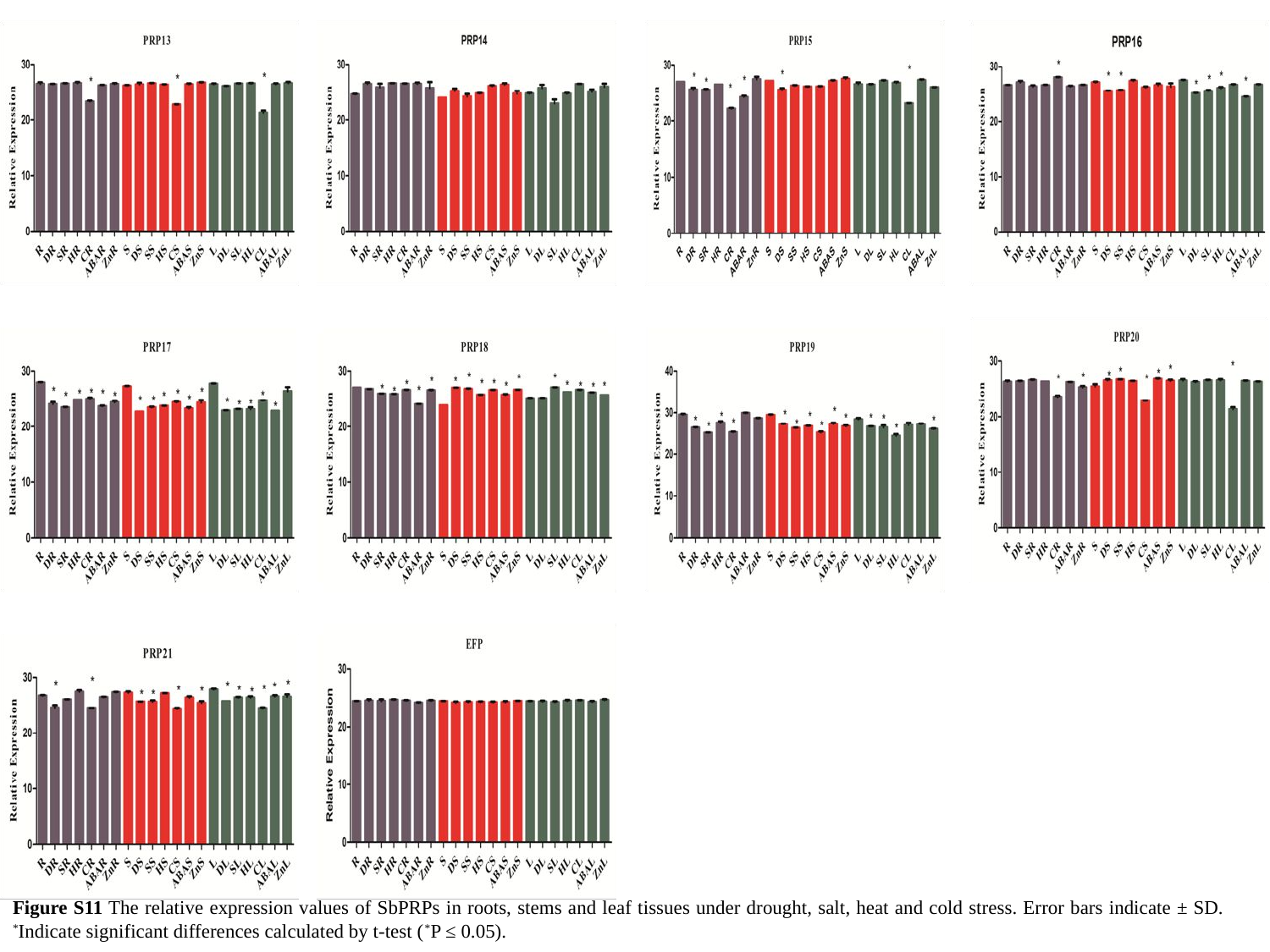

Figure S11 The relative expression values of SbPRPs in roots, stems and leaf tissues under drought, salt, heat and cold stress. Error bars indicate ± SD. *Indicate significant differences calculated by t-test (*P ≤ 0.05).
