# Supplementary material for: Identification and analysis of proline-rich proteins and hybrid proline-rich proteins super family genes from Sorghum bicolor and their expression patterns to abiotic stress and zinc stimuli
Source: Front Plant Sci. 2022 Sep 26;13:952732. doi: 10.3389/fpls.2022.952732 (PMC9549341; doi:10.3389/fpls.2022.952732)
Supplement: Supplementary file 12 [file Presentation_12.pptx]

## Slide 1
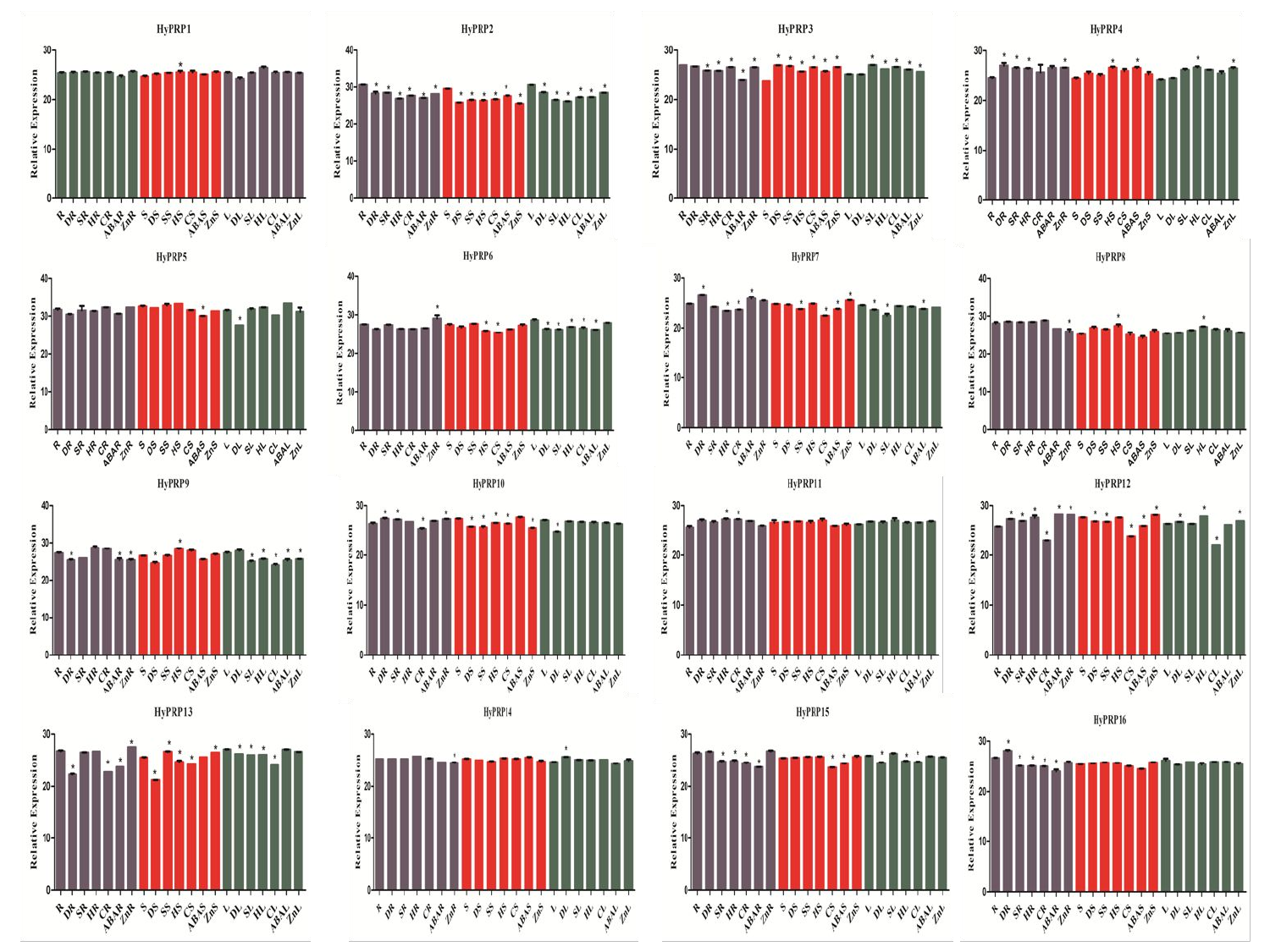

## Slide 2
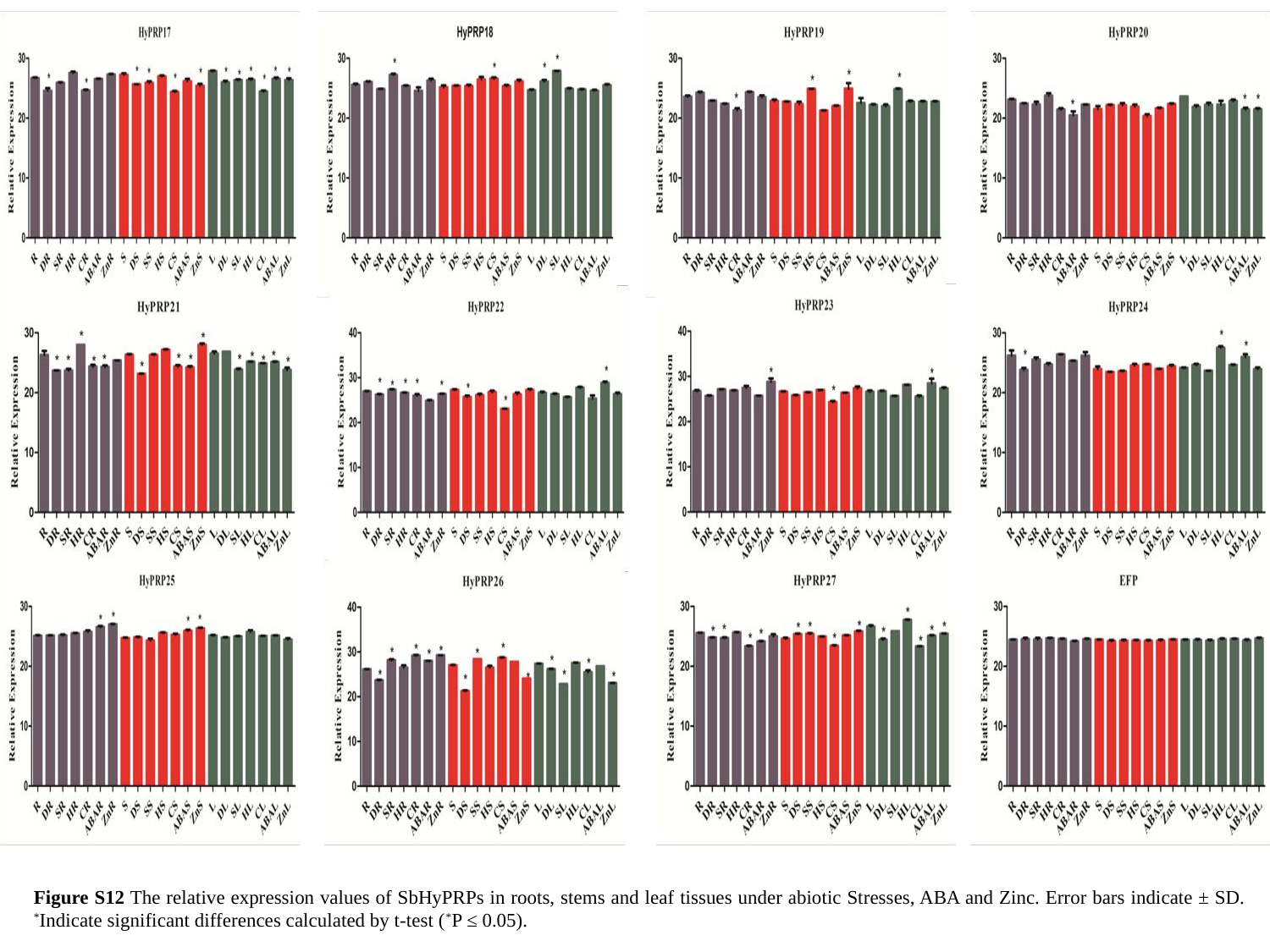

Figure S12 The relative expression values of SbHyPRPs in roots, stems and leaf tissues under abiotic Stresses, ABA and Zinc. Error bars indicate ± SD. *Indicate significant differences calculated by t-test (*P ≤ 0.05).
