# Supplementary material for: Identification and analysis of proline-rich proteins and hybrid proline-rich proteins super family genes from Sorghum bicolor and their expression patterns to abiotic stress and zinc stimuli
Source: Front Plant Sci. 2022 Sep 26;13:952732. doi: 10.3389/fpls.2022.952732 (PMC9549341; doi:10.3389/fpls.2022.952732)
Supplement: Supplementary file 13 [file Table_1.doc]

**Table S1.** *SbPRP* and *SbHyPRP* gene specific primers for gene **expression analysis by** qRT-PCR

| Gene ID | primer sequence | Size | Tm | % GC | Amplicon size |
| --- | --- | --- | --- | --- | --- |
| SbPRP-1F | GCATGCAAATCCAAAGTGCC | 20 | 58.92 | 50.0 | 123 |
| SbPRP-1R | CGGGAATTAATGCCGTCCAT | 20 | 58.40 | 50.00 |
| SbPRP-2F | CAAGAAGCCTGTTGTACCGC | 20 | 59.48 | 55.00 | 130 |
| SbPRP-2R | AGCATGGTTGCTCACAGGAG | 20 | 60.32 | 55.00 |
| SbPRP-3 F | AAGGATGCTACTCGGCGTCT | 20 | 61.04 | 55.0 | 121 |
| SbPRP-3 R | GTTCTTCCTCGTGCAGTCGG | 20 | 61.01 | 60.0 |
| SbPRP-4 F | CGGCAAGGTGCACTACCCAT | 20 | 62.82 | 60.0 | 123 |
| SbPRP-4R | GCCGGCTTCTTGTGGATGTG | 20 | 61.93 | 60.0 |
| SbPRP-5F | CAACTTCATTTGCCGTCGGG | 20 | 60.11 | 55.00 | 131 |
| SbPRP-5R | TCAACAGCCGTTGTACCTCC | 20 | 59.97 | 55.00 |
| SbPRP-6 F | CCAGCAAGCCGCTCTTCTTT | 20 | 61.24 | 55.0 | 120 |
| SbPRP-6 R | TTGACGTCGTTGGGGGAAC | 20 | 61.94 | 55.0 |
| SbPRP-7F | CAAGGACAAGGACGACCCCA | 20 | 61.76 | 60.00 | 139 |
| SbPRP-7R | GTCTTTGGAGCCCGGAGGA | 19 | 61.29 | 63.16 |
| SbPRP-8F | GGGGGTGTTCTACGTGGTGT | 20 | 61.76 | 60.00 | 150 |
| SbPRP-8R | GCGATCTTGTCGTTGGGGTT | 20 | 60.95 | 55.00 |
| SbPRP-9F | CAGCCTCTTCTTGCCTGTCA | 20 | 59.96 | 55.00 | 153 |
| SbPRP-9R | TCGGCGGCTTAGCTGAATAG | 20 | 59.97 | 55.00 |
| SbPRP-10 F | CAAGGACAAGCAACCGGCAA | 20 | 61.73 | 55.0 | 131 |
| SbPRP-10 R | ACTTCACACAAGCCACCCCA | 20 | 61.93 | 55.0 |
| SbPRP-11F | GCGTCGACTGGAGGCTTTAT | 20 | 60.18 | 55.00 | 159 |
| SbPRP-11R | CGGCAGAAAGTACGGAAGGT | 20 | 60.04 | 55.00 |
| SbPRP-12F | AGAATGGTGACCAGTGGCTG | 20 | 59.96 | 55.00 | 157 |
| SbPRP-12R | CTTGGGCTCAAAGGCCCATA | 20 | 60.03 | 55.00 |
| SbPRP-13 F | AGTGCCACCTTCAGATCCTGC | 21 | 62.40 | 57.14 | 136 |
| SbPRP-13 R | CAGAGTCACCAGCCTAGACCAG | 22 | 61.79 | 59.09 |
| SbPRP-14 F | AAGAAGCCTGAGCCGTCAAA | 20 | 59.89 | 50 | 128 |
| SbPRP-14 R | TGCATGTTCATTGCAGGAGG | 20 | 58.82 | 50 |
| SbPRP-15 F | AAGAAGACCTTCGTCGCAG | 20 | 59.33 | 50 | 140 |
| SbPRP-15 R | TTCTTGTGGATGTGAGGGCT | 20 | 58.93 | 50 |
| SbPRP-16 F | ACTACCCCCTGATCGACTTG | 20 | 58.51 | 55 | 137 |
| SbPRP-16 R | CGTTCCGCACAGATTGATGT | 20 | 58.92 | 50 |
| SbPRP-17 F | GAAGAAGGCAGACGTGGTTG | 20 | 58.13 | 55 | 130 |
| SbPRP-17 R | CATCTCGTAGCTCTCGTCGT | 20 | 58.07 | 55 |
| SbPRP-18 F | GTCAACGTCCACAGTAACCG | 20 | 58.87 | 55 | 133 |
| SbPRP-18 R | CGGCTTAGCTGAATAGCTGC | 20 | 59.1 | 55 |
| SbPRP-19 F | TAAGCAGCACAAGTTTCGCC | 20 | 59.41 | 50 | 122 |
| SbPRP-19 R | TACGTAGACTCGGACAAGGC | 20 | 58.91 | 55 |
| SbPRP-20 F | AGCCACAACCCAAACCGGAA | 20 | 60 | 55 | 126 |
| SbPRP-20 R | GCTCTGGATGAGGCTCAGTT | 20 | 58 | 55 |
| SbPRP-21 F | TAATCCGAGACGACGCAAGA | 20 | 58.9 | 50 | 120 |
| SbPRP-21R | GATCCCAACCTTCTCGGTGA | 20 | 59.1 | 55 |
| SbHYPRP-1F | GGCGTGTCCCCAATAGTTCT | 20 | 59.75 | 55 | 124 |
| SbHYPRP-1R | GTAGGCATCAAGCAAGCAGC | 20 | 59.90 | 55 |
| SbHYPRP-2F | CGTGTGATCGAGCGCAACTA | 20 | 60.80 | 55 | 152 |
| SbHYPRP-2R | ACCCGTACGACAACTTGAGAG | 21 | 59.73 | 52.38 |
| SbHYPRP-3F | AGGCTGCTCTCTTCCTTGCC | 20 | 62.20 | 60.0 | 131 |
| SbHYPRP-3R | CCCACCATGGCTGTGGGTC | 19 | 62.94 | 68.0 |
| SbHYPRP-4F | GTGCCGACTCTTGCATTTGCC | 21 | 62.98 | 57.14 | 140 |
| SbHYPRP-4R | GGGGCAAGCTGGATGATGTAACT | 23 | 62.84 | 52.17 |
| SbHYPRP-5F | CCGACACCACCATCATCTCC | 20 | 60.18 | 60.00 | 155 |
| SbHYPRP-5R | TTGCCGATGGAGACCTTGAC | 20 | 60.04 | 55.00 |
| SbHYPRP-6F | CAACGTGCTGAACGGGCTGA | 20 | 63.59 | 60.0 | 135 |
| SbHYPRP-6R | CCCAGGATGTTGGCTCGCAG | 20 | 63.49 | 65.0 |
| SbHYPRP-7F | TCATCAAGGCCAAGGTGGG | 19 | 59.62 | 57.89 | 139 |
| SbHYPRP-7R | ATGGGGAGGTTGAGGTGGAT | 20 | 60.25 | 55.00 |
| SbHYPRP-8F | ACACCTTGCTACAAGGCTCA | 20 | 59.24 | 50.00 | 160 |
| SbHYPRP-8R | GGCTCAACTTCGGTTGACAT | 20 | 58.48 | 50.00 |
| SbHYPRP-9F | GCAGTACGCAACTACGCTGT | 20 | 61.01 | 55.0 | 140 |
| SbHYPRP-9R | GACTGTGGGCTGCAATTGATCT | 22 | 61.20 | 50.0 |
| SbHYPRP-10F | AGACTACCTCTAGCCCTTCGG | 21 | 60.13 | 57.14 | 141 |
| SbHYPRP-10R | CCTTCTTCTCGCAGCTCTGG | 20 | 60.46 | 60.00 |
| SbHYPRP-11F | GGTCTGGCATCCAATTATGTCG | 22 | 59.45 | 50.00 | 127 |
| SbHYPRP-11R | CGTCATCAACCAAGACACCATC | 22 | 59.58 | 50.00 |
| SbHYPRP-12F | CATCGGCGAGAAACCAATGC | 20 | 60.25 | 55.00 | 154 |
| SbHYPRP-12R | AAGCTTCGGAATGGGTCGAG | 20 | 60.11 | 55.00 |
| SbHYPRP-13 F | CGTGTCCCCAATAGTTCTGC | 20 | 58.63 | 55 | 125 |
| SbHYPRP-13 R | GAGGTAGGCATCAAGCAAGC | 20 | 58.98 | 55 |
| SbHYPRP-14 F | GCATTGTGCCTCTGCACTG | 19 | 59.79 | 57.89 | 123 |
| SbHYPRP-14 R | GTAGCAAGTGAAGTCCTCCG | 20 | 58.0 | 55 |
| SbHYPRP-15 F | GTGTGCCGACTCTTGCATT | 19 | 59.85 | 52.63 | 140 |
| SbHYPRP-15 R | GGCAAGCTGGATGATGTAAC | 20 | 58.17 | 50 |
| SbHYPRP-16 F | GTGACGACGACGATGATGAG | 20 | 58.54 | 55 | 123 |
| SbHYPRP-16 R | AGGAGATGATGGTGGTGTCG | 20 | 59.17 | 55 |
| SbHYPRP-17 F | CCTCTGCACTGCCATCAAG | 19 | 58.52 | 57.89 | 120 |
| SbHYPRP-17 R | TAGTGGGTGCAGTGGAAGC | 19 | 59.63 | 57.89 |
| SbHYPRP-18 F | CACAAAGGCCATGGAAAGAG | 20 | 58.48 | 50 | 133 |
| SbHYPRP-18 R | CGACATAATTGGATGCCAGACC | 22 | 59.45 | 50 |
| SbHYPRP-19 F | TTGCTCCTCGTGGCCCTCAA | 20 | 59.6 | 60 | 121 |
| SbHYPRP-19 R | ACTTGCCGCCGGTTGAAGGT | 20 | 59.6 | 60 |
| SbHYPRP-20 F | TGATCAAGGCTCAGGTCGG | 19 | 59.1 | 57.89 | 138 |
| SbHYPRP-20 R | GTGGCACGTTGAGGTTGATG | 20 | 59.76 | 55 |
| SbHYPRP-21F | CCGTTGTCGATAGCTGAGGAA | 21 | 59.87 | 52.38 | 151 |
| SbHYPRP-21R | AGGACAGTGTAACGATGGCG | 20 | 60.11 | 55.00 |
| SbHYPRP-22F | CAAGACATCGAAGCCTCTGC | 20 | 58.99 | 55 | 126 |
| SbHYPRP-22R | GCCGTTACCGCCATGTTTAT | 20 | 58.98 | 50 |
| SbHYPRP-23F | CGTGCTGAACCTACTGAAGC | 20 | 58.93 | 55 | 138 |
| SbHYPRP-23R | TTGAGGTTGATGCCCAGGAT | 20 | 59 | 50 |
| SbHYPRP-24F | CCTGATCAAGGCCAAGGTG | 19 | 58.12 | 57.89 | 124 |
| SbHYPRP-24R | GATGCCGAGGATATTGCCCT | 20 | 59.67 | 55 |
| SbHYPRP-25F | GCTCACCCTCAACTTCGACT | 20 | 59.68 | 55 | 128 |
| SbHYPRP-25R | TTAGATTGTGCCGGATGGCT | 20 | 59.45 | 50 |
| SbHYPRP-26F | ACCATGCTGCACATTGATCC | 20 | 58.89 | 50 | 130 |
| SbHYPRP-26R | GTAGTTGACGAGGAGGCTGA | 20 | 58.83 | 55 |
| SbHYPRP-27F | TCACCGGATCTGCTTGTCAT | 20 | 59.10 | 50 | 135 |
| SbHYPRP-27R | CAATGGTGTTTGGAGGAGGC | 20 | 59.11 | 55 |
| SbACP2F | ACGAACTTGTTGCGGCAGAAG | 21 | 58.5 | 52.4 | 110 |
| SbACP2R | GAACAAGAAGGGATGCGCTGG | 21 | 58.8 | 57.1 |
| SbEF-PF | TGAAGCGGGTGAGAAGATTGT | 21 | 56.5 | 47.6 | 114 |
| SbEF-PR | AGCCAAATCATACTCGCCCA | 20 | 56.7 | 50 |
