# Supplementary material for: Identification and analysis of proline-rich proteins and hybrid proline-rich proteins super family genes from Sorghum bicolor and their expression patterns to abiotic stress and zinc stimuli
Source: Front Plant Sci. 2022 Sep 26;13:952732. doi: 10.3389/fpls.2022.952732 (PMC9549341; doi:10.3389/fpls.2022.952732)
Supplement: Supplementary file 14 [file Table_2.doc]

| Gene | ARR1AT | ACGTATE | ERELEE4 | GT1CONSE | POLLEN1LE | TAAAGSTK | EECCRCAH1 | HDZIP2ATA | BIHD1OS | TELOBX | DPBFC | RBCSCONS | MYCCONSE | ABRELATE | IRO2OS | AMMORE | T/GBOXA | LEAFYAT | E2FCON | SP8BFIB | SEF4MOTIF | CACTFTPPC | L1BOXATP1 | CBFHV | MYBCORE | ANAERO1C | XYLAT | IBOX | IBOXCORE | ASF1MOTIF | WRKY71OS | SURECORE | WBOXNTE | WBOXHVIS |
| --- | --- | --- | --- | --- | --- | --- | --- | --- | --- | --- | --- | --- | --- | --- | --- | --- | --- | --- | --- | --- | --- | --- | --- | --- | --- | --- | --- | --- | --- | --- | --- | --- | --- | --- |
| SbPRP-1 | 20 | 2 | 2 | 16 | 13 | 13 | 3 | 2 | 0 | 0 | 0 | 16 | 16 | 1 | 1 | 0 | 1 | 2 | 0 | 2 | 5 | 20 | 1 | 2 | 3 | 1 | 1 | 1 | 1 | 1 | 2 | 2 | 1 | 5 |
| SbPRP-2 | 9 | 4 | 0 | 4 | 3 | 6 | 2 | 2 | 1 | 1 | 10 | 0 | 20 | 0 | 3 | 1 | 10 | 6 | 0 | 0 | 1 | 24 | 0 | 0 | 14 | 1 | 0 | 4 | 32 | 16 | 1 | 1 | 3 | 0 |
| SbPRP-3 | 15 | 1 | 1 | 10 | 12 | 3 | 4 | 0 | 6 | 0 | 7 | 0 | 16 | 0 | 2 | 2 | 0 | 1 | 4 | 0 | 4 | 35 | 1 | 3 | 11 | 1 | 0 | 1 | 0 | 22 | 0 | 5 | 2 | 1 |
| SbPRP-4 | 9 | 2 | 0 | 9 | 4 | 6 | 5 | 2 | 5 | 0 | 4 | 0 | 8 | 24 | 1 | 6 | 2 | 0 | 6 | 0 | 0 | 29 | 1 | 1 | 6 | 0 | 0 | 15 | 3 | 15 | 0 | 0 | 0 | 2 |
| SbPRP-5 | 14 | 8 | 1 | 10 | 4 | 5 | 4 | 0 | 8 | 0 | 2 | 1 | 8 | 0 | 0 | 1 | 0 | 6 | 8 | 12 | 1 | 23 | 0 | 10 | 15 | 1 | 0 | 17 | 0 | 1 | 12 | 0 | 11 | 4 |
| SbPRP-6 | 11 | 3 | 1 | 14 | 11 | 4 | 4 | 0 | 3 | 0 | 2 | 0 | 16 | 3 | 0 | 2 | 0 | 0 | 0 | 1 | 3 | 30 | 1 | 1 | 10 | 2 | 1 | 0 | 2 | 1 | 1 | 1 | 3 | 0 |
| SbPRP-7 | 3 | 0 | 0 | 3 | 1 | 1 | 1 | 0 | 5 | 0 | 4 | 0 | 8 | 0 | 0 | 0 | 0 | 1 | 0 | 6 | 0 | 16 | 0 | 1 | 5 | 0 | 0 | 0 | 5 | 1 | 0 | 2 | 2 | 3 |
| SbPRP-8 | 24 | 36 | 0 | 24 | 16 | 11 | 3 | 0 | 5 | 0 | 6 | 0 | 22 | 6 | 1 | 0 | 2 | 1 | 0 | 0 | 4 | 55 | 0 | 6 | 16 | 0 | 0 | 8 | 6 | 12 | 25 | 6 | 0 | 0 |
| SbPRP-9 | 36 | 20 | 0 | 20 | 10 | 6 | 6 | 0 | 11 | 0 | 16 | 1 | 42 | 0 | 1 | 1 | 1 | 0 | 8 | 2 | 7 | 53 | 1 | 5 | 15 | 5 | 0 | 12 | 11 | 5 | 26 | 9 | 10 | 5 |
| SbPRP-10 | 22 | 8 | 0 | 12 | 10 | 4 | 4 | 0 | 3 | 0 | 9 | 0 | 0 | 0 | 0 | 1 | 2 | 2 | 2 | 0 | 5 | 36 | 0 | 7 | 7 | 2 | 1 | 4 | 4 | 2 | 11 | 5 | 6 | 1 |
| SbPRP-11 | 27 | 6 | 1 | 20 | 8 | 9 | 0 | 0 | 6 | 0 | 2 | 3 | 22 | 0 | 1 | 0 | 0 | 3 | 6 | 0 | 5 | 34 | 1 | 0 | 3 | 0 | 0 | 8 | 8 | 0 | 13 | 2 | 7 | 3 |
| SbPRP-12 | 15 | 4 | 1 | 12 | 12 | 7 | 1 | 1 | 3 | 0 | 1 | 0 | 6 | 0 | 0 | 0 | 0 | 1 | 4 | 2 | 8 | 25 | 0 | 3 | 4 | 1 | 0 | 3 | 3 | 0 | 8 | 3 | 5 | 1 |
| SbPRP-13 | 22 | 4 | 0 | 11 | 14 | 1 | 3 | 0 | 3 | 0 | 4 | 0 | 20 | 0 | 4 | 21 | 0 | 0 | 0 | 1 | 6 | 17 | 0 | 3 | 6 | 2 | 0 | 4 | 3 | 2 | 9 | 1 | 4 | 2 |
| SbPRP-14 | 16 | 10 | 0 | 1 | 2 | 1 | 3 | 1 | 1 | 0 | 3 | 0 | 16 | 2 | 0 | 0 | 1 | 0 | 5 | 0 | 0 | 12 | 9 | 2 | 10 | 1 | 0 | 2 | 1 | 5 | 15 | 2 | 9 | 3 |
| SbPRP-15 | 12 | 6 | 0 | 1 | 2 | 1 | 0 | 1 | 1 | 0 | 0 | 1 | 8 | 1 | 0 | 0 | 0 | 1 | 2 | 1 | 0 | 12 | 0 | 0 | 5 | 1 | 0 | 2 | 2 | 2 | 6 | 0 | 3 | 3 |
| SbPRP-16 | 9 | 12 | 0 | 3 | 6 | 3 | 2 | 0 | 3 | 0 | 5 | 0 | 10 | 4 | 0 | 0 | 1 | 0 | 0 | 0 | 4 | 34 | 0 | 3 | 12 | 1 | 0 | 1 | 1 | 1 | 11 | 3 | 7 | 5 |
| SbPRP-17 | 8 | 6 | 0 | 2 | 4 | 1 | 1 | 0 | 2 | 0 | 4 | 0 | 5 | 1 | 0 | 0 | 2 | 1 | 1 | 1 | 3 | 15 | 0 | 2 | 8 | 1 | 0 | 0 | 6 | 0 | 20 | 4 | 5 | 4 |
| SbPRP-18 | 0 | 1 | 0 | 1 | 2 | 0 | 0 | 0 | 0 | 1 | 1 | 0 | 0 | 0 | 0 | 0 | 0 | 0 | 0 | 0 | 2 | 13 | 0 | 0 | 0 | 0 | 0 | 0 | 3 | 0 | 0 | 0 | 0 | 0 |
| SbPRP-19 | 12 | 6 | 0 | 1 | 2 | 1 | 0 | 1 | 1 | 0 | 0 | 1 | 8 | 1 | 0 | 0 | 0 | 1 | 2 | 1 | 0 | 12 | 0 | 0 | 5 | 1 | 0 | 2 | 2 | 2 | 6 | 0 | 3 | 3 |
| SbPRP-20 | 22 | 4 | 0 | 11 | 14 | 1 | 3 | 0 | 3 | 0 | 4 | 0 | 20 | 0 | 4 | 21 | 0 | 0 | 0 | 1 | 6 | 17 | 0 | 3 | 6 | 2 | 0 | 4 | 3 | 2 | 9 | 1 | 4 | 2 |
| SbPRP-21 | 16 | 10 | 0 | 1 | 2 | 1 | 3 | 1 | 1 | 0 | 3 | 0 | 16 | 2 | 0 | 0 | 1 | 8 | 3 | 2 | 0 | 12 | 9 | 2 | 10 | 1 | 0 | 2 | 1 | 5 | 15 | 2 | 9 | 3 |

**Table S2.** Conserved *cis-*acting regulatory elements in PRP promoters of *Sorghum*

(ARR1: regulator, ACGTATE: etiolation, ERE: ethylene responsive, GT1: light, DPBF: drought, POLLEN1LE:Pollen specific activation, TAAAGSTK: cell specific expression, EEC: binding site, HDZ: light, BIHD1OS: transcription factor, TELOBX: promoter, RBCS: consensus sequence, MYC :dehydration, ABRE: ABA responsive , IRO: binding core, T/GBOXA: JA induction , LEAFYAT: target sequence, E2F: consensous sequence, SP8B: binding site, SEF4M: binding site, CACTF: regulator, L1BOX: Layer specific expression, CBFHV : dehydration, MYB: water stress responsive, ANAERO1C: fermentation, XYLAT: promoter, IBOX: light, IBOXCORE: phytochrome regulation, ASF1: light, WRKY: repressor, SURE: sulfur responsive, WBOXNTE: repressor, WBOXHVIS: sugar responsive)
