# Supplementary material for: Identification and analysis of proline-rich proteins and hybrid proline-rich proteins super family genes from Sorghum bicolor and their expression patterns to abiotic stress and zinc stimuli
Source: Front Plant Sci. 2022 Sep 26;13:952732. doi: 10.3389/fpls.2022.952732 (PMC9549341; doi:10.3389/fpls.2022.952732)
Supplement: Supplementary file 16 [file Table_4.doc]

| SbPRP Gene 1 | Chr | SbPRP Paralog Gene 2 | Chr | No. non Synonymous sites (N) | No. Synonymous sites (S) | Non Synonymous substitution rate (d_N_) | Synonymous substitution rate (d_S_) | d_N_ / d_S_ |
| --- | --- | --- | --- | --- | --- | --- | --- | --- |
| SbPRP1 | 1 | SbPRP2 | 1 | 787.7 | 184.3 | 6.4127 | 0.0648 | 99.0000 |
| SbPRP4 | 1 | SbPRP6 | 1 | 617.4 | 159.6 | 3.9701 | 0.9337 | 4.2522 |
| SbPRP9 | 3 | SbPRP18 | 7 | 165.8 | 41.2 | 2.9704 | 7.9775 | 0.3723 |
| SbPRP11 | 4 | SbPRP13 | 5 | 1186.6 | 256.4 | 15.7745 | 0.1593 | 99.0000 |
| SbPRP16 | 6 | SbPRP20 | 10 | 353.1 | 90.9 | 10.7601 | 2.2641 | 4.7524 |

**Table S4:** Non-synonymous to synonymous substitution ratios of *SbPRP* paralogs

(d_N_/d_S_ >1 = Positive or Darwinian Selection (Driving Change); d_N_/d_S_ <1 = Purifying or Stabilizing Selection (Acting against change); d_N_ /d_S_ =1 Neutral Selection)
