# Supplementary material for: Identification and analysis of proline-rich proteins and hybrid proline-rich proteins super family genes from Sorghum bicolor and their expression patterns to abiotic stress and zinc stimuli
Source: Front Plant Sci. 2022 Sep 26;13:952732. doi: 10.3389/fpls.2022.952732 (PMC9549341; doi:10.3389/fpls.2022.952732)
Supplement: Supplementary file 17 [file Table_5.doc]

**Table S5.** Non-synonymous to synonymous substitution ratios of *PRP* orthologs of *Sorghum, Oryza* and *Arabidopsis*

| SbPRP Gene | Chr | Ortholog | Chr | No. non Synonymous sites (N) | No. Synonymous sites (S) | Non Synonymous substitution rate (d_N_) | Synonymous substitution rate (d_S_) | d_N_ / d_S_ |
| --- | --- | --- | --- | --- | --- | --- | --- | --- |
| SORBI_3001G438500 | 1 | Os02G0732800 | 2 | 614.4 | 162.6 | 3.2729 | 2.7659 | 1.1833 |
| SORBI_3008G082400 | 8 | Os05G0226000 | 5 | 514.4 | 100.6 | 11.4668 | 0.1158 | 99.0000 |
| SORBI_3006G211701 | 6 | Os06G0104800 | 6 | 286.2 | 79.8 | 2.3215 | 51.2705 | 0.0453 |
| SORBI_3010G054600 | 10 | Os06G0168700 | 6 | 543.1 | 194.9 | 9.8793 | 21.6987 | 0.4553 |
| SORBI_3001G266100 | 1 | At5G59170 | 5 | 663.9 | 200.1 | 4.8872 | 39.9169 | 0.1224 |

(d_N_/d_S_ >1 = Positive or Darwinian Selection (Driving Change); d_N_/d_S_ <1 = Purifying or Stabilizing Selection (Acting against change); d_N_ /d_S_ =1 Neutral Selection)
