# Supplementary material for: Identification and analysis of proline-rich proteins and hybrid proline-rich proteins super family genes from Sorghum bicolor and their expression patterns to abiotic stress and zinc stimuli
Source: Front Plant Sci. 2022 Sep 26;13:952732. doi: 10.3389/fpls.2022.952732 (PMC9549341; doi:10.3389/fpls.2022.952732)
Supplement: Supplementary file 18 [file Table_6.doc]

**Table S6.** Non-synonymous to synonymous substitution ratios of *SbHyPRP* paralogs

| SbHyPRP Gene 1 | Chr | SbHyPRP Paralog Gene 2 | Chr | No. non Synonymous sites (N) | No. Synonymous sites (S) | Non Synonymous substitution rate (d_N_) | Synonymous substitution rate (d_S_) | d_N_ / d_S_ |
| --- | --- | --- | --- | --- | --- | --- | --- | --- |
| SbHyPRP2 | 1 | SbHyPRP13 | 3 | 303.5 | 89.5 | 16.7837 | 0.1695 | 99.0000 |
| SbHyPRP3 | 1 | SbHyPRP17 | 5 | 305.1 | 81.9 | 2.0944 | 1.8178 | 1.1522 |
| SbHyPRP9 | 1 | SbHyPRP21 | 6 | 888.7 | 251.3 | 5.2161 | 2.9133 | 1.7904 |
| SbHyPRP10 | 3 | SbHyPRP22 | 7 | 350.1 | 93.9 | 5.9268 | 0.0599 | 99.0000 |
| SbHyPRP11 | 3 | SbHyPRP12 | 3 | 402.2 | 110.8 | 16.5341 | 0.1670 | 99.0000 |
| SbHyPRP14 | 3 | SbHyPRP15 | 4 | 301.1 | 64.9 | 0.0067 | 0.0159 | 0.4199 |
| SbHyPRP18 | 6 | SbHyPRP19 | 6 | 315.3 | 80.7 | 1.9047 | 56.3642 | 0.0338 |
| SbHyPRP23 | 9 | SbHyPRP24 | 9 | 552.9 | 134.1 | 3.8559 | 2.5476 | 1.5136 |
| SbHyPRP26 | 10 | SbHyPRP27 | 10 | 688.7 | 238.3 | 17.3921 | 0.3093 | 56.2218 |

(d_N_/d_S_ >1 = Positive or Darwinian Selection (Driving Change); d_N_/d_S_ <1 = Purifying or Stabilizing Selection (Acting against change); d_N_ /d_S_ =1 Neutral Selection)
