# Supplementary material for: Identification and analysis of proline-rich proteins and hybrid proline-rich proteins super family genes from Sorghum bicolor and their expression patterns to abiotic stress and zinc stimuli
Source: Front Plant Sci. 2022 Sep 26;13:952732. doi: 10.3389/fpls.2022.952732 (PMC9549341; doi:10.3389/fpls.2022.952732)
Supplement: Supplementary file 19 [file Table_7.doc]

**Table S7.** Non-synonymous to synonymous substitution ratios of *SbHyPRP* orthologs of *Sorghum, Oryza* and *Arabidopsis*

| SbHyPRP Gene | Chr | Ortholog | Chr | No. non Synonymous sites (N) | No. Synonymous sites (S) | Non Synonymous substitution rate (d_N_) | Synonymous substitution rate (d_S_) | d_N_ / d_S_ |
| --- | --- | --- | --- | --- | --- | --- | --- | --- |
| SORBI_3010G204700 | 10 | At2G10940 | 2 | 692.0 | 181.0 | 13.0733 | 12.7199 | 1.0278 |
| SORBI_3010G054800 | 10 | Os06G07220 | 6 | 543.1 | 194.9 | 13.1998 | 1.6593 | 7.9548 |
| SORBI_3006G211701 | 6 | Os04G52260 | 4 | 342.8 | 101.2 | 16.6930 | 0.4852 | 34.4035 |
| SORBI_3001G304201 | 1 | Os10G40420 | 10 | 382.5 | 103.5 | 2.0403 | 53.5230 | 0.0381 |
| SORBI_3004G291500 | 4 | Os02G44320 | 2 | 300.9 | 83.1 | 4.9100 | 0.6614 | 7.4237 |
| SORBI_3010G003600 | 10 | Os06G01580 | 6 | 279.0 | 69.0 | 8.0831 | 3.7397 | 2.1615 |
| SORBI_3001G541400 | 1 | Os03G01320 | 3 | 424.7 | 100.3 | 1.9909 | 1.5915 | 1.2509 |
| SORBI_3001G302600 | 1 | Os10G40614 | 10 | 395.1 | 105.9 | 13.8882 | 9.6873 | 1.4337 |
| SORBI_3001G112200 | 1 | Os03G50960 | 3 | 291.2 | 77.8 | 3.6410 | 48.0054 | 0.0758 |
| SORBI_3006G238100 | 6 | Os04G55170 | 4 | 209.6 | 57.4 | 10.1718 | 0.1027 | 99.0000 |
| SORBI_3003G329400 | 3 | Os03G14654 | 3 | 385.8 | 88.2 | 9.2432 | 0.0934 | 99.0000 |

(d_N_/d_S_ >1 = Positive or Darwinian Selection (Driving Change); d_N_/d_S_ <1 = Purifying or Stabilizing Selection (Acting against change);

d_N_ /d_S_ =1 Neutral Selection)
