# Supplementary material for: Identification and analysis of proline-rich proteins and hybrid proline-rich proteins super family genes from Sorghum bicolor and their expression patterns to abiotic stress and zinc stimuli
Source: Front Plant Sci. 2022 Sep 26;13:952732. doi: 10.3389/fpls.2022.952732 (PMC9549341; doi:10.3389/fpls.2022.952732)
Supplement: Supplementary file 21 [file Table_9.doc]

| Name | Phosphorylation sites | | | PKC | CK2 | RSK | PKA | UNSP | EGFR | INSR | PKG | CK1 | DNAPK | CdC2 | p38MAPK | CDK5 | GSK3 | ATM |
| --- | --- | --- | --- | --- | --- | --- | --- | --- | --- | --- | --- | --- | --- | --- | --- | --- | --- | --- |
|  | S | T | Y |  |  |  |  |  |  |  |  |  |  |  |  |  |  |  |
| SbHyPRP-1 | 14 | 8 | 1 | 9 | 1 | 0 | 5 | 7 | 0 | 1 | 0 | 4 | 1 | 2 | 0 | 0 | 0 | 1 |
| SbHyPRP-2 | 14 | 5 | 1 | 6 | 0 | 0 | 2 | 6 | 0 | 0 | 1 | 1 | 2 | 5 | 2 | 3 | 1 | 0 |
| SbHyPRP-3 | 5 | 3 | 0 | 1 | 0 | 0 | 3 | 0 | 0 | 0 | 0 | 0 | 0 | 1 | 0 | 0 | 2 | 0 |
| SbHyPRP-4 | 15 | 4 | 4 | 2 | 1 | 0 | 8 | 14 | 0 | 1 | 0 | 0 | 0 | 2 | 2 | 2 | 1 | 1 |
| SbHyPRP-5 | 12 | 6 | 0 | 3 | 0 | 0 | 3 | 8 | 0 | 0 | 0 | 0 | 0 | 5 | 3 | 3 | 5 | 0 |
| SbHyPRP-6 | 6 | 5 | 0 | 3 | 0 | 0 | 4 | 2 | 0 | 0 | 0 | 1 | 0 | 1 | 1 | 3 | 3 | 0 |
| SbHyPRP-7 | 3 | 3 | 1 | 2 | 1 | 0 | 0 | 3 | 0 | 0 | 0 | 0 | 0 | 1 | 0 | 0 | 0 | 0 |
| SbHyPRP-8 | 31 | 16 | 2 | 16 | 3 | 1 | 10 | 32 | 1 | 0 | 1 | 1 | 2 | 1 | 10 | 1 | 3 | 0 |
| SbHyPRP-9 | 33 | 24 | 3 | 24 | 6 | 2 | 5 | 39 | 0 | 1 | 4 | 2 | 3 | 4 | 5 | 3 | 4 | 2 |
| SbHyPRP-10 | 10 | 9 | 4 | 6 | 0 | 1 | 2 | 12 | 0 | 1 | 1 | 0 | 2 | 7 | 2 | 3 | 1 | 1 |
| SbHyPRP-11 | 48 | 13 | 2 | 19 | 4 | 1 | 9 | 36 | 1 | 1 | 3 | 4 | 3 | 11 | 1 | 3 | 3 | 0 |
| SbHyPRP-12 | 48 | 12 | 4 | 19 | 6 | 1 | 9 | 36 | 1 | 1 | 3 | 4 | 1 | 11 | 1 | 3 | 3 | 0 |
| SbHyPRP-13 | 14 | 3 | 1 | 8 | 1 | 0 | 4 | 12 | 0 | 0 | 4 | 0 | 2 | 4 | 0 | 0 | 0 | 2 |
| SbHyPRP-14 | 7 | 7 | 0 | 0 | 0 | 0 | 2 | 4 | 0 | 0 | 0 | 1 | 0 | 2 | 3 | 5 | 3 | 0 |
| SbHyPRP-15 | 8 | 5 | 0 | 3 | 0 | 0 | 1 | 10 | 0 | 0 | 0 | 2 | 0 | 2 | 2 | 3 | 2 | 0 |
| SbHyPRP-16 | 7 | 3 | 0 | 2 | 0 | 0 | 1 | 8 | 0 | 0 | 0 | 1 | 0 | 0 | 2 | 3 | 2 | 0 |
| SbHyPRP-17 | 31 | 29 | 4 | 28 | 5 | 1 | 8 | 40 | 0 | 0 | 1 | 1 | 2 | 10 | 3 | 0 | 0 | 2 |
| SbHyPRP-18 | 3 | 4 | 0 | 2 | 0 | 0 | 1 | 3 | 0 | 0 | 0 | 0 | 0 | 0 | 3 | 2 | 0 | 0 |
| SbHyPRP-19 | 4 | 6 | 0 | 4 | 0 | 0 | 3 | 4 | 0 | 0 | 0 | 0 | 0 | 0 | 2 | 5 | 3 | 0 |
| SbHyPRP-20 | 19 | 13 | 2 | 11 | 0 | 1 | 0 | 15 | 0 | 0 | 1 | 0 | 0 | 3 | 0 | 6 | 4 | 0 |
| SbHyPRP-21 | 9 | 12 | 0 | 6 | 0 | 0 | 4 | 10 | 0 | 0 | 1 | 1 | 0 | 3 | 3 | 5 | 5 | 0 |
| SbHyPRP-22 | 22 | 14 | 2 | 18 | 2 | 1 | 7 | 17 | 1 | 0 | 0 | 2 | 0 | 9 | 0 | 2 | 0 | 0 |
| SbHyPRP-23 | 19 | 4 | 0 | 2 | 0 | 0 | 2 | 14 | 0 | 0 | 0 | 1 | 0 | 8 | 1 | 2 | 2 | 0 |
| SbHyPRP-24 | 13 | 7 | 0 | 4 | 0 | 0 | 2 | 0 | 6 | 0 | 0 | 0 | 5 | 0 | 5 | 2 | 2 | 0 |
| SbHyPRP-25 | 15 | 4 | 0 | 7 | 0 | 2 | 5 | 10 | 0 | 0 | 1 | 1 | 1 | 8 | 3 | 2 | 1 | 0 |
| SbHyPRP-26 | 21 | 16 | 20 | 5 | 0 | 0 | 9 | 45 | 9 | 1 | 0 | 0 | 1 | 4 | 12 | 19 | 19 | 0 |
| SbHyPRP-27 | 14 | 18 | 3 | 6 | 2 | 0 | 0 | 23 | 0 | 0 | 2 | 0 | 1 | 6 | 7 | 12 | 8 | 0 |

PKC: Protein Kinase C, CK2: Casein Kinase 2, RSK1: Ribosomal S6 Kinase 1, PKA: Protein Kinase A, CK1: Casein Kinase, DNAPK: DNA dependant Protein Kinase, ATM: Ataxia-telangiectasia mutated **kinase**, EGFR: epidermal growth factor receptor, INSR: insulin receptor tyrosine **kinase, PKG:** Protein **Kinase** G, CDK: Cyclin dependent kinases.

**Table S8.** Types of protein kinases in the phosphorylation of SbHyPRPs
