# Supplementary material for: Identification and analysis of proline-rich proteins and hybrid proline-rich proteins super family genes from Sorghum bicolor and their expression patterns to abiotic stress and zinc stimuli
Source: Front Plant Sci. 2022 Sep 26;13:952732. doi: 10.3389/fpls.2022.952732 (PMC9549341; doi:10.3389/fpls.2022.952732)
Supplement: Supplementary file 22 [file Table_10.doc]

**Table S10.** Homology modeling and structure validation of SbHyPRP and SbPRP proteins using Swiss-Model and Protein Structure Validation Suite (PSVS) respectively

| **Homology modeling** | | | | | **Structure validation** |
| --- | --- | --- | --- | --- | --- |
| **Protein** | **Template PDB ID** | **Template description** | **Chain** | **Model oligo-state** | **Ramachandran plot**  **(Statistics)** |
| SbPRP3 | 6jmt.1.A | ARF GTPase-activating protein GIT2 | A | Monomer | Favoured region: 82.8%,  Allowed region: 17.2%,  Generously region: 0.0%,  Disallowed region: 0.0% |
| SbPRP6 | 4z8w.1.A | Major pollen allergen Pla l 1 | A | Monomer | Favoured region: 84.0%,  Allowed region: 16.0%,  Generously region: 0.0%,  Disallowed region: 0.0% |
| SbPRP10 | 4e7j.1.A | Pro-Pol polyprotein  PFV integrase Target Capture Complex | All chains | Homo-tetramer | Favoured region: 81.5%,  Allowed region: 16.2%,  Generously region: 1.3%,  Disallowed region: 1.0% |
| SbPRP12 | 7s7b.1.F | Zinc finger CCHC domain-containing protein 8 | F | Monomer | Favoured region: 70.3%,  Allowed region: 27.4%,  Generously region: 2.3%,  Disallowed region: 0.0% |
| SbPRP17 | 6xy9.1.A | Haloalkane dehalogenase | All Chains | Homo-dimer | Favoured region: 82.2%,  Allowed region: 14.7%,  Generously region: 1.6%,  Disallowed region: 1.6% |
| SbPRP19 | 6giq.1.2 | Cytochrome c oxidase subunit 7A | Chain 2 | Monomer | Favoured region: 100.0%,  Allowed region: 0.0%,  Generously region: 0.0%,  Disallowed region: 0.0% |
| SbHyPRP2 | 1hyp.1.A | Hydrophobic protein from soybean | A | Monomer | Favoured region: 88.6%,  Allowed region: 10.1%,  Generously region: 1.3%,  Disallowed region: 0.0% |
| SbHyPRP5 | 1hyp.1.A | Hydrophobic protein from soybean | A | Monomer | Favoured region: 91.5%,  Allowed region: 8.5%,  Generously region: 0.0%,  Disallowed region: 0.0% |
| SbHyPRP6 | 1hyp.1.A | Hydrophobic protein from soybean | A | Monomer | Favoured region: 93.1%,  Allowed region: 6.9%,  Generously region: 0.0%,  Disallowed region: 0.0% |
| SbHyPRP13 | 1hyp.1.A | Hydrophobic protein from soybean | A | Monomer | Favoured region: 92.9%,  Allowed region: 7.1%,  Generously region: 0.0%,  Disallowed region: 0.0% |
| SbHyPRP17 | 1hyp.1.A | Hydrophobic protein from soybean | A | Monomer | Favoured region: 87.7%,  Allowed region: 9.6%,  Generously region: 1.4%,  Disallowed region: 1.4% |
| SbHyPRP26 | 1hyp.1.A | Hydrophobic protein from soybean | A | Monomer | Favoured region: 82.2%,  Allowed region: 17.8%,  Generously region: 0.0%,  Disallowed region: 0.0% |
