# Supplementary material for: Identification and analysis of proline-rich proteins and hybrid proline-rich proteins super family genes from Sorghum bicolor and their expression patterns to abiotic stress and zinc stimuli
Source: Front Plant Sci. 2022 Sep 26;13:952732. doi: 10.3389/fpls.2022.952732 (PMC9549341; doi:10.3389/fpls.2022.952732)
Supplement: Supplementary file 23 [file Table_11.doc]

| iRNA_Acc. | Target_Acc. | Expectation  **Table S11.** miRNAs that target *SbPRP* genes | Target_start | Target_end | miRNA_aligned_fragment | Target_aligned_fragment | Inhibition | Multiplicity |
| --- | --- | --- | --- | --- | --- | --- | --- | --- |
| sbi-miR6225-5p | SORBI_3008G082400 | 0 | 1050 | 1073 | AACUAGACUCAAAAGAUUCAUCUC | GAGACGAAUCUUUUGAGUCUAGUU | Cleavage | 1 |
| sbi-miR6220-3p | SORBI_3010G054600 | 0.5 | 1757 | 1780 | AUGCCUUAUAAUUUGGGAUGGAGA | UCUCUAUUCCAAAUUAUAAGGCAU | Cleavage | 1 |
| sbi-miR5568f-3p | SORBI_3010G054600 | 1 | 1758 | 1778 | GUCUUAUAAUUUGGAAUGGAG | CUCUAUUCCAAAUUAUAAGGC | Cleavage | 1 |
| sbi-miR6235-5p | SORBI_3006G211701 | 2 | 439 | 462 | UUGUGAGAGAAAAAUACUGUUGGC | GUCAACAUUGUUUUUCUCUUACAA | Cleavage | 1 |
| sbi-miR6220-3p | SORBI_3004G180900 | 2.5 | 181 | 204 | AUGCCUUAUAAUUUGGGAUGGAGA | CCUUUAUUUCAAAUUAUAAGCCAU | Cleavage | 1 |
| sbi-miR6220-5p | SORBI_3004G180900 | 2.5 | 410 | 433 | CUCCAUCCUAAAUUAUAAGACAUU | AAUGAUUUAUAAUUUGGGAUGAAG | Cleavage | 1 |
| sbi-miR821e | SORBI_3007G074200 | 2.5 | 1405 | 1425 | AAGUCAUCAAAAUAAAAGUUG | CAACUUUUAUUUUGACGGUUU | Cleavage | 1 |
| sbi-miR1435a | SORBI_3004G180900 | 3 | 1477 | 1496 | UUUCUUAAGUCAAACUUUUC | AAAAAGUUUGAUUUUGGAGA | Cleavage | 1 |
| sbi-miR319a | SORBI_3001G266100 | 3 | 262 | 281 | UUGGACUGAAGGGUGCUCCC | GGCAGCACCCUUUAGACCAA | Cleavage | 1 |
| sbi-miR319b | SORBI_3001G266100 | 3 | 262 | 281 | UUGGACUGAAGGGUGCUCCC | GGCAGCACCCUUUAGACCAA | Cleavage | 1 |
| sbi-miR5567 | SORBI_3008G082400 | 3 | 979 | 1002 | UUAAUGAUUCAUGUAUGUGUCCAA | UUAGACGCAUGCAUGAAGUAUUAA | Cleavage | 1 |
| sbi-miR5568d-3p | SORBI_3007G074200 | 3 | 1391 | 1411 | AAAGUUGUGUAUCUAGAAAAG | CUUUUCAAGAUCUACAACUUU | Translation | 2 |
| sbi-miR5568f-3p | SORBI_3004G180900 | 3 | 182 | 202 | GUCUUAUAAUUUGGAAUGGAG | CUUUAUUUCAAAUUAUAAGCC | Cleavage | 1 |
| sbi-miR6225-3p | SORBI_3010G054600 | 3 | 799 | 822 | GAAACGAAUCUUUUAAGUCUAAUU | AACUGGGUUUAAAAGAUUCGUCUU | Cleavage | 1 |
| sbi-miR5385 | SORBI_3001G266300 | 3.5 | 731 | 752 | ACCACCAACCCCACCGCUUCUC | CUGAGGCGGUGGCGAUGGUGGU | Translation | 1 |
| sbi-miR5568g-3p | SORBI_3010G054600 | 3.5 | 1763 | 1783 | AAAACGUCUUAUAAUUUGGAG | UUCCAAAUUAUAAGGCAUUCU | Cleavage | 1 |
| sbi-miR5568g-3p | SORBI_3004G060600 | 3.5 | 1468 | 1488 | AAAACGUCUUAUAAUUUGGAG | UACUGAAAUAUAAGACGUUCU | Cleavage | 1 |
| sbi-miR1435b | SORBI_3004G180900 | 4 | 1477 | 1496 | UUUCUUAAGUCAAACCUUUU | AAAAAGUUUGAUUUUGGAGA | Cleavage | 1 |
| sbi-miR396a | SORBI_3001G438400 | 4 | 996 | 1016 | UUCCACAGCUUUCUUGAACUG | ACCUCUGAGAAGGCUGUGGAG | Cleavage | 1 |
| sbi-miR396b | SORBI_3001G438400 | 4 | 996 | 1016 | UUCCACAGCUUUCUUGAACUG | ACCUCUGAGAAGGCUGUGGAG | Cleavage | 1 |
| sbi-miR396c | SORBI_3001G438400 | 4 | 996 | 1016 | UUCCACAGCUUUCUUGAACUU | ACCUCUGAGAAGGCUGUGGAG | Cleavage | 1 |
| sbi-miR5565e | SORBI_3008G082400 | 4 | 230 | 248 | UUGUUUGGAUGUUGUCGGA | GGCGAAAACAUCCAAGCAG | Cleavage | 1 |
| sbi-miR5567 | SORBI_3007G074200 | 4 | 441 | 464 | UUAAUGAUUCAUGUAUGUGUCCAA | UACGGCACAUGCAUAGAGCAUUAA | Translation | 1 |
| sbi-miR5568c-5p | SORBI_3004G180900 | 4 | 412 | 432 | UCUGUUCCAAAUUGUAAGUCG | UGAUUUAUAAUUUGGGAUGAA | Cleavage | 1 |
| sbi-miR5568f-5p | SORBI_3004G180900 | 4 | 412 | 432 | UCCAUUCCAAAUUGUAAGAUG | UGAUUUAUAAUUUGGGAUGAA | Cleavage | 1 |
| sbi-miR6225-3p | SORBI_3003G315500 | 4 | 1714 | 1737 | GAAACGAAUCUUUUAAGUCUAAUU | AACUAGGCUUAAAAGAUUUAUCUC | Cleavage | 1 |
| sbi-miR6231-5p | SORBI_3005G191600 | 4 | 1135 | 1155 | GUCCGUGAGUCCACAAAUAGG | CGGAUUUGUGGAUUUCCGGGC | Cleavage | 1 |
| sbi-miR6232b-5p | SORBI_3008G082400 | 4 | 1666 | 1686 | UUUUUGGUACAUUGAAUUUGC | AAAAAUUAAAAGUACUGAAAG | Translation | 1 |
| sbi-miR821a | SORBI_3007G074200 | 4 | 1405 | 1425 | AAGUCAUCAACAUAAAAGUUG | CAACUUUUAUUUUGACGGUUU | Translation | 1 |
| sbi-miR821c | SORBI_3007G074200 | 4 | 1405 | 1425 | AAGUCAUCAACAUAAAAGUUG | CAACUUUUAUUUUGACGGUUU | Translation | 1 |
| sbi-miR156d | SORBI_3001G266100 | 4.5 | 1546 | 1566 | UGACAGAAGAGAGAGAGCACA | UGAGUUUUUUCUCUUUUUUCA | Cleavage | 1 |
| sbi-miR156d | SORBI_3005G191600 | 4.5 | 1012 | 1032 | UGACAGAAGAGAGAGAGCACA | AUUUCUCUUUCUCGUCUCUCA | Cleavage | 1 |
| sbi-miR159a | SORBI_3001G438500 | 4.5 | 511 | 531 | UUUGGAUUGAAGGGAGCUCUG | CAGAGUUUCUUUCGAGCCAAU | Cleavage | 1 |
| sbi-miR159b | SORBI_3001G438500 | 4.5 | 511 | 531 | CUUGGAUUGAAGGGAGCUCCU | CAGAGUUUCUUUCGAGCCAAU | Cleavage | 1 |
| sbi-miR437a | SORBI_3005G191600 | 4.5 | 1330 | 1350 | AAAGUUAGAGAAGUUUGACUU | AAUUUAGAUUUCUUUAAGUUU | Cleavage | 1 |
| sbi-miR437b | SORBI_3005G191600 | 4.5 | 1330 | 1350 | AAAGUUAGAGAAGUUUGACUU | AAUUUAGAUUUCUUUAAGUUU | Cleavage | 1 |
| sbi-miR437c | SORBI_3005G191600 | 4.5 | 1330 | 1350 | AAAGUUAGAGAAGUUUGACUU | AAUUUAGAUUUCUUUAAGUUU | Cleavage | 1 |
| sbi-miR437d | SORBI_3005G191600 | 4.5 | 1330 | 1350 | AAAGUUAGAGAAGUUUGACUU | AAUUUAGAUUUCUUUAAGUUU | Cleavage | 1 |
| sbi-miR437e | SORBI_3005G191600 | 4.5 | 1330 | 1350 | AAAGUUAGAGAAGUUUGACUU | AAUUUAGAUUUCUUUAAGUUU | Cleavage | 1 |
| sbi-miR437f | SORBI_3005G191600 | 4.5 | 1330 | 1350 | AAAGUUAGAGAAGUUUGACUU | AAUUUAGAUUUCUUUAAGUUU | Cleavage | 1 |
| sbi-miR437g | SORBI_3005G191600 | 4.5 | 1330 | 1350 | AAAGUUAGAGAAGUUUGACUU | AAUUUAGAUUUCUUUAAGUUU | Cleavage | 1 |
| sbi-miR437i | SORBI_3005G191600 | 4.5 | 1330 | 1350 | AAAGUUAGAGAAGUUUGACUU | AAUUUAGAUUUCUUUAAGUUU | Cleavage | 1 |
| sbi-miR437j | SORBI_3005G191600 | 4.5 | 1330 | 1350 | AAAGUUAGAGAAGUUUGACUU | AAUUUAGAUUUCUUUAAGUUU | Cleavage | 1 |
| sbi-miR437k | SORBI_3005G191600 | 4.5 | 1330 | 1350 | AAAGUUAGAGAAGUUUGACUU | AAUUUAGAUUUCUUUAAGUUU | Cleavage | 1 |
| sbi-miR437l | SORBI_3005G191600 | 4.5 | 1330 | 1350 | AAAGUUAGAGAAGUUUGACUU | AAUUUAGAUUUCUUUAAGUUU | Cleavage | 1 |
| sbi-miR437m | SORBI_3005G191600 | 4.5 | 1330 | 1350 | AAAGUUAGAGAAGUUUGACUU | AAUUUAGAUUUCUUUAAGUUU | Cleavage | 1 |
| sbi-miR437n | SORBI_3005G191600 | 4.5 | 1330 | 1350 | AAAGUUAGAGAAGUUUGACUU | AAUUUAGAUUUCUUUAAGUUU | Cleavage | 1 |
| sbi-miR437o | SORBI_3005G191600 | 4.5 | 1330 | 1350 | AAAGUUAGAGAAGUUUGACUU | AAUUUAGAUUUCUUUAAGUUU | Cleavage | 1 |
| sbi-miR437p | SORBI_3005G191600 | 4.5 | 1330 | 1350 | AAAGUUAGAGAAGUUUGACUU | AAUUUAGAUUUCUUUAAGUUU | Cleavage | 1 |
| sbi-miR437q | SORBI_3005G191600 | 4.5 | 1330 | 1350 | AAAGUUAGAGAAGUUUGACUU | AAUUUAGAUUUCUUUAAGUUU | Cleavage | 1 |
| sbi-miR437r | SORBI_3005G191600 | 4.5 | 1330 | 1350 | AAAGUUAGAGAAGUUUGACUU | AAUUUAGAUUUCUUUAAGUUU | Cleavage | 1 |
| sbi-miR437s | SORBI_3005G191600 | 4.5 | 1330 | 1350 | AAAGUUAGAGAAGUUUGACUU | AAUUUAGAUUUCUUUAAGUUU | Cleavage | 1 |
| sbi-miR437t | SORBI_3005G191600 | 4.5 | 1330 | 1350 | AAAGUUAGAGAAGUUUGACUU | AAUUUAGAUUUCUUUAAGUUU | Cleavage | 1 |
| sbi-miR437u | SORBI_3005G191600 | 4.5 | 1330 | 1350 | AAAGUUAGAGAAGUUUGACUU | AAUUUAGAUUUCUUUAAGUUU | Cleavage | 1 |
| sbi-miR437v | SORBI_3005G191600 | 4.5 | 1330 | 1350 | AAAGUUAGAGAAGUUUGACUU | AAUUUAGAUUUCUUUAAGUUU | Cleavage | 1 |
| sbi-miR437w | SORBI_3005G191600 | 4.5 | 1330 | 1350 | AAAGUUAGAGAAGUUUGACUU | AAUUUAGAUUUCUUUAAGUUU | Cleavage | 1 |
| sbi-miR5568c-3p | SORBI_3010G054600 | 4.5 | 1757 | 1777 | ACUUACAGUUUGGAACGGAGG | UCUCUAUUCCAAAUUAUAAGG | Cleavage | 1 |
| sbi-miR5568c-5p | SORBI_3007G074200 | 4.5 | 222 | 242 | UCUGUUCCAAAUUGUAAGUCG | UUACAGAUAUUUUGGGACAGA | Cleavage | 1 |
| sbi-miR5568e-3p | SORBI_3003G336300 | 4.5 | 1931 | 1951 | UAUCUAGAAAAGCUAAAACGU | AAGUUUCAGGUUUUUAAGAUA | Cleavage | 1 |
| sbi-miR5568f-3p | SORBI_3004G060600 | 4.5 | 1463 | 1483 | GUCUUAUAAUUUGGAAUGGAG | AAUUAUACUGAAAUAUAAGAC | Cleavage | 1 |
| sbi-miR5568g-5p | SORBI_3004G180900 | 4.5 | 405 | 425 | CAAAUUAUAAGAUGUUUUGGC | CUUGGAAUGAUUUAUAAUUUG | Cleavage | 1 |
| sbi-miR5568g-5p | SORBI_3006G238000 | 4.5 | 1250 | 1270 | CAAAUUAUAAGAUGUUUUGGC | AACAGAGCAUUUUUUAAUCUG | Cleavage | 1 |
| sbi-miR6217a-5p | SORBI_3007G074200 | 4.5 | 40 | 63 | UAGCCACUUUGAGUUACGAUAAUU | CUAUAUUGUAACCCAAAGUGCUUG | Cleavage | 1 |
| sbi-miR6217b-5p | SORBI_3007G074200 | 4.5 | 40 | 63 | UAGCCACUUUGAGUUACGAUAAUU | CUAUAUUGUAACCCAAAGUGCUUG | Cleavage | 1 |
| sbi-miR6218-3p | SORBI_3003G195000 | 4.5 | 794 | 814 | ACAAGUUUCGUGAUUUUUGGA | AAUAAAAAUCAUGAUAUGUGU | Cleavage | 1 |
| sbi-miR6220-5p | SORBI_3004G060600 | 4.5 | 1625 | 1648 | CUCCAUCCUAAAUUAUAAGACAUU | AAUAUAUUGUAUUAUAGGAUGGAG | Translation | 1 |
| sbi-miR6225-5p | SORBI_3007G074200 | 4.5 | 510 | 533 | AACUAGACUCAAAAGAUUCAUCUC | GAGACGAAUAUUUUGAGCUUAAUU | Cleavage | 1 |
| sbi-miR6228-5p | SORBI_3001G265900 | 4.5 | 1829 | 1852 | UUCUAUCUCUAUUAAUUGUGUUGC | AAGCAAAAAUAAAAAGGGAUAGAG | Translation | 1 |
| sbi-miR6230-5p | SORBI_3001G265900 | 4.5 | 212 | 232 | UUUUGGGUCCCUAAACUUGUU | UUUAAUUCUAGGGCUCCAAAA | Cleavage | 1 |
| sbi-miR6232b-5p | SORBI_3003G315500 | 4.5 | 1807 | 1827 | UUUUUGGUACAUUGAAUUUGC | CAAGAUUCGAUGUGAUGAGAA | Cleavage | 1 |
| sbi-miR6233-3p | SORBI_3010G278800 | 4.5 | 1090 | 1113 | CAAGUUUGGUUUUGGUAAUUAAUG | AAAGAAUUAUUAAAAUCAGAGUUA | Cleavage | 1 |
| sbi-miR6233-3p | SORBI_3001G438400 | 4.5 | 1903 | 1926 | CAAGUUUGGUUUUGGUAAUUAAUG | CAGUCAUGACAAAAAGCAAACUUU | Cleavage | 1 |
| sbi-miR156d | SORBI_3003G336300 | 5 | 1649 | 1669 | UGACAGAAGAGAGAGAGCACA | CUAUCUCUCUCCCUUCUUUCA | Translation | 1 |
| sbi-miR169o | SORBI_3004G060600 | 5 | 189 | 209 | UAGCCAAGGAUGAUUUGCCUG | AUAGCAAAUCCUCCUUUGCUC | Translation | 1 |
| sbi-miR171a | SORBI_3001G266100 | 5 | 29 | 49 | UGAUUGAGCCGUGCCAAUAUC | AUUAUAGGCAAAGCUUAAUUA | Translation | 1 |
| sbi-miR171b | SORBI_3001G266100 | 5 | 29 | 49 | UGAUUGAGCCGUGCCAAUAUC | AUUAUAGGCAAAGCUUAAUUA | Translation | 1 |
| sbi-miR171d | SORBI_3001G266100 | 5 | 29 | 49 | UGAUUGAGCCGUGCCAAUAUC | AUUAUAGGCAAAGCUUAAUUA | Translation | 1 |
| sbi-miR171i | SORBI_3001G266100 | 5 | 29 | 49 | UGAUUGAGCCGUGCCAAUAUC | AUUAUAGGCAAAGCUUAAUUA | Translation | 1 |
| sbi-miR171k | SORBI_3001G266100 | 5 | 29 | 49 | UGAUUGAGCCGUGCCAAUAUC | AUUAUAGGCAAAGCUUAAUUA | Translation | 1 |
| sbi-miR2118-3p | SORBI_3005G197700 | 5 | 667 | 688 | UUCCUGAUGCCUCCCAUGCCUA | CAAGCGUGGGGGGCAACCGGAU | Cleavage | 1 |
| sbi-miR2118-5p | SORBI_3003G336300 | 5 | 1634 | 1655 | GGCAUGGGAACAUGUAGGAAGG | GUGUUCUUUGUGUUCCUAUCUC | Cleavage | 1 |
| sbi-miR394a | SORBI_3005G197700 | 5 | 180 | 199 | UUGGCAUUCUGUCCACCUCC | AGAGGCGGGCAGGAUUCCCA | Cleavage | 1 |
| sbi-miR394b | SORBI_3005G197700 | 5 | 180 | 199 | UUGGCAUUCUGUCCACCUCC | AGAGGCGGGCAGGAUUCCCA | Cleavage | 1 |
| sbi-miR395k | SORBI_3003G336300 | 5 | 2006 | 2029 | GUGAAGUGUUUG---GAGGAACUC | AGGUUCCUCGUACAAACGCUUCAC | Cleavage | 1 |
| sbi-miR437a | SORBI_3010G054600 | 5 | 1906 | 1926 | AAAGUUAGAGAAGUUUGACUU | GUUACAAAUUUUUAUAAUUUU | Cleavage | 1 |
| sbi-miR437b | SORBI_3010G054600 | 5 | 1906 | 1926 | AAAGUUAGAGAAGUUUGACUU | GUUACAAAUUUUUAUAAUUUU | Cleavage | 1 |
| sbi-miR437c | SORBI_3010G054600 | 5 | 1906 | 1926 | AAAGUUAGAGAAGUUUGACUU | GUUACAAAUUUUUAUAAUUUU | Cleavage | 1 |
| sbi-miR437d | SORBI_3010G054600 | 5 | 1906 | 1926 | AAAGUUAGAGAAGUUUGACUU | GUUACAAAUUUUUAUAAUUUU | Cleavage | 1 |
| sbi-miR437e | SORBI_3010G054600 | 5 | 1906 | 1926 | AAAGUUAGAGAAGUUUGACUU | GUUACAAAUUUUUAUAAUUUU | Cleavage | 1 |
| sbi-miR437f | SORBI_3010G054600 | 5 | 1906 | 1926 | AAAGUUAGAGAAGUUUGACUU | GUUACAAAUUUUUAUAAUUUU | Cleavage | 1 |
| sbi-miR437g | SORBI_3010G054600 | 5 | 1906 | 1926 | AAAGUUAGAGAAGUUUGACUU | GUUACAAAUUUUUAUAAUUUU | Cleavage | 1 |
| sbi-miR437i | SORBI_3010G054600 | 5 | 1906 | 1926 | AAAGUUAGAGAAGUUUGACUU | GUUACAAAUUUUUAUAAUUUU | Cleavage | 1 |
| sbi-miR437j | SORBI_3010G054600 | 5 | 1906 | 1926 | AAAGUUAGAGAAGUUUGACUU | GUUACAAAUUUUUAUAAUUUU | Cleavage | 1 |
| sbi-miR437k | SORBI_3010G054600 | 5 | 1906 | 1926 | AAAGUUAGAGAAGUUUGACUU | GUUACAAAUUUUUAUAAUUUU | Cleavage | 1 |
| sbi-miR437l | SORBI_3010G054600 | 5 | 1906 | 1926 | AAAGUUAGAGAAGUUUGACUU | GUUACAAAUUUUUAUAAUUUU | Cleavage | 1 |
| sbi-miR437m | SORBI_3010G054600 | 5 | 1906 | 1926 | AAAGUUAGAGAAGUUUGACUU | GUUACAAAUUUUUAUAAUUUU | Cleavage | 1 |
| sbi-miR437n | SORBI_3010G054600 | 5 | 1906 | 1926 | AAAGUUAGAGAAGUUUGACUU | GUUACAAAUUUUUAUAAUUUU | Cleavage | 1 |
| sbi-miR437o | SORBI_3010G054600 | 5 | 1906 | 1926 | AAAGUUAGAGAAGUUUGACUU | GUUACAAAUUUUUAUAAUUUU | Cleavage | 1 |
| sbi-miR437p | SORBI_3010G054600 | 5 | 1906 | 1926 | AAAGUUAGAGAAGUUUGACUU | GUUACAAAUUUUUAUAAUUUU | Cleavage | 1 |
| sbi-miR437q | SORBI_3010G054600 | 5 | 1906 | 1926 | AAAGUUAGAGAAGUUUGACUU | GUUACAAAUUUUUAUAAUUUU | Cleavage | 1 |
| sbi-miR437r | SORBI_3010G054600 | 5 | 1906 | 1926 | AAAGUUAGAGAAGUUUGACUU | GUUACAAAUUUUUAUAAUUUU | Cleavage | 1 |
| sbi-miR437s | SORBI_3010G054600 | 5 | 1906 | 1926 | AAAGUUAGAGAAGUUUGACUU | GUUACAAAUUUUUAUAAUUUU | Cleavage | 1 |
| sbi-miR437t | SORBI_3010G054600 | 5 | 1906 | 1926 | AAAGUUAGAGAAGUUUGACUU | GUUACAAAUUUUUAUAAUUUU | Cleavage | 1 |
| sbi-miR437u | SORBI_3010G054600 | 5 | 1906 | 1926 | AAAGUUAGAGAAGUUUGACUU | GUUACAAAUUUUUAUAAUUUU | Cleavage | 1 |
| sbi-miR437v | SORBI_3010G054600 | 5 | 1906 | 1926 | AAAGUUAGAGAAGUUUGACUU | GUUACAAAUUUUUAUAAUUUU | Cleavage | 1 |
| sbi-miR437w | SORBI_3010G054600 | 5 | 1906 | 1926 | AAAGUUAGAGAAGUUUGACUU | GUUACAAAUUUUUAUAAUUUU | Cleavage | 1 |
| sbi-miR529 | SORBI_3001G266100 | 5 | 840 | 859 | CUGUACCCUCUCUCUUCUUC | AGAGGGGAGAUAGGGUAAGG | Translation | 1 |
| sbi-miR5388 | SORBI_3010G054600 | 5 | 1483 | 1504 | AUCUUUGCCGGGUGUCUCUGAC | AGCAGAAACACACGGCAAUGAA | Translation | 1 |
| sbi-miR5565d | SORBI_3001G266200 | 5 | 1786 | 1808 | ACUUCAAUCCAUGUAUGUUGGUGU | GGACGGACA-GCAUGGAUUCGAGU | Cleavage | 1 |
| sbi-miR5566 | SORBI_3006G211701 | 5 | 362 | 382 | UCAGCAUCACCUCCCUGUUGU | ACAACGGGAGGGUGGUGUUAA | Cleavage | 1 |
| sbi-miR5568c-3p | SORBI_3003G336300 | 5 | 1871 | 1891 | ACUUACAGUUUGGAACGGAGG | GUACCGGCCCAAACUGUCGGU | Cleavage | 1 |
| sbi-miR5568d-3p | SORBI_3007G074200 | 5 | 1243 | 1263 | AAAGUUGUGUAUCUAGAAAAG | CUUUUUAAGACCUACAACUUU | Translation | 2 |
| sbi-miR5568d-3p | SORBI_3001G266200 | 5 | 588 | 608 | AAAGUUGUGUAUCUAGAAAAG | UAUUCAUAGAAACACAAUUUG | Translation | 1 |
| sbi-miR5568e-5p | SORBI_3006G031100 | 5 | 1404 | 1424 | GAUGUUUUGGGUUUUCUAGAU | GAAUAAAAAAUCCAAAUCAUG | Cleavage | 1 |
| sbi-miR5568e-5p | SORBI_3001G438400 | 5 | 837 | 857 | GAUGUUUUGGGUUUUCUAGAU | GCCCACGAAAUUCAAACCAUC | Cleavage | 1 |
| sbi-miR5568g-5p | SORBI_3010G054600 | 5 | 1791 | 1810 | CAAAUUAUAAGAUGUUUUGGC | GUUAAAAUAUUUUA-AGUUUG | Cleavage | 1 |
| sbi-miR5568g-5p | SORBI_3007G074200 | 5 | 404 | 424 | CAAAUUAUAAGAUGUUUUGGC | UACAAAACUUUUUACGAUUUU | Cleavage | 1 |
| sbi-miR5568g-5p | SORBI_3001G266200 | 5 | 1417 | 1437 | CAAAUUAUAAGAUGUUUUGGC | UUGAUAUUAUUUUGUAGUUUG | Cleavage | 1 |
| sbi-miR6218-3p | SORBI_3010G278800 | 5 | 1032 | 1052 | ACAAGUUUCGUGAUUUUUGGA | UCCAAAAAUCAUGAAAUUAAU | Cleavage | 1 |
| sbi-miR6218-3p | SORBI_3007G074200 | 5 | 962 | 982 | ACAAGUUUCGUGAUUUUUGGA | UCCAGAAAUUAUGAAAUCAGU | Cleavage | 1 |
| sbi-miR6219-5p | SORBI_3010G054600 | 5 | 699 | 722 | GAACCGGGACUAAAGGUGGGACAU | CCAUGCCACAUUUAGUCAUUGUUU | Cleavage | 1 |
| sbi-miR6224a-5p | SORBI_3004G060600 | 5 | 1628 | 1648 | CUCCGUCCUAAUAUAUAAGGC | AUAUUGUAUUAUAGGAUGGAG | Translation | 2 |
| sbi-miR6224a-5p | SORBI_3004G060600 | 5 | 1839 | 1859 | CUCCGUCCUAAUAUAUAAGGC | GGGGUAUUUAUUAGGGGGGAG | Cleavage | 2 |
| sbi-miR6224b-5p | SORBI_3004G060600 | 5 | 1628 | 1648 | CUCCGUCCUAAUAUAUAAGGC | AUAUUGUAUUAUAGGAUGGAG | Translation | 2 |
| sbi-miR6224b-5p | SORBI_3004G060600 | 5 | 1839 | 1859 | CUCCGUCCUAAUAUAUAAGGC | GGGGUAUUUAUUAGGGGGGAG | Cleavage | 2 |
| sbi-miR6224c-5p | SORBI_3004G060600 | 5 | 1628 | 1648 | CUCCGUCCUAAUAUAUAAGGC | AUAUUGUAUUAUAGGAUGGAG | Translation | 2 |
| sbi-miR6224c-5p | SORBI_3004G060600 | 5 | 1839 | 1859 | CUCCGUCCUAAUAUAUAAGGC | GGGGUAUUUAUUAGGGGGGAG | Cleavage | 2 |
| sbi-miR6225-3p | SORBI_3003G336300 | 5 | 293 | 316 | GAAACGAAUCUUUUAAGUCUAAUU | AACUAGGACCAAAAGAUUCGUCUC | Cleavage | 1 |
| sbi-miR6228-5p | SORBI_3004G060600 | 5 | 1991 | 2014 | UUCUAUCUCUAUUAAUUGUGUUGC | ACGUGAUUAUUGAUAGAGGGAGAU | Cleavage | 1 |
| sbi-miR6229-3p | SORBI_3004G180900 | 5 | 71 | 94 | GUUUUUCUCGCCGGGUGAGAAGGC | AUGCUUUAACUCGGGCAGAAAAAC | Translation | 1 |
| sbi-miR6232a-3p | SORBI_3005G191600 | 5 | 1817 | 1840 | UGGAUGUACCAAAAAAGUCAAAGC | UGUGUGAUUUUUUUGAUGAAUUUA | Cleavage | 1 |
| sbi-miR6232b-3p | SORBI_3007G074200 | 5 | 419 | 439 | AAUUCGAUGUACCAAAAAAGU | GAUUUUCUGUCACAUCGAAUC | Translation | 1 |
| sbi-miR6232b-5p | SORBI_3010G054600 | 5 | 884 | 904 | UUUUUGGUACAUUGAAUUUGC | CAAGAUUUGAUGUGAUGAGAA | Cleavage | 1 |
| sbi-miR6233-3p | SORBI_3001G266100 | 5 | 41 | 63 | CAAGUUUGGUUUUGGUAAUUAAUG | GCUUAAUUAU-AAAGCUAAGCUUA | Cleavage | 1 |
| sbi-miR6233-3p | SORBI_3006G211701 | 5 | 294 | 317 | CAAGUUUGGUUUUGGUAAUUAAUG | CCGUAAUUAGUAAAACUAACCAUG | Cleavage | 1 |
| sbi-miR821a | SORBI_3004G060600 | 5 | 214 | 234 | AAGUCAUCAACAUAAAAGUUG | CAACUUUAGUGGUGAUUAUUU | Translation | 1 |
| sbi-miR821b | SORBI_3010G054600 | 5 | 1976 | 1996 | AAGUUAUGAACAUAAAAGUUG | AUAUUUUUAUAUUCUUAAUUA | Translation | 1 |
| sbi-miR821c | SORBI_3004G060600 | 5 | 214 | 234 | AAGUCAUCAACAUAAAAGUUG | CAACUUUAGUGGUGAUUAUUU | Translation | 1 |
