# Supplementary material for: Identification and analysis of proline-rich proteins and hybrid proline-rich proteins super family genes from Sorghum bicolor and their expression patterns to abiotic stress and zinc stimuli
Source: Front Plant Sci. 2022 Sep 26;13:952732. doi: 10.3389/fpls.2022.952732 (PMC9549341; doi:10.3389/fpls.2022.952732)
Supplement: Supplementary file 24 [file Table_12.doc]

**Table S12.** miRNAs that target *SbHyPRP* genes

| miRNA_Acc. | Target_Acc. | Expectation | Target_start | Target_end | miRNA_aligned_fragment | Target_aligned_fragment | Inhibition | Mmultiplicity | |
| --- | --- | --- | --- | --- | --- | --- | --- | --- | --- |
| sbi-miR5565f | SORBI_3006G172550 | 1 | 1584 | 1603 | UAGUCGGAUUUAUAUCAAUC | GAUUGAUGUGAAUCCGACUA | Cleavage | 1 |  |
| sbi-miR5565g-5p | SORBI_3006G172550 | 1 | 1572 | 1595 | UUCACAUCAAUCCACAUAUGUUGG | UCAACACAUGUGGAUUGAUGUGAA | Cleavage | 2 |  |
| sbi-miR5565e | SORBI_3006G172550 | 1.5 | 1596 | 1614 | UUGUUUGGAUGUUGUCGGA | UCCGACUACAUCCAAACAA | Cleavage | 1 |  |
| sbi-miR6227-5p | SORBI_3010G003600 | 2 | 1851 | 1874 | GGGCCCAAAUAGCAAGUGUUGUGA | UCACAACACUUGUUAUUUGGACCC | Cleavage | 1 |  |
| sbi-miR5565a | SORBI_3006G172550 | 2.5 | 1501 | 1524 | AACACAUGUGGAUUGAGGCGAAUC | GAUUCACUUCAAUCCAUGUGUGUU | Cleavage | 1 |  |
| sbi-miR5565b | SORBI_3006G172550 | 2.5 | 1501 | 1524 | AACACAUGUGGAUUGAGGCGAAUC | GAUUCACUUCAAUCCAUGUGUGUU | Cleavage | 1 |  |
| sbi-miR5565c | SORBI_3006G172550 | 2.5 | 1504 | 1524 | UACACAUGUGGAUUGAGGUGA | UCACUUCAAUCCAUGUGUGUU | Cleavage | 1 |  |
| sbi-miR5565g-3p | SORBI_3006G172550 | 2.5 | 1500 | 1523 | ACACAUGUGGAUUGAGAUGAAUAC | GGAUUCACUUCAAUCCAUGUGUGU | Cleavage | 1 |  |
| sbi-miR395a | SORBI_3003G329400 | 3 | 1146 | 1166 | GUGAAGUGUUUGGGGGAACUC | CAGUUUCCUUAAAUAUUUUAC | Cleavage | 1 |  |
| sbi-miR395b | SORBI_3003G329400 | 3 | 1146 | 1166 | GUGAAGUGUUUGGGGGAACUC | CAGUUUCCUUAAAUAUUUUAC | Cleavage | 1 |  |
| sbi-miR395c | SORBI_3003G329400 | 3 | 1146 | 1166 | GUGAAGUGUUUGGGGGAACUC | CAGUUUCCUUAAAUAUUUUAC | Cleavage | 1 |  |
| sbi-miR395d | SORBI_3003G329400 | 3 | 1146 | 1166 | GUGAAGUGUUUGGGGGAACUC | CAGUUUCCUUAAAUAUUUUAC | Cleavage | 1 |  |
| sbi-miR395e | SORBI_3003G329400 | 3 | 1146 | 1166 | GUGAAGUGUUUGGGGGAACUC | CAGUUUCCUUAAAUAUUUUAC | Cleavage | 1 |  |
| sbi-miR395g | SORBI_3003G329400 | 3 | 1146 | 1166 | GUGAAGUGUUUGGGGGAACUC | CAGUUUCCUUAAAUAUUUUAC | Cleavage | 1 |  |
| sbi-miR395h | SORBI_3003G329400 | 3 | 1146 | 1166 | GUGAAGUGUUUGGGGGAACUC | CAGUUUCCUUAAAUAUUUUAC | Cleavage | 1 |  |
| sbi-miR395i | SORBI_3003G329400 | 3 | 1146 | 1166 | GUGAAGUGUUUGGGGGAACUC | CAGUUUCCUUAAAUAUUUUAC | Cleavage | 1 |  |
| sbi-miR395j | SORBI_3003G329400 | 3 | 1146 | 1166 | GUGAAGUGUUUGGGGGAACUC | CAGUUUCCUUAAAUAUUUUAC | Cleavage | 1 |  |
| sbi-miR5389 | SORBI_3001G304201 | 3 | 766 | 786 | GCUUGAGUUUAUCAGCCGAGU | AUUCGACAGAUAAAUUUAAGC | Cleavage | 1 |  |
| sbi-miR5565g-5p | SORBI_3006G172550 | 3 | 1519 | 1542 | UUCACAUCAAUCCACAUAUGUUGG | UGUGUUGGUGUGGAUUGGUGUGAA | Cleavage | 2 |  |
| sbi-miR5568c-5p | SORBI_3001G304300 | 3 | 1410 | 1430 | UCUGUUCCAAAUUGUAAGUCG | AAAUUUAUAGUUUGGAACAAA | Cleavage | 1 |  |
| sbi-miR5568e-3p | SORBI_3001G304300 | 3 | 841 | 861 | UAUCUAGAAAAGCUAAAACGU | UUAUUUUAGUUUUUCUACAUA | Cleavage | 2 |  |
| sbi-miR528 | SORBI_3003G149800 | 3.5 | 416 | 436 | UGGAAGGGGCAUGCAGAGGAG | AUUUUCUUCAUGCCUCUUCCU | Cleavage | 1 |  |
| sbi-miR5565d | SORBI_3006G172550 | 3.5 | 1569 | 1592 | ACUUCAAUCCAUGUAUGUUGGUGU | ACCUCAACACAUGUGGAUUGAUGU | Cleavage | 1 |  |
| sbi-miR5567 | SORBI_3001G542000 | 3.5 | 1203 | 1226 | UUAAUGAUUCAUGUAUGUGUCCAA | UACGACACAUACAUAAAAUAUUAA | 1 |  | |
| sbi-miR5568e-3p | SORBI_3001G304300 | 3.5 | 1078 | 1098 | UAUCUAGAAAAGCUAAAACGU | UCGUUUUAGUUUUUCCACAUA | Cleavage | 2 |  |
| sbi-miR164a | SORBI_3003G126100 | 4 | 739 | 759 | UGGAGAAGCAGGGCACGUGCA | CGCCCUUGCUCUGCUUCUCGA | Cleavage | 1 |  |
| sbi-miR164b | SORBI_3003G126100 | 4 | 739 | 759 | UGGAGAAGCAGGGCACGUGCU | CGCCCUUGCUCUGCUUCUCGA | Cleavage | 1 |  |
| sbi-miR164d | SORBI_3003G126100 | 4 | 739 | 759 | UGGAGAAGCAGGGCACGUGCA | CGCCCUUGCUCUGCUUCUCGA | Cleavage | 1 |  |
| sbi-miR164e | SORBI_3003G126100 | 4 | 739 | 759 | UGGAGAAGCAGGGCACGUGCA | CGCCCUUGCUCUGCUUCUCGA | Cleavage | 1 |  |
| sbi-miR395f | SORBI_3003G329400 | 4 | 1146 | 1166 | AUGAAGUGUUUGGGGGAACUC | CAGUUUCCUUAAAUAUUUUAC | Cleavage | 1 |  |
| sbi-miR395f | SORBI_3004G292001 | 4 | 1636 | 1656 | AUGAAGUGUUUGGGGGAACUC | UUGUAUCUCCAAAUAUUUUGU | Cleavage | 1 |  |
| sbi-miR395k | SORBI_3003G329400 | 4 | 1146 | 1166 | GUGAAGUGUUUGGAGGAACUC | CAGUUUCCUUAAAUAUUUUAC | Cleavage | 1 |  |
| sbi-miR395k | SORBI_3004G292001 | 4 | 1636 | 1656 | GUGAAGUGUUUGGAGGAACUC | UUGUAUCUCCAAAUAUUUUGU | Cleavage | 1 |  |
| sbi-miR5388 | SORBI_3004G292900 | 4 | 209 | 230 | AUCUUUGCCGGGUGUCUCUGAC | UUUUUAGGCACUCGGCAAAGAA | Cleavage | 2 |  |
| sbi-miR5564c-3p | SORBI_3004G292250 | 4 | 1056 | 1076 | ACGCGAGCUGUUUGGCGAAUU | UCGUCGUCGAACGGCUCGUGC | Cleavage | 1 |  |
| sbi-miR5565a | SORBI_3003G329400 | 4 | 1283 | 1306 | AACACAUGUGGAUUGAGGCGAAUC | CCAAUGCUACAAAUUACAUGUGUU | Cleavage | 1 |  |
| sbi-miR5565b | SORBI_3003G329400 | 4 | 1283 | 1306 | AACACAUGUGGAUUGAGGCGAAUC | CCAAUGCUACAAAUUACAUGUGUU | Cleavage | 1 |  |
| sbi-miR5568g-5p | SORBI_3004G291500 | 4 | 1425 | 1445 | CAAAUUAUAAGAUGUUUUGGC | UGCAGUGCCUCUUGUAAUUUG | Cleavage | 1 |  |
| sbi-miR5569 | SORBI_3004G292001 | 4 | 1695 | 1718 | UAUUGCAUGCUUGAACUAUGGUAA | UUACUGUAGUUCAAGUAUACAUUA | Cleavage | 1 |  |
| sbi-miR821e | SORBI_3004G292900 | 4 | 406 | 426 | AAGUCAUCAAAAUAAAAGUUG | CAAUGUUUAUUUUGGUAAUUU | Cleavage | 1 |  |
| sbi-miR1435b | SORBI_3003G058900 | 4.5 | 85 | 104 | UUUCUUAAGUCAAACCUUUU | CUAGGGUUUCAUUUAGGAAG | 1 |  | |
| sbi-miR156d | SORBI_3001G304201 | 4.5 | 433 | 453 | UGACAGAAGAGAGAGAGCACA | UUAGCUUUUUUUUUUUUGUUG | Cleavage | 1 |  |
| sbi-miR160a | SORBI_3005G046700 | 4.5 | 1258 | 1278 | UGCCUGGCUCCCUGUAUGCCA | AAAAAUGCAAGGAGCCAGGUA | Cleavage | 1 |  |
| sbi-miR160b | SORBI_3005G046700 | 4.5 | 1258 | 1278 | UGCCUGGCUCCCUGUAUGCCA | AAAAAUGCAAGGAGCCAGGUA | Cleavage | 1 |  |
| sbi-miR160c | SORBI_3005G046700 | 4.5 | 1258 | 1278 | UGCCUGGCUCCCUGUAUGCCA | AAAAAUGCAAGGAGCCAGGUA | Cleavage | 1 |  |
| sbi-miR160d | SORBI_3005G046700 | 4.5 | 1258 | 1278 | UGCCUGGCUCCCUGUAUGCCA | AAAAAUGCAAGGAGCCAGGUA | Cleavage | 1 |  |
| sbi-miR160e | SORBI_3005G046700 | 4.5 | 1258 | 1278 | UGCCUGGCUCCCUGUAUGCCA | AAAAAUGCAAGGAGCCAGGUA | Cleavage | 1 |  |
| sbi-miR395a | SORBI_3004G292001 | 4.5 | 1636 | 1656 | GUGAAGUGUUUGGGGGAACUC | UUGUAUCUCCAAAUAUUUUGU | Cleavage | 1 |  |
| sbi-miR395b | SORBI_3004G292001 | 4.5 | 1636 | 1656 | GUGAAGUGUUUGGGGGAACUC | UUGUAUCUCCAAAUAUUUUGU | Cleavage | 1 |  |
| sbi-miR395c | SORBI_3004G292001 | 4.5 | 1636 | 1656 | GUGAAGUGUUUGGGGGAACUC | UUGUAUCUCCAAAUAUUUUGU | Cleavage | 1 |  |
| sbi-miR395d | SORBI_3004G292001 | 4.5 | 1636 | 1656 | GUGAAGUGUUUGGGGGAACUC | UUGUAUCUCCAAAUAUUUUGU | Cleavage | 1 |  |
| sbi-miR395e | SORBI_3004G292001 | 4.5 | 1636 | 1656 | GUGAAGUGUUUGGGGGAACUC | UUGUAUCUCCAAAUAUUUUGU | Cleavage | 1 |  |
| sbi-miR395g | SORBI_3004G292001 | 4.5 | 1636 | 1656 | GUGAAGUGUUUGGGGGAACUC | UUGUAUCUCCAAAUAUUUUGU | Cleavage | 1 |  |
| sbi-miR395h | SORBI_3004G292001 | 4.5 | 1636 | 1656 | GUGAAGUGUUUGGGGGAACUC | UUGUAUCUCCAAAUAUUUUGU | Cleavage | 1 |  |
| sbi-miR395i | SORBI_3004G292001 | 4.5 | 1636 | 1656 | GUGAAGUGUUUGGGGGAACUC | UUGUAUCUCCAAAUAUUUUGU | Cleavage | 1 |  |
| sbi-miR395j | SORBI_3004G292001 | 4.5 | 1636 | 1656 | GUGAAGUGUUUGGGGGAACUC | UUGUAUCUCCAAAUAUUUUGU | Cleavage | 1 |  |
| sbi-miR395l | SORBI_3003G329400 | 4.5 | 1146 | 1166 | GUGAAGUGCUUGGGGGAACUC | CAGUUUCCUUAAAUAUUUUAC | Cleavage | 1 |  |
| sbi-miR399a | SORBI_3001G112200 | 4.5 | 1549 | 1569 | UGCCAAAGGAGAAUUGCCCUG | UUGGGGAAUUUUCUUUUGACC | Cleavage | 1 |  |
| sbi-miR399c | SORBI_3001G112200 | 4.5 | 1549 | 1569 | UGCCAAAGGAGAAUUGCCCUG | UUGGGGAAUUUUCUUUUGACC | Cleavage | 1 |  |
| sbi-miR399h | SORBI_3001G112200 | 4.5 | 1549 | 1569 | UGCCAAAGGAGAAUUGCCCUG | UUGGGGAAUUUUCUUUUGACC | Cleavage | 1 |  |
| sbi-miR399j | SORBI_3001G112200 | 4.5 | 1549 | 1569 | UGCCAAAGGAGAAUUGCCCUG | UUGGGGAAUUUUCUUUUGACC | Cleavage | 1 |  |
| sbi-miR437a | SORBI_3001G302600 | 4.5 | 1237 | 1257 | AAAGUUAGAGAAGUUUGACUU | AAGUUAAAUUUUUAAAACUUU | Cleavage | 1 |  |
| sbi-miR437b | SORBI_3001G302600 | 4.5 | 1237 | 1257 | AAAGUUAGAGAAGUUUGACUU | AAGUUAAAUUUUUAAAACUUU | Cleavage | 1 |  |
| sbi-miR437c | SORBI_3001G302600 | 4.5 | 1237 | 1257 | AAAGUUAGAGAAGUUUGACUU | AAGUUAAAUUUUUAAAACUUU | Cleavage | 1 |  |
| sbi-miR437d | SORBI_3001G302600 | 4.5 | 1237 | 1257 | AAAGUUAGAGAAGUUUGACUU | AAGUUAAAUUUUUAAAACUUU | Cleavage | 1 |  |
| sbi-miR437e | SORBI_3001G302600 | 4.5 | 1237 | 1257 | AAAGUUAGAGAAGUUUGACUU | AAGUUAAAUUUUUAAAACUUU | Cleavage | 1 |  |
| sbi-miR437f | SORBI_3001G302600 | 4.5 | 1237 | 1257 | AAAGUUAGAGAAGUUUGACUU | AAGUUAAAUUUUUAAAACUUU | Cleavage | 1 |  |
| sbi-miR437g | SORBI_3001G302600 | 4.5 | 1237 | 1257 | AAAGUUAGAGAAGUUUGACUU | AAGUUAAAUUUUUAAAACUUU | Cleavage | 1 |  |
| sbi-miR437i | SORBI_3001G302600 | 4.5 | 1237 | 1257 | AAAGUUAGAGAAGUUUGACUU | AAGUUAAAUUUUUAAAACUUU | Cleavage | 1 |  |
| sbi-miR437j | SORBI_3001G302600 | 4.5 | 1237 | 1257 | AAAGUUAGAGAAGUUUGACUU | AAGUUAAAUUUUUAAAACUUU | Cleavage | 1 |  |
| sbi-miR437k | SORBI_3001G302600 | 4.5 | 1237 | 1257 | AAAGUUAGAGAAGUUUGACUU | AAGUUAAAUUUUUAAAACUUU | Cleavage | 1 |  |
| sbi-miR437l | SORBI_3001G302600 | 4.5 | 1237 | 1257 | AAAGUUAGAGAAGUUUGACUU | AAGUUAAAUUUUUAAAACUUU | Cleavage | 1 |  |
| sbi-miR437m | SORBI_3001G302600 | 4.5 | 1237 | 1257 | AAAGUUAGAGAAGUUUGACUU | AAGUUAAAUUUUUAAAACUUU | Cleavage | 1 |  |
| sbi-miR437n | SORBI_3001G302600 | 4.5 | 1237 | 1257 | AAAGUUAGAGAAGUUUGACUU | AAGUUAAAUUUUUAAAACUUU | Cleavage | 1 |  |
| sbi-miR437o | SORBI_3001G302600 | 4.5 | 1237 | 1257 | AAAGUUAGAGAAGUUUGACUU | AAGUUAAAUUUUUAAAACUUU | Cleavage | 1 |  |
| sbi-miR437p | SORBI_3001G302600 | 4.5 | 1237 | 1257 | AAAGUUAGAGAAGUUUGACUU | AAGUUAAAUUUUUAAAACUUU | Cleavage | 1 |  |
| sbi-miR437q | SORBI_3001G302600 | 4.5 | 1237 | 1257 | AAAGUUAGAGAAGUUUGACUU | AAGUUAAAUUUUUAAAACUUU | Cleavage | 1 |  |
| sbi-miR437r | SORBI_3001G302600 | 4.5 | 1237 | 1257 | AAAGUUAGAGAAGUUUGACUU | AAGUUAAAUUUUUAAAACUUU | Cleavage | 1 |  |
| sbi-miR437s | SORBI_3001G302600 | 4.5 | 1237 | 1257 | AAAGUUAGAGAAGUUUGACUU | AAGUUAAAUUUUUAAAACUUU | Cleavage | 1 |  |
| sbi-miR437t | SORBI_3001G302600 | 4.5 | 1237 | 1257 | AAAGUUAGAGAAGUUUGACUU | AAGUUAAAUUUUUAAAACUUU | Cleavage | 1 |  |
| sbi-miR437u | SORBI_3001G302600 | 4.5 | 1237 | 1257 | AAAGUUAGAGAAGUUUGACUU | AAGUUAAAUUUUUAAAACUUU | Cleavage | 1 |  |
| sbi-miR437v | SORBI_3001G302600 | 4.5 | 1237 | 1257 | AAAGUUAGAGAAGUUUGACUU | AAGUUAAAUUUUUAAAACUUU | Cleavage | 1 |  |
| sbi-miR437w | SORBI_3001G302600 | 4.5 | 1237 | 1257 | AAAGUUAGAGAAGUUUGACUU | AAGUUAAAUUUUUAAAACUUU | Cleavage | 1 |  |
| sbi-miR5385 | SORBI_3007G030100 | 4.5 | 1500 | 1521 | ACCACCAACCCCACCGCUUCUC | CGGUGGCGGUGGCGUUGGUCGU | 1 |  | |
| sbi-miR5565d | SORBI_3010G003600 | 4.5 | 531 | 554 | ACUUCAAUCCAUGUAUGUUGGUGU | UAUAAGACGAUUAUGGAUUGAAGA | Cleavage | 1 |  |
| sbi-miR5565f | SORBI_3006G238100 | 4.5 | 1045 | 1064 | UAGUCGGAUUUAUAUCAAUC | CCUGGAUAUGGAUUCGACUU | Cleavage | 1 |  |
| sbi-miR5567 | SORBI_3004G292001 | 4.5 | 1758 | 1781 | UUAAUGAUUCAUGUAUGUGUCCAA | GGAACUAUAUAUAUGAAUUAUUCG | Cleavage | 1 |  |
| sbi-miR5567 | SORBI_3001G304201 | 4.5 | 344 | 367 | UUAAUGAUUCAUGUAUGUGUCCAA | GUAAACACAUGUAUGUGUAAUUAA | Cleavage | 1 |  |
| sbi-miR5568b-3p | SORBI_3001G304300 | 4.5 | 847 | 867 | ACUAUGUAUCUAGAAAAGCUA | UAGUUUUUCUACAUACAUAUG | 2 |  | |
| sbi-miR5568b-3p | SORBI_3001G304300 | 4.5 | 1084 | 1104 | ACUAUGUAUCUAGAAAAGCUA | UAGUUUUUCCACAUACAUAGC | 2 |  | |
| sbi-miR5568d-3p | SORBI_3001G541300 | 4.5 | 281 | 301 | AAAGUUGUGUAUCUAGAAAAG | UUUUUCUAUAUAUAUAAUGUU | Cleavage | 1 |  |
| sbi-miR5568d-3p | SORBI_3001G304300 | 4.5 | 1087 | 1107 | AAAGUUGUGUAUCUAGAAAAG | UUUUUCCACAUACAUAGCUUC | Cleavage | 1 |  |
| sbi-miR5568g-5p | SORBI_3006G238100 | 4.5 | 219 | 239 | CAAAUUAUAAGAUGUUUUGGC | AACAGAGCAUUUUUUAAUCUG | Cleavage | 1 |  |
| sbi-miR5568g-5p | SORBI_3004G292900 | 4.5 | 1305 | 1325 | CAAAUUAUAAGAUGUUUUGGC | AACUAAAUAACUUAUAGUUUU | Cleavage | 1 |  |
| sbi-miR5568g-5p | SORBI_3001G304300 | 4.5 | 1403 | 1423 | CAAAUUAUAAGAUGUUUUGGC | AUAAAAAAAAUUUAUAGUUUG | Cleavage | 1 |  |
| sbi-miR6233-5p | SORBI_3006G172550 | 4.5 | 1002 | 1025 | UGUUGAGGCUGGAGCGAAACUCGG | GUGCGUUGCGUUUCAGCUGCAAUA | Cleavage | 1 |  |
| sbi-miR6235-3p | SORBI_3001G304201 | 4.5 | 702 | 725 | AACGAACAGUAUUUUUCUCUUACA | UCAUGACUGAAAGUACUGUUUGUU | Cleavage | 1 |  |
| sbi-miR6235-3p | SORBI_3004G292250 | 4.5 | 1854 | 1877 | AACGAACAGUAUUUUUCUCUUACA | AAGGUGAGAAUGAUACUAUUUGUC | Cleavage | 1 |  |
| sbi-miR6235-5p | SORBI_3001G473200 | 4.5 | 474 | 497 | UUGUGAGAGAAAAAUACUGUUGGC | ACAAACAAUGUUUUUCUCCUACAC | Cleavage | 1 |  |
| sbi-miR6235-5p | SORBI_3004G291500 | 4.5 | 1317 | 1339 | UUGUGAGAGAAAAAUACUGUUGGC | GUGACCAGU-UUUUUUUUUUGCAG | Cleavage | 1 |  |
| sbi-miR821b | SORBI_3003G149800 | 4.5 | 847 | 867 | AAGUUAUGAACAUAAAAGUUG | CGAUUUCUUUGUUCAUGACUA | Cleavage | 1 |  |
| sbi-miR1435a | SORBI_3003G058900 | 5 | 85 | 104 | UUUCUUAAGUCAAACUUUUC | CUAGGGUUUCAUUUAGGAAG | 1 |  | |
| sbi-miR1435b | SORBI_3006G238100 | 5 | 2056 | 2075 | UUUCUUAAGUCAAACCUUUU | UAUAGUUUUGACUUGAGUAC | Cleavage | 1 |  |
| sbi-miR156a | SORBI_3006G211701 | 5 | 1495 | 1514 | UGACAGAAGAGAGUGAGCAC | CUGCUCAUUUCCUUGUGUUG | 1 |  | |
| sbi-miR156b | SORBI_3006G211701 | 5 | 1495 | 1514 | UGACAGAAGAGAGUGAGCAC | CUGCUCAUUUCCUUGUGUUG | 1 |  | |
| sbi-miR156c | SORBI_3006G211701 | 5 | 1495 | 1514 | UGACAGAAGAGAGUGAGCAC | CUGCUCAUUUCCUUGUGUUG | 1 |  | |
| sbi-miR156f | SORBI_3006G211701 | 5 | 1495 | 1514 | UGACAGAAGAGAGUGAGCAC | CUGCUCAUUUCCUUGUGUUG | 1 |  | |
| sbi-miR156g | SORBI_3006G211701 | 5 | 1495 | 1514 | UGACAGAAGAGAGUGAGCAC | CUGCUCAUUUCCUUGUGUUG | 1 |  | |
| sbi-miR156h | SORBI_3006G211701 | 5 | 1495 | 1514 | UGACAGAAGAGAGUGAGCAC | CUGCUCAUUUCCUUGUGUUG | 1 |  | |
| sbi-miR156i | SORBI_3006G211701 | 5 | 1495 | 1514 | UGACAGAAGAGAGUGAGCAC | CUGCUCAUUUCCUUGUGUUG | 1 |  | |
| sbi-miR159b | SORBI_3006G172550 | 5 | 1498 | 1518 | CUUGGAUUGAAGGGAGCUCCU | UCGGAUUCACUUCAAUCCAUG | Cleavage | 1 |  |
| sbi-miR160f | SORBI_3005G046700 | 5 | 1258 | 1278 | UGCCUGGCUCCCUGAAUGCCA | AAAAAUGCAAGGAGCCAGGUA | Cleavage | 1 |  |
| sbi-miR171a | SORBI_3001G304201 | 5 | 77 | 97 | UGAUUGAGCCGUGCCAAUAUC | GUUAGUGGCUCGGCUCAAGCU | Cleavage | 1 |  |
| sbi-miR171b | SORBI_3001G304201 | 5 | 77 | 97 | UGAUUGAGCCGUGCCAAUAUC | GUUAGUGGCUCGGCUCAAGCU | Cleavage | 1 |  |
| sbi-miR171c | SORBI_3004G292250 | 5 | 760 | 780 | GAGGUGAGCCGAGCCAAUAUC | UAUGAUGACUCGGUUCAUUUG | Cleavage | 1 |  |
| sbi-miR171d | SORBI_3001G304201 | 5 | 77 | 97 | UGAUUGAGCCGUGCCAAUAUC | GUUAGUGGCUCGGCUCAAGCU | Cleavage | 1 |  |
| sbi-miR171g | SORBI_3001G304201 | 5 | 77 | 97 | UGAUUGAGCCGCGCCAAUAUC | GUUAGUGGCUCGGCUCAAGCU | Cleavage | 1 |  |
| sbi-miR171h | SORBI_3001G304201 | 5 | 77 | 97 | GGAUUGAGCCGCGUCAAUAUC | GUUAGUGGCUCGGCUCAAGCU | Cleavage | 1 |  |
| sbi-miR171h | SORBI_3006G172601 | 5 | 774 | 794 | GGAUUGAGCCGCGUCAAUAUC | UCUACUGAAGUGCUUCAAUCC | Cleavage | 1 |  |
| sbi-miR171i | SORBI_3001G304201 | 5 | 77 | 97 | UGAUUGAGCCGUGCCAAUAUC | GUUAGUGGCUCGGCUCAAGCU | Cleavage | 1 |  |
| sbi-miR171j | SORBI_3001G304201 | 5 | 77 | 97 | UGAUUGAGCCGCGCCAAUAUC | GUUAGUGGCUCGGCUCAAGCU | Cleavage | 1 |  |
| sbi-miR171k | SORBI_3001G304201 | 5 | 77 | 97 | UGAUUGAGCCGUGCCAAUAUC | GUUAGUGGCUCGGCUCAAGCU | Cleavage | 1 |  |
| sbi-miR395f | SORBI_3007G030100 | 5 | 237 | 257 | AUGAAGUGUUUGGGGGAACUC | CUGCUGCUACGGACACUUCAU | Cleavage | 1 |  |
| sbi-miR395k | SORBI_3007G030100 | 5 | 237 | 257 | GUGAAGUGUUUGGAGGAACUC | CUGCUGCUACGGACACUUCAU | Cleavage | 1 |  |
| sbi-miR397-3p | SORBI_3008G158600 | 5 | 1022 | 1042 | UCACCGGCGCUGCACUCAAUU | CAGUGGGGGCGGCACCGGUGG | Cleavage | 1 |  |
| sbi-miR397-3p | SORBI_3007G030100 | 5 | 684 | 704 | UCACCGGCGCUGCACUCAAUU | CGCUGGGCGUGGCGCUGGUGC | Cleavage | 1 |  |
| sbi-miR399d | SORBI_3001G112200 | 5 | 1549 | 1569 | UGCCAAAGGAGAGUUGCCCUG | UUGGGGAAUUUUCUUUUGACC | Cleavage | 1 |  |
| sbi-miR399i | SORBI_3001G112200 | 5 | 1549 | 1569 | UGCCAAAGGAGAGUUGCCCUG | UUGGGGAAUUUUCUUUUGACC | Cleavage | 1 |  |
| sbi-miR437a | SORBI_3001G304300 | 5 | 1550 | 1570 | AAAGUUAGAGAAGUUUGACUU | UAUUUAGCCUUUUCUAAUUUA | Cleavage | 1 |  |
| sbi-miR437a | SORBI_3004G292900 | 5 | 119 | 139 | AAAGUUAGAGAAGUUUGACUU | ACGCCAAACUUCUCUACCGUG | Cleavage | 1 |  |
| sbi-miR437b | SORBI_3001G304300 | 5 | 1550 | 1570 | AAAGUUAGAGAAGUUUGACUU | UAUUUAGCCUUUUCUAAUUUA | Cleavage | 1 |  |
| sbi-miR437b | SORBI_3004G292900 | 5 | 119 | 139 | AAAGUUAGAGAAGUUUGACUU | ACGCCAAACUUCUCUACCGUG | Cleavage | 1 |  |
| sbi-miR437c | SORBI_3001G304300 | 5 | 1550 | 1570 | AAAGUUAGAGAAGUUUGACUU | UAUUUAGCCUUUUCUAAUUUA | Cleavage | 1 |  |
| sbi-miR437c | SORBI_3004G292900 | 5 | 119 | 139 | AAAGUUAGAGAAGUUUGACUU | ACGCCAAACUUCUCUACCGUG | Cleavage | 1 |  |
| sbi-miR437d | SORBI_3001G304300 | 5 | 1550 | 1570 | AAAGUUAGAGAAGUUUGACUU | UAUUUAGCCUUUUCUAAUUUA | Cleavage | 1 |  |
| sbi-miR437d | SORBI_3004G292900 | 5 | 119 | 139 | AAAGUUAGAGAAGUUUGACUU | ACGCCAAACUUCUCUACCGUG | Cleavage | 1 |  |
| sbi-miR437e | SORBI_3001G304300 | 5 | 1550 | 1570 | AAAGUUAGAGAAGUUUGACUU | UAUUUAGCCUUUUCUAAUUUA | Cleavage | 1 |  |
| sbi-miR437e | SORBI_3004G292900 | 5 | 119 | 139 | AAAGUUAGAGAAGUUUGACUU | ACGCCAAACUUCUCUACCGUG | Cleavage | 1 |  |
| sbi-miR437f | SORBI_3001G304300 | 5 | 1550 | 1570 | AAAGUUAGAGAAGUUUGACUU | UAUUUAGCCUUUUCUAAUUUA | Cleavage | 1 |  |
| sbi-miR437f | SORBI_3004G292900 | 5 | 119 | 139 | AAAGUUAGAGAAGUUUGACUU | ACGCCAAACUUCUCUACCGUG | Cleavage | 1 |  |
| sbi-miR437g | SORBI_3001G304300 | 5 | 1550 | 1570 | AAAGUUAGAGAAGUUUGACUU | UAUUUAGCCUUUUCUAAUUUA | Cleavage | 1 |  |
| sbi-miR437g | SORBI_3004G292900 | 5 | 119 | 139 | AAAGUUAGAGAAGUUUGACUU | ACGCCAAACUUCUCUACCGUG | Cleavage | 1 |  |
| sbi-miR437i | SORBI_3001G304300 | 5 | 1550 | 1570 | AAAGUUAGAGAAGUUUGACUU | UAUUUAGCCUUUUCUAAUUUA | Cleavage | 1 |  |
| sbi-miR437i | SORBI_3004G292900 | 5 | 119 | 139 | AAAGUUAGAGAAGUUUGACUU | ACGCCAAACUUCUCUACCGUG | Cleavage | 1 |  |
| sbi-miR437j | SORBI_3001G304300 | 5 | 1550 | 1570 | AAAGUUAGAGAAGUUUGACUU | UAUUUAGCCUUUUCUAAUUUA | Cleavage | 1 |  |
| sbi-miR437j | SORBI_3004G292900 | 5 | 119 | 139 | AAAGUUAGAGAAGUUUGACUU | ACGCCAAACUUCUCUACCGUG | Cleavage | 1 |  |
| sbi-miR437k | SORBI_3001G304300 | 5 | 1550 | 1570 | AAAGUUAGAGAAGUUUGACUU | UAUUUAGCCUUUUCUAAUUUA | Cleavage | 1 |  |
| sbi-miR437k | SORBI_3004G292900 | 5 | 119 | 139 | AAAGUUAGAGAAGUUUGACUU | ACGCCAAACUUCUCUACCGUG | Cleavage | 1 |  |
| sbi-miR437l | SORBI_3001G304300 | 5 | 1550 | 1570 | AAAGUUAGAGAAGUUUGACUU | UAUUUAGCCUUUUCUAAUUUA | Cleavage | 1 |  |
| sbi-miR437l | SORBI_3004G292900 | 5 | 119 | 139 | AAAGUUAGAGAAGUUUGACUU | ACGCCAAACUUCUCUACCGUG | Cleavage | 1 |  |
| sbi-miR437m | SORBI_3001G304300 | 5 | 1550 | 1570 | AAAGUUAGAGAAGUUUGACUU | UAUUUAGCCUUUUCUAAUUUA | Cleavage | 1 |  |
| sbi-miR437m | SORBI_3004G292900 | 5 | 119 | 139 | AAAGUUAGAGAAGUUUGACUU | ACGCCAAACUUCUCUACCGUG | Cleavage | 1 |  |
| sbi-miR437n | SORBI_3001G304300 | 5 | 1550 | 1570 | AAAGUUAGAGAAGUUUGACUU | UAUUUAGCCUUUUCUAAUUUA | Cleavage | 1 |  |
| sbi-miR437n | SORBI_3004G292900 | 5 | 119 | 139 | AAAGUUAGAGAAGUUUGACUU | ACGCCAAACUUCUCUACCGUG | Cleavage | 1 |  |
| sbi-miR437o | SORBI_3001G304300 | 5 | 1550 | 1570 | AAAGUUAGAGAAGUUUGACUU | UAUUUAGCCUUUUCUAAUUUA | Cleavage | 1 |  |
| sbi-miR437o | SORBI_3004G292900 | 5 | 119 | 139 | AAAGUUAGAGAAGUUUGACUU | ACGCCAAACUUCUCUACCGUG | Cleavage | 1 |  |
| sbi-miR437p | SORBI_3001G304300 | 5 | 1550 | 1570 | AAAGUUAGAGAAGUUUGACUU | UAUUUAGCCUUUUCUAAUUUA | Cleavage | 1 |  |
| sbi-miR437p | SORBI_3004G292900 | 5 | 119 | 139 | AAAGUUAGAGAAGUUUGACUU | ACGCCAAACUUCUCUACCGUG | Cleavage | 1 |  |
| sbi-miR437q | SORBI_3001G304300 | 5 | 1550 | 1570 | AAAGUUAGAGAAGUUUGACUU | UAUUUAGCCUUUUCUAAUUUA | Cleavage | 1 |  |
| sbi-miR437q | SORBI_3004G292900 | 5 | 119 | 139 | AAAGUUAGAGAAGUUUGACUU | ACGCCAAACUUCUCUACCGUG | Cleavage | 1 |  |
| sbi-miR437r | SORBI_3001G304300 | 5 | 1550 | 1570 | AAAGUUAGAGAAGUUUGACUU | UAUUUAGCCUUUUCUAAUUUA | Cleavage | 1 |  |
| sbi-miR437r | SORBI_3004G292900 | 5 | 119 | 139 | AAAGUUAGAGAAGUUUGACUU | ACGCCAAACUUCUCUACCGUG | Cleavage | 1 |  |
| sbi-miR437s | SORBI_3001G304300 | 5 | 1550 | 1570 | AAAGUUAGAGAAGUUUGACUU | UAUUUAGCCUUUUCUAAUUUA | Cleavage | 1 |  |
| sbi-miR437s | SORBI_3004G292900 | 5 | 119 | 139 | AAAGUUAGAGAAGUUUGACUU | ACGCCAAACUUCUCUACCGUG | Cleavage | 1 |  |
| sbi-miR437t | SORBI_3001G304300 | 5 | 1550 | 1570 | AAAGUUAGAGAAGUUUGACUU | UAUUUAGCCUUUUCUAAUUUA | Cleavage | 1 |  |
| sbi-miR437t | SORBI_3004G292900 | 5 | 119 | 139 | AAAGUUAGAGAAGUUUGACUU | ACGCCAAACUUCUCUACCGUG | Cleavage | 1 |  |
| sbi-miR437u | SORBI_3001G304300 | 5 | 1550 | 1570 | AAAGUUAGAGAAGUUUGACUU | UAUUUAGCCUUUUCUAAUUUA | Cleavage | 1 |  |
| sbi-miR437u | SORBI_3004G292900 | 5 | 119 | 139 | AAAGUUAGAGAAGUUUGACUU | ACGCCAAACUUCUCUACCGUG | Cleavage | 1 |  |
| sbi-miR437v | SORBI_3001G304300 | 5 | 1550 | 1570 | AAAGUUAGAGAAGUUUGACUU | UAUUUAGCCUUUUCUAAUUUA | Cleavage | 1 |  |
| sbi-miR437v | SORBI_3004G292900 | 5 | 119 | 139 | AAAGUUAGAGAAGUUUGACUU | ACGCCAAACUUCUCUACCGUG | Cleavage | 1 |  |
| sbi-miR437w | SORBI_3001G304300 | 5 | 1550 | 1570 | AAAGUUAGAGAAGUUUGACUU | UAUUUAGCCUUUUCUAAUUUA | Cleavage | 1 |  |
| sbi-miR437w | SORBI_3004G292900 | 5 | 119 | 139 | AAAGUUAGAGAAGUUUGACUU | ACGCCAAACUUCUCUACCGUG | Cleavage | 1 |  |
| sbi-miR528 | SORBI_3006G211701 | 5 | 585 | 605 | UGGAAGGGGCAUGCAGAGGAG | GGCCGCGGCGUGCCCCUACUG | Cleavage | 1 |  |
| sbi-miR5385 | SORBI_3008G158600 | 5 | 1051 | 1073 | ACCACCAACCCCACC-GCUUCUC | GCGGAGCAGGUGGGGGUGGUGGA | Cleavage | 1 |  |
| sbi-miR5386 | SORBI_3004G291500 | 5 | 306 | 325 | CGUCGCUGUCGCGCGCGCUG | CAACGCCUGCGGCGGCGGCU | Cleavage | 1 |  |
| sbi-miR5388 | SORBI_3004G292900 | 5 | 253 | 274 | AUCUUUGCCGGGUGUCUCUGAC | AAAAAAAACACUCGACAAAGAA | Cleavage | 2 |  |
| sbi-miR5564a | SORBI_3006G211701 | 5 | 1993 | 2014 | UGGGGAAGCAAUUCGUCGAACA | GAAUCAUCGAAAUGUUUCUUCA | 1 |  | |
| sbi-miR5568b-3p | SORBI_3001G541300 | 5 | 278 | 298 | ACUAUGUAUCUAGAAAAGCUA | UAUUUUUUCUAUAUAUAUAAU | 1 |  | |
| sbi-miR5568b-5p | SORBI_3003G058900 | 5 | 1555 | 1575 | UUUCUAGGUACAUAGCUUUUG | UGAAGGCUCUGUCCUUGGGAA | Cleavage | 1 |  |
| sbi-miR5568c-5p | SORBI_3010G204700 | 5 | 346 | 366 | UCUGUUCCAAAUUGUAAGUCG | UAAUUGGAAAUUUGCAAUAGA | Cleavage | 1 |  |
| sbi-miR5568d-5p | SORBI_3001G302600 | 5 | 764 | 784 | UGGCUUUUCUAGAUACAUAGC | UUGAUGUGUUUUGAAAAUCCA | 1 |  | |
| sbi-miR5568e-3p | SORBI_3003G149800 | 5 | 101 | 121 | UAUCUAGAAAAGCUAAAACGU | AUGGUGUACCUUUGCUAGAUA | Cleavage | 1 |  |
| sbi-miR5568e-5p | SORBI_3004G291500 | 5 | 1217 | 1236 | GAUGUUUUGGGUUUUCUAGAU | GCCUA-AGAACUCGAAAUAUA | Cleavage | 1 |  |
| sbi-miR6218-3p | SORBI_3004G292900 | 5 | 876 | 896 | ACAAGUUUCGUGAUUUUUGGA | UCAAAAAGUUAUGAAACUUUG | Cleavage | 1 |  |
| sbi-miR6224a-3p | SORBI_3003G329400 | 5 | 808 | 828 | CUUAUAUACUAGGACGGAGGG | GAAUCGGUCCUAGUUUAGAAG | Cleavage | 1 |  |
| sbi-miR6224b-3p | SORBI_3003G329400 | 5 | 808 | 828 | CUUAUAUACUAGGACGGAGGG | GAAUCGGUCCUAGUUUAGAAG | Cleavage | 1 |  |
| sbi-miR6224c-3p | SORBI_3003G329400 | 5 | 808 | 828 | CUUAUAUACUAGGACGGAGGG | GAAUCGGUCCUAGUUUAGAAG | Cleavage | 1 |  |
| sbi-miR6227-3p | SORBI_3001G302600 | 5 | 2021 | 2042 | CUCACAACACUUGCUAUUUGGG | CUCUAAUAGUAAGUUUUGAGGG | Cleavage | 1 |  |
| sbi-miR6227-3p | SORBI_3001G304201 | 5 | 1958 | 1979 | CUCACAACACUUGCUAUUUGGG | UUGAAAUUGCAAUUGUGGUGAC | Cleavage |  | |
| sbi-miR6227-5p | SORBI_3010G054800 | 5 | 1044 | 1066 | GGGCCCAAAUAGCAAGUGUUGUGA | UAACUACACUUGC-AUUUGGGUUA | Cleavage |  | |
| sbi-miR6230-3p | SORBI_3010G204700 | 5 | 280 | 300 | UAACAAGUUUAGGGAUCUAGA | UAUAUAUUCUAAGAUUUGUUG | Cleavage |  | |
| sbi-miR6230-5p | SORBI_3004G291500 | 5 | 1119 | 1139 | UUUUGGGUCCCUAAACUUGUU | UCUGAUUUUAUUGACCCAAAA | Cleavage |  | |
| sbi-miR6231-3p | SORBI_3005G046700 | 5 | 1385 | 1405 | UAUUUGUGGACUCAUGGACAU | CGCUCAAUUGGUCUAUAAAUA | Cleavage | 1 |  |
| sbi-miR6232a-5p | SORBI_3001G302600 | 5 | 1138 | 1160 | GUCGCUUUGACUUUUUUGGUACAU | AUGU-CCAUGAAAGUUAAAGACAC | Cleavage | 1 |  |
| sbi-miR6232b-3p | SORBI_3003G058900 | 5 | 1447 | 1466 | AAUUCGAUGUACCAAAAAAGU | UGUUUUUUGG-GUGUUGAAUU | 1 |  | |
| sbi-miR6233-5p | SORBI_3003G126100 | 5 | 428 | 450 | UGUUGAGGCUGGAGCGAAACUCGG | UGCUGUUUCG-UUUAGUUUCGGCA | Cleavage | 1 |  |
| sbi-miR6235-3p | SORBI_3001G541300 | 5 | 1868 | 1891 | AACGAACAGUAUUUUUCUCUUACA | ACCAAAAAAAAGGUGCUGUUCUUU | Cleavage | 1 |  |
| sbi-miR821d | SORBI_3003G149800 | 5 | 847 | 867 | AAGUCAUCAACAACAAAGUUG | CGAUUUCUUUGUUCAUGACUA | Cleavage | 1 |  |
| sbi-miR821e | SORBI_3001G304201 | 5 | 437 | 457 | AAGUCAUCAAAAUAAAAGUUG | CUUUUUUUUUUUUGUUGAUUU | Cleavage | 1 |  |
