# Supplementary material for: Identification and analysis of proline-rich proteins and hybrid proline-rich proteins super family genes from Sorghum bicolor and their expression patterns to abiotic stress and zinc stimuli
Source: Front Plant Sci. 2022 Sep 26;13:952732. doi: 10.3389/fpls.2022.952732 (PMC9549341; doi:10.3389/fpls.2022.952732)
Supplement: Supplementary file 25 [file Table_13.doc]

**Table S13.** Number of acetylation and methylation sites in *SbPRPs* in sorghum

| Common name | No. of Acetylation sites | No. of Methylation sites | |
| --- | --- | --- | --- |
|  |  | Lysine | Arginine |
| SbPRP-1 | 41 | 36 | 0 |
| SbPRP-2 | 14 | 10 | 0 |
| SbPRP-3 | 17 | 5 | 0 |
| SbPRP-4 | 15 | 6 | 0 |
| SbPRP-5 | 9 | 4 | 1 |
| SbPRP-6 | 8 | 2 | 0 |
| SbPRP-7 | 9 | 4 | 1 |
| SbPRP-8 | 0 | 7 | 9 |
| SbPRP-9 | 80 | 0 | 0 |
| SbPRP-10 | 30 | 62 | 10 |
| SbPRP-11 | 16 | 18 | 0 |
| SbPRP-12 | 11 | 7 | 9 |
| SbPRP-13 | 5 | 4 | 5 |
| SbPRP-14 | 7 | 7 | 1 |
| SbPRP-15 | 8 | 5 | 7 |
| SbPRP-16 | 15 | 7 | 0 |
| SbPRP-17 | 29 | 7 | 1 |
| SbPRP-18 | 4 | 22 | 9 |
| SbPRP-19 | 21 | 31 | 1 |
| SbPRP-20 | 4 | 3 | 0 |
| SbPRP-21 | 60 | 19 | 4 |
