# Supplementary material for: Identification and analysis of proline-rich proteins and hybrid proline-rich proteins super family genes from Sorghum bicolor and their expression patterns to abiotic stress and zinc stimuli
Source: Front Plant Sci. 2022 Sep 26;13:952732. doi: 10.3389/fpls.2022.952732 (PMC9549341; doi:10.3389/fpls.2022.952732)
Supplement: Supplementary file 26 [file Table_14.doc]

**Table S14.** Number of acetylation and methylation sites in *SbHyPRPs* in sorghum

| Common name | No. of Acetylation sites | No. of methylation sites | |
| --- | --- | --- | --- |
|  |  | Lysine | Arginine |
| SbHyPRP-1 | 5 | 1 | 0 |
| SbHyPRP-2 | 6 | 3 | 1 |
| SbHyPRP-3 | 3 | 1 | 0 |
| SbHyPRP-4 | 7 | 2 | 1 |
| SbHyPRP-5 | 6 | 2 | 0 |
| SbHyPRP-6 | 3 | 0 | 1 |
| SbHyPRP-7 | 5 | 2 | 0 |
| SbHyPRP-8 | 8 | 6 | 4 |
| SbHyPRP-9 | 15 | 6 | 6 |
| SbHyPRP-10 | 5 | 2 | 2 |
| SbHyPRP-11 | 35 | 17 | 3 |
| SbHyPRP-12 | 12 | 4 | 0 |
| SbHyPRP-13 | 7 | 1 | 0 |
| SbHyPRP-14 | 3 | 1 | 0 |
| SbHyPRP-15 | 3 | 1 | 0 |
| SbHyPRP-16 | 22 | 12 | 1 |
| SbHyPRP-17 | 2 | 0 | 0 |
| SbHyPRP-18 | 6 | 2 | 0 |
| SbHyPRP-19 | 11 | 4 | 0 |
| SbHyPRP-20 | 8 | 7 | 0 |
| SbHyPRP-21 | 15 | 7 | 1 |
| SbHyPRP-22 | 11 | 5 | 1 |
| SbHyPRP-23 | 4 | 1 | 1 |
| SbHyPRP-24 | 3 | 1 | 0 |
| SbHyPRP-25 | 3 | 1 | 1 |
| SbHyPRP-26 | 4 | 3 | 0 |
| SbHyPRP-27 | 15 | 8 | 0 |
