# Supplementary material for: Identification and analysis of proline-rich proteins and hybrid proline-rich proteins super family genes from Sorghum bicolor and their expression patterns to abiotic stress and zinc stimuli
Source: Front Plant Sci. 2022 Sep 26;13:952732. doi: 10.3389/fpls.2022.952732 (PMC9549341; doi:10.3389/fpls.2022.952732)
Supplement: Supplementary file 27 [file Table_15.docx]

| Gene | Chromosome | Count | Motif | Motif length | Length |
| --- | --- | --- | --- | --- | --- |
| *SbPRP1* | 1 | 1 | (TGCA)3 | 4 | 33-44 |
| *SbPRP6* | 1 | 3 | (AAG)4 | 3 | 469-480 |
|  |  |  | (CCG)6 | 3 | 496-513 |
|  |  |  | (CGA)4 | 3 | 696-707 |
| *SbPRP7* | 1 | 1 | (GGTGCT)3 | 6 | 21-38 |
| *SbPRP8* | 3 | 2 | (CGG)5 | 3 | 69-83 |
|  |  |  | (CCGCGC)3 | 6 | 737-754 |
| *SbPRP10* | 3 | 2 | (TGA)4 | 3 | 1215-1226 |
|  |  |  | (CGA)6 | 3 | 7392-7409 |
| *SbPRP13* | 5 | 1 | (CAG)6 | 3 | 1414-1431 |
| *SbPRP15* | 6 | 1 | (CGGCAG)4 | 6 | 267-290 |
| *SbPRP16* | 6 | 1 | (CCG)6 | 3 | 103-120 |
| *SbPRP17* | 6 | 1 | (TACG)4 | 4 | 721-736 |
| *SbPRP18* | 7 | 3 | (GGC)4 | 3 | 721-732 |
|  |  |  | (CAG)9 | 3 | 2938-2964 |
|  |  |  | (CTG)4 | 3 | 3018-3029 |
| *SbPRP20* | 10 | 1 | (GTG)4 | 3 | 116-127 |
| *SbPRP21* | 10 | 1 | (AAG)4 | 4 | 1163-1174 |
| *SbHyPRP1* | 1 | 2 | (CGG)4 | 3 | 243-254 |
|  |  |  | (GGT)4 | 3 | 349-360 |
| *SbHyPRP2* | 1 | 1 | (CGC)4 | 3 | 125-136 |
| *SbHyPRP4* | 1 | 2 | (GCC)4 | 3 | 55-66 |
|  |  |  | (CGGCGC)3 | 6 | 95-112 |
| *SbHyPRP6* | 1 | 2 | (TGG)4 | 3 | 156-167 |
|  |  |  | (GGC)9 | 3 | 229-255 |
| *SbHyPRP7* | 1 | 1 | (GCG)4 | 3 | 143-154 |
| *SbHyPRP11* | 3 | 1 | (CCCTCC)5 | 6 | 144-173 |
| *SbHyPRP12* | 3 | 1 | (GCA)6 | 3 | 246-263 |
| *SbHyPRP14* | 4 | 1 | (TCG)4 | 3 | 100-111 |
| *SbHyPRP15* | 4 | 1 | (TCG)4 | 3 | 100-111 |
| *SbHyPRP20* | 6 | 1 | (CCG)6 | 3 | 103-120 |
| *SbHyPRP21* | 6 | 1 | (TACG)4 | 4 | 721-736 |
| *SbHyPRP22* | 7 | 1 | (GGTGGC)3 | 6 | 2-20 |
| *SbHyPRP25* | 10 | 1 | (CTA)7 | 3 | 371-398 |
| *SbHyPRP26* | 10 | 1 | (GTG)4 | 3 | 116-127 |
| *SbHyPRP27* | 10 | 1 | (TCGCCG)3 | 6 | 184-201 |

**Table S15.** Simple sequence repeats (SSRs) present in SbPRP and SbHyPRP gene transcripts
